# Functional cooperativity between the trigger factor chaperone and the ClpXP proteolytic complex

**DOI:** 10.1038/s41467-020-20553-x

**Published:** 2021-01-12

**Authors:** Kamran Rizzolo, Angela Yeou Hsiung Yu, Adedeji Ologbenla, Sa Rang Kim, Haojie Zhu, Koichiro Ishimori, Guillaume Thibault, Elisa Leung, Yi Wen Zhang, Mona Teng, Marta Haniszewski, Noha Miah, Sadhna Phanse, Zoran Minic, Sukyeong Lee, Julio Diaz Caballero, Mohan Babu, Francis T. F. Tsai, Tomohide Saio, Walid A. Houry

**Affiliations:** 1 Department of Biochemistry, University of Toronto, Toronto, ON M5G 1M1 Canada; 2 Graduate School of Chemical Sciences and Engineering, Hokkaido University, Sapporo, Hokkaido 060-8628 Japan; 3 Department of Chemistry, Faculty of Science, Hokkaido University, Sapporo, Hokkaido 060-0810 Japan; 4 School of Biological Sciences, Nanyang Technological University, Singapore, 637551 Singapore; 5 The Donnelly Centre, University of Toronto, Toronto, ON M5S 3E1 Canada; 6 Department of Biochemistry, University of Regina, Regina, Saskatchewan S4S 0A2 Canada; 7 Department of Chemistry and Biomolecular Science, University of Ottawa, John L. Holmes, Mass Spectrometry Facility, Ottawa, ON K1N 1A2 Canada; 8 Department of Biochemistry and Molecular Biology, Baylor College of Medicine, Houston, TX 77030 USA; 9 Department of Cell and Systems Biology, University of Toronto, Toronto, ON M5S 3G5 Canada; 10 Department of Molecular and Cellular Biology, Baylor College of Medicine, Houston, TX 77030 USA; 11 Department of Molecular Virology and Microbiology, Baylor College of Medicine, Houston, TX 77030 USA; 12 Institute of Advanced Medical Sciences, Tokushima University, Tokushima, 770-8503 Japan; 13 Department of Chemistry, University of Toronto, Toronto, ON M5S 3H6 Canada; 14 Present Address: Dewpoint Therapeutics, 6 Tide Street, Boston, MA 02210 USA; 15 Present Address: Pfizer Inc., 401 N Middletown Rd, Pearl River, NY 10965 USA

**Keywords:** Protein quality control, Chaperones

## Abstract

A functional association is uncovered between the ribosome-associated trigger factor (TF) chaperone and the ClpXP degradation complex. Bioinformatic analyses demonstrate conservation of the close proximity of *tig*, the gene coding for TF, and genes coding for ClpXP, suggesting a functional interaction. The effect of TF on ClpXP-dependent degradation varies based on the nature of substrate. While degradation of some substrates are slowed down or are unaffected by TF, surprisingly, TF increases the degradation rate of a third class of substrates. These include λ phage replication protein λO, master regulator of stationary phase RpoS, and SsrA-tagged proteins. Globally, TF acts to enhance the degradation of about 2% of newly synthesized proteins. TF is found to interact through multiple sites with ClpX in a highly dynamic fashion to promote protein degradation. This chaperone–protease cooperation constitutes a unique and likely ancestral aspect of cellular protein homeostasis in which TF acts as an adaptor for ClpXP.

## Abstract (web summary)

ClpXP is the main ATP-dependent proteolytic complex in bacteria, is essential for maintaining cellular protein homeostasis and is also critical for bacterial pathogenesis. Here, the authors establish a functional link between ClpXP and trigger actor, a chaperone involved in the early stages of protein folding.

## Introduction

In the very crowded environment of the cell, molecular chaperones and proteases function to ensure proper protein homeostasis either by promoting the folding of newly synthesized proteins and maintaining the correct conformation of pre-existing proteins or by degrading misfolded and unfolded proteins, respectively. Generally, some chaperones such as trigger factor (TF) and the Hsp70 system (DnaK/DnaJ/GrpE) act early during the folding process, while chaperonins (GroEL/GroES), and Hsp90 (HtpG) act at later stages of folding^1^. In addition, there is redundancy in the function of folding chaperones such as that found for TF and DnaK in folding newly synthesized proteins^2,3^.

**Fig. 1:**
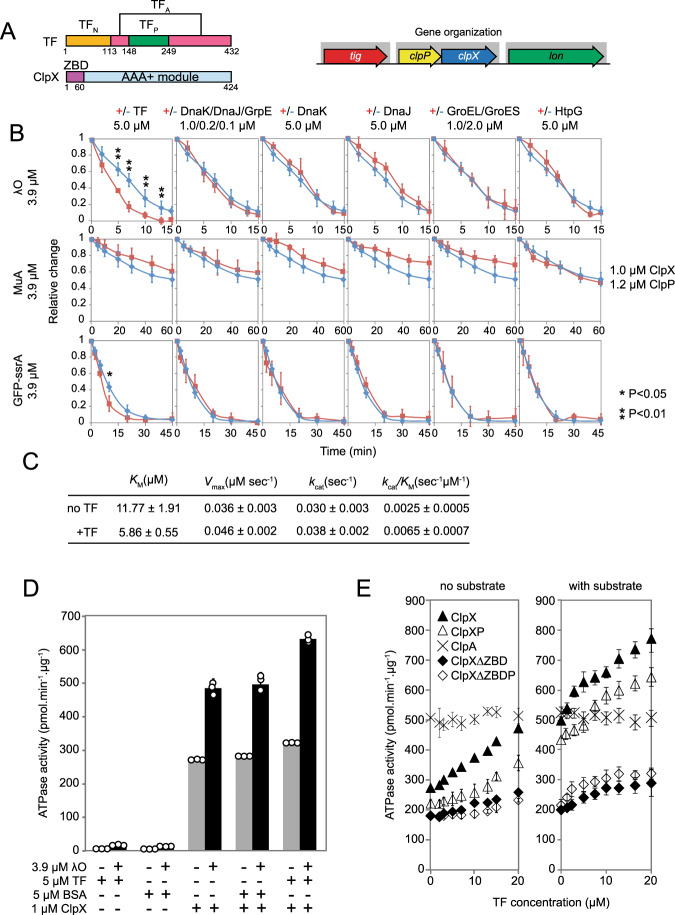
The effect of chaperones on the ClpXP-mediated degradation of model substrates. **A** Domains in *E. coli* ClpX and TF proteins (left panel) and the genetic localization of the *tig*, *clpPX* and *lon* operons in *E. coli* (right panel). **B** Kinetics of the degradation of model substrates by ClpXP in the presence (red) or absence (blue) of different chaperones. Shown are examples of at least three repeats; data are presented as mean values and error bars represent standard deviations. Student’s two-tailed *t* test was used to compare each time point. **C** Michaelis–Menten values derived from Supplementary Fig. 3c based on the change in the ClpXP-dependent degradation rates upon increasing concentrations of λO substrate. **D** Shown is the ATPase activities of ClpX in the absence and presence of λO substrate, TF, or BSA control. All reactions were background-subtracted and repeated at least three times to obtain the averages and standard deviations. ClpP is absent in these reactions. **E** The ATPase activities of ClpX and ClpXΔZBD in the absence or presence of λO substrate and increasing concentration of TF. The effect of TF on the ATPase activity of ClpA is shown as a control. Error bars represent the standard deviations from *n* = 3 independent replicates. Source data are provided as a Source Data file.

*Escherichia coli* TF is a 48 kDa protein containing an N-terminal ribosome-binding domain (TF_N_), a peptidyl-prolyl cis/trans isomerase (PPIase) domain (TF_P_), and a discontinuous domain with two arm motifs (TF_A_) (Fig. 1a)^4^. TF is highly abundant with an estimated concentration of ~50 µM in the cell^5^; it exists as a monomer and as a dimer. As a monomer, TF engages the ribosome in a 1:1 stoichiometry binding next to the exit tunnel of the 50 S large ribosomal subunit. This allows TF to interact with the nascent chain as it emerges from the ribosomal exit tunnel^6,7^. As a dimer, TF assists in various folding processes and has anti-aggregation activity^8^.

ClpXP is one of the main ATP-dependent protein degradation complexes in bacteria. It plays an essential role in protein quality control by removing misfolded, damaged, and regulatory proteins. The proteolytic complex consists of the tetradecameric serine protease ClpP^9^ and the hexameric ClpX unfoldase belonging to the AAA+ superfamily^10,11^. Substrate recognition by the ClpXP system occurs via specific recognition motifs, known as degrons, which are typically located at the N- or C-terminal ends of protein substrates. Some degrons are introduced by a specialized SsrA-tagging system which rescues stalled ribosomes^12^. Alternatively, adaptor proteins aid in the recruitment of substrates by tethering selected substrates to ClpX for degradation^13^. ClpX has two domains (Fig. 1a), an N-terminal zinc-binding domain (ZBD) and a C-terminal AAA+ domain that contains the characteristic Walker A and Walker B nucleotide binding and recognition motifs. ClpX binds to target substrates and uses the energy derived from ATP hydrolysis to unfold and thread substrates into the ClpP chamber through axial pores present at opposite ends of the ClpP cylinder. Substrates are generally cleaved into peptides of about 7–8 residues that exit the ClpP chamber through transient equatorial side pores^14^.

In phylogenetic studies of chaperones, we were intrigued to find that *tig*, the gene encoding the TF chaperone, was frequently found clustered with the *clpPX* operon in many species throughout the bacterial kingdom (Fig. 1a, Supplementary Fig. 1). This observation prompted us to seek out the functional link between TF and the ClpXP system. Unexpectedly, TF was found to enhance the ClpXP-dependent degradation of several substrates including the phage protein λO, the stationary phase sigma factor RpoS, and SsrA-tagged proteins both in vitro and in vivo. Based on results from biochemical and structural analyses, we propose that TF binds to ClpX by anchoring onto the AAA+ domain of the chaperone complex, thereby engaging the ZBD domain and modulating its movement towards the ClpP chamber. In doing so, TF accelerates the degradation of substrates. Our results suggest that TF facilitates ClpXP-mediated degradation of a select group of proteins and, hence, acts as an adaptor of the ClpXP system. This interplay between a folding chaperone and a major proteolysis complex was unexpected and provides new insights into bacterial proteostasis.

## Results

### Phylogenetic and genome analyses suggest a functional association between TF and ClpXP

In *E. coli*, *clpP* and *clpX* genes form the *clpPX* operon, while the *tig* gene constitutes its own operon that is located immediately upstream of the *clpPX* operon (Fig. 1a, Supplementary Fig. 1). In certain bacteria such as Bacillaceae, Staphylococcaceae, and Lactobacillaceae within the Firmicutes phyla and Gloeobacteraceae within the Cyanobacteria phyla, *clpX* and *clpP* are located at different loci but the *tig* gene is directly upstream of *clpP* (Supplementary Fig. 1). This initial observation prompted us to explore the genomic positioning of *tig*, *clpP*, and *clpX* in all available bacterial genomes. NCBI representative species and each selected genus were mapped onto the tree of life using the interactive tree of life tool^15^ (Supplementary Fig. 1). While all three genes are highly conserved and present throughout the bacterial kingdom; *clpP* and *clpX* were the only ones missing in Tenericutes. In *Dehalococcoides sp*., *clpP* is present without *clpX*, but *clpA* is found instead. In addition, we noticed that the *lon* gene encoding the Lon protease was also typically found close to *clpP* and *clpX* and always positioned downstream of them; so, we included *lon* in our analysis as well (Supplementary Fig. 1).

In general, the four genes *tig, clpP, clpX* and *lon* are almost always located next to each other in the order, *tig-clpP-clpX-lon*, and this cluster is conserved in Proteobacteria especially in species belonging to the Delta-, Beta-, and Gamma-classes (Supplementary Fig. 1). Some bacteria have *tig* next to *clpX* (*tig-clpX*), while the majority feature *tig* next to *clpP* (*tig-clpP*). Only in a small number of bacteria, the three genes are located far apart from each other, namely in *Chlorobiaceae* and *Rickettsiales* families, and in some members of Firmicute phyla. *lon* is also conserved but absent in the Staphylococcaceae, Streptococcaceae, and Mycobacteriaceae families^16^. Most notably, *lon* is clustered next to *tig* (*tig-lon*) in the ClpXP-lacking Mycoplasmataceae family.

Genes that are clustered together on the genome tend to have similar functions, are targeted to the same pathways, or are expressed in the same compartment of the cell^17^. Furthermore, the evolutionary conservation of such clustering is strong evidence for functional association^18^. Hence, the conserved genomic proximity led us to investigate potential functional and physical interactions between TF and ClpXP.

### TF modulates the ClpXP-dependent degradation rates of substrates

In order to test whether there is a functional relationship between TF and the ClpXP system, we conducted an in vitro degradation assay screen looking for the effect of TF on the rates of ClpXP-mediated degradation of three well-established ClpXP substrates: λO phage replication protein^19^, MuA phage transposase^20^, and GFP-SsrA^21^. In addition to TF, we also used the major *E. coli* chaperones DnaK/DnaJ/GrpE together, DnaK, DnaJ, GroEL/GroES and HtpG. DnaK and TF are known to have overlapping functions and recognize similar substrates^2,3,22^. Hence, DnaK serves as a control for the effect of TF on ClpXP-mediated degradation.

We observed three outcomes in our assays. First, for GFP-ssrA and λO substrates with DnaK/DnaJ/GrpE, DnaK, DnaJ, GroEL/GroES, and HtpG chaperones, no significant effect of the chaperone(s) on the degradation rates was observed (Fig. 1b, Supplementary Fig. 2a). TF also had no effect on FlhDC and TnaA (Supplementary Fig. 2b, c), two known substrates of ClpXP^23,24^. Second, the presence of chaperones slowed down the degradation rate as generally observed for reactions involving MuA, suggesting that chaperones interact with MuA and protect it from ClpXP degradation (Fig. 1b, Supplementary Fig. 2a). The same effect was seen with TF and the antitermination λ phage protein, λN-His (Supplementary Fig. 2d), and the fumarate nitrate reduction regulatory protein, Fnr (Supplementary Fig. 2e). Third, TF caused a significant increase in the rate of ClpXP-mediated degradation of λO. In addition to λO, we noticed a slight increase in the rate of GFP-ssrA degradation upon TF addition (Fig. 1b, Supplementary Fig. 2a). It should be noted that TF itself is not a substrate of ClpXP or ClpX∆ZBDP in the absence or presence of other ClpXP substrates (Supplementary Fig. 2f). ClpXΔZBD (ClpX AAA+) lacks the ZBD domain that is required for λO degradation^25^.

If TF strictly acts as a folding or holding chaperone in these assays, we would expect it to either have no effect or to protect the substrate from degradation by ClpXP, hence reducing the degradation rate. Alternatively, it is conceivable that TF might prevent the formation of small aggregates making substrates more accessible to ClpX and accelerating their degradation by ClpXP. However, there is no evidence of λO or GFP-SsrA aggregation under the conditions of our experiments. Hence, the enhancement of λO and GFP-SsrA ClpXP-dependent degradation is a unique result and suggests a functional association between the folding chaperone and the degradation complex for specific substrates.

To obtain a quantitative measure of the effect of TF on ClpXP-mediated degradation of λO, we took advantage of the higher number of intrinsic tryptophan residues within the λO protein sequence compared to other proteins in the reaction (λO: nine tryptophans, ClpX: none, ClpP: none and TF: one) and used this to measure the intrinsic tryptophan fluorescence change as a proxy for λO degradation. λO fluorescence decreased upon its degradation by ClpXP + ATP or the unspecific protease Proteinase K (Supplementary Fig. 3a). No significant change in fluorescence was observed for λO in the presence of TF alone plus or minus ATP, ClpXP without ATP, ClpXΔZBDP + ATP, or ClpX(E185Q)P + ATP (Supplementary Fig. 3a). ClpX(E185Q) has a mutation in the Walker B motif rendering it ATPase inactive^26^.

Subsequently, the degradation of λO by ClpXP + ATP was monitored by fluorescence in the presence of increasing concentrations of TF. As shown in Fig. S3B, the initial degradation rate of λO was enhanced in a TF-dependent manner. The titration curves yielded a *K*_d_ of 49 µM. In addition, by using increasing concentrations of the λO substrate in the presence of 1 µM ClpX, 1.2 µM ClpP, and 5 µM TF, we were able to determine the *K*_M_ and *k*_cat_ values for the degradation reaction (Fig. 1c, Supplementary Fig. 3c). TF reduced the *K*_M_ for this substrate by a factor of about 2 and increased the *k*_cat_ 1.3-fold. Hence, TF was able to increase the catalytic efficiency (*k*_cat_/*K*_M_) of ClpXP for λO by a factor of 2.6 indicating that it increases ClpXP selectivity toward λO (Fig. 1c).

Based on the above results, it is reasonable to expect that TF might modulate the ATPase activity of ClpX. It is well established that the ATPase activity of the chaperone is affected by ClpX adaptor proteins, substrates, and ClpP binding^25,27^. The presence of 5 μM TF stimulated the ATPase of 1 μM ClpX by about 20% compared to ClpX alone, while the control protein, bovine serum albumin, had no significant effect on the ClpX ATPase. In the presence of 3.9 µM of the λO substrate, we observe a 1.5- to 2-fold increase of ATPase activity with the highest activity produced when TF is present (Fig. 1d). In titration experiments, TF enhanced the ATPase activity of ClpX and ClpXP in the absence and presence of substrate and to a lesser extent that of ClpX∆ZBD, and ClpX∆ZBDP (Fig. 1e). Importantly, TF did not affect the ATPase activity of the related AAA + protein ClpA (Fig. 1e). These results show that TF stimulates the ATPase activity of ClpX mainly via the ZBD and this effect is independent of the ATPase increase produced by the λO substrate.

The TF single-domain (TF_N_, TF_P_, and TF_A_) and two-domain constructs (TF_NP_, TF_NA_, and TF_PA_) did not have a significant effect on the ClpXP-dependent degradation of λO (Supplementary Fig. 4a). Furthermore, they did not enhance the ATPase activity of ClpX, ClpX∆ZBD, or ClpA (Supplementary Fig. 4b), indicating that full-length TF is required for such an effect on ClpX.

### λO degradation is compromised in *tig* deletion mutant strain

**Fig. 2:**
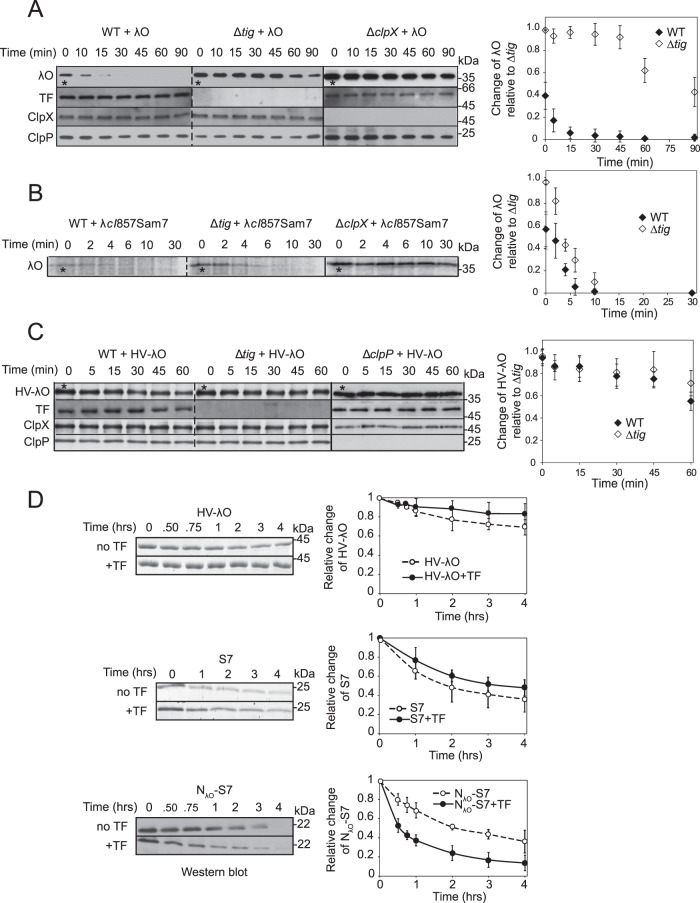
The role of TF in the cellular degradation of λO. **A** Western blot analysis of the degradation of overexpressed λO as a function of time after inhibition of protein synthesis in MC4100 WT, ∆*tig*, and ∆*clpX* cells. About 10^5^ cells were loaded for each time point. Reactions separated by dashed lines are from the same gel, while those separated by solid lines are from different gels. Curves on the right show the quantification of the bands. The error bars calculated here and in (**B**–**D**), are the standard deviation based on three independent experimental repeats. The stars highlight the zero time point directly after inhibition of protein synthesis. **B** MC4100 WT and Δ*tig* lysogens carrying heat-inducible prophage λ*cI*857Sam7 were heated at 42 °C for 8 min to induce phage protein expression. A pulse-chase experiment followed by immunoprecipitation of λO protein was performed to monitor endogenous λO degradation over time. Quantification of the λO band as a function of time is shown on the right. **C** Same as in A but using N-terminal HV- tagged λO. **D** In vitro degradation assay of HV-λO, S7, and N_λO_-S7 by ClpXP in the absence or presence of TF. Quantifications of the substrate bands are shown on the right. Western blots were performed for N_λO_-S7 (22 kDa) using an α-S7 antibody (dilution 1:2000) due to the close proximity in its migration to ClpP (23 kDa) on SDS-PAGE gels. Source data are provided as a Source Data file.

Having demonstrated that TF enhances λO and GFP-ssrA degradation by ClpXP in vitro, we carried out experiments to assess whether such an enhancement also occurs in vivo. The degradation of λO expressed from a plasmid was measured in WT and *tig* knockout *E. coli* cells. λO protein expression from a plasmid was induced by IPTG in both strains. At OD_600_ ~1.5, protein levels of ClpX, ClpP, TF, and overexpressed λO in WT strain were as follows: 16,000, 20,000, 44,000, and 390,000 molecules/cell, respectively. To measure the degradation of λO in vivo, λO was first induced for two hours then translation was inhibited using 30 µg/mL of tetracycline. Whole cell lysates obtained at different time points after tetracycline addition were separated by sodium dodecyl sulphate-polyacrylamide gel electrophoresis (SDS-PAGE) and proteins were visualized by western blot analysis. Degradation of λO occurred at a faster rate in WT cells compared to Δ*tig* cells (Fig. 2a), which is consistent with the in vitro results showing that TF enhances λO degradation (Fig. 1b, Supplementary Figs. 2a and 3). ClpXP levels remain unchanged during the time course. No significant degradation of λO was observed in Δ*clpX* cells, demonstrating that ClpXP is the main proteolytic system responsible for λO degradation (Fig. 2a). Furthermore, λO steady state protein level was significantly higher in Δ*tig* strain compared to WT cells (zero time point indicated by a star in Fig. 2a) demonstrating the rapid turnover of this protein by ClpXP. Importantly, the absence of TF in the ∆*tig* strain did not have any significant effect on protein levels of other chaperones in the cell (Supplementary Fig. 5a), and there was no change in the rate of λO degradation in the absence of another chaperone-like HtpG (∆*htpG*, Supplementary Fig. 5b). Thus, TF enhances the ClpXP-mediated degradation of the λO substrate in a highly specific manner.

In parallel experiments, λO stability was analyzed under more physiological conditions in WT, Δ*tig*, and Δ*clpX* lysogens carrying λcI857Sam7 prophage^28^. Phage replication was induced by heat shock. Cells were then pulse-labeled with radioactive methionine and chased with nonradioactive methionine. λO protein level was monitored by immunoprecipitating the protein with anti-λO antibody from samples at the indicated time points during the chase period and then visualized by autoradiography (Fig. 2b). Consistent with the results for λO overexpressed from a plasmid, λO levels in the lysogen were lower in WT compared to Δ*tig* lysogens at the early time points. λO was not degraded in the ∆*clpX* lysogen (Fig. 2b).

### The effect of TF on λO degradation is dependent on the presence of an exposed N-terminal ClpX-recognition sequence

It is known that the N-terminus of λO is required for the protein to be recognized by ClpXP^19^. Consistent with this, λO expressed from a plasmid with an N-terminal His-tag followed by a tobacco etch virus (TEV) protease cut site (HV-tag) is stable against degradation by ClpXP in vivo in the absence or presence of TF (Fig. 2c). As a result, TF cannot induce the ClpXP-dependent degradation of a substrate not exposing a ClpX recognition motif. Furthermore, in contrast to what was observed for λO, the levels of HV-λO at the zero-time point were the same in WT and Δ*tig* strains, which strongly suggests that TF promotes the rapid degradation of newly synthesized λO protein. We address this observation below.

We further tested the requirement of the λO N-terminus for its ClpXP-dependent degradation in vitro. Consistent with in vivo results, there was no degradation of the HV-λO substrate in the absence or presence of the TF chaperone (Fig. 2d). To establish that the TF effect is exerted through the N-terminus of λO (N_λO_, residues 1–12), we fused N_λO_ to the N-terminus of a poor ClpXP substrate, the 30S ribosomal protein S7. The degradation of S7 by ClpXP is slowed down in the presence of TF both in vitro and in vivo (Fig. 2d, Supplementary Fig. 5c). Similarly, a fusion of N_λO_ with S7 (N_λO_-S7) was still a poor ClpXP substrate and was degraded at a similar rate as S7 alone; however, N_λO_-S7 degradation in vitro was enhanced by about tenfold in the presence of TF (Fig. 2d). This demonstrates that an exposed ClpX-recognition sequence in the substrate is critical for enhancement of its degradation by TF.

### TF enhances the ClpXP-dependent degradation of λO by promoting the downward movement of the ZBD dimer (ZBD_2_) towards ClpP

**Fig. 3:**
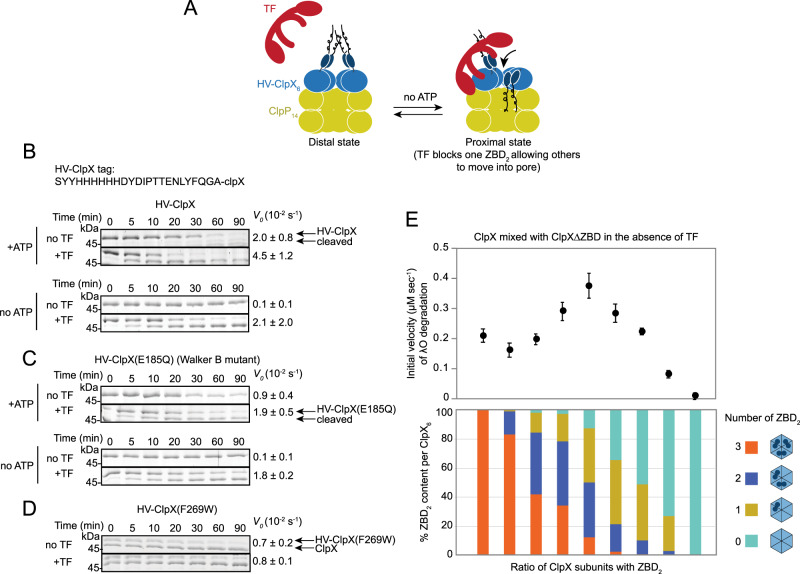
TF enhances ClpXP-dependent degradation by modulating the movement of ZBD. **A** Model of the effect of TF on the change in the configuration of ZBD in ClpXP. Upon TF binding to a ZBD_2_, we propose that unbound ZBD_2_ move from a distal configuration away from the ClpP catalytic chamber to a proximal configuration closer to the ClpP chamber. Hence, TF binding favors the proximal state of the unbound ZBD_2_s. **B** The enhanced clipping and degradation of N-terminally tagged ClpX by ClpP in the presence of TF. In vitro degradation assays using N-terminally tagged ClpX, HV-ClpX, in the absence or presence of TF and ATP. The HV-ClpX tag sequence is shown on the top. *V*_0_ refers to the initial rate of disappearance of HV-ClpX. Standard deviations here and in (**C**, **D**) were calculated from at least three independent experiments. **C** Same as in (**B**) but using the ATPase-deficient Walker B HV-ClpX mutant. **D** Degradation assay in which the ClpXP complex was performed in the presence of ATP, then HV-ClpX(F269W) was added to the mixture. The rates shown are for the HV-ClpX(F269W) protein degradation. **E** Initial rates of λO degradation in the presence of mixed ClpX:ClpX∆ZBD complexes and ClpP. The calculated ratio of ClpX and ClpX∆ZBD in each ClpX_6_ complex varies depending on the mixing ratio and is shown in the lower panel (see “Methods”). Error bars represent the standard deviations from *n* = 3 independent reactions. Source data are provided as a Source Data file.

We had earlier demonstrated that ZBD_2_ undergoes a nucleotide-dependent movement between two conformational states, one of which positions ZBD_2_ away from ClpP and on top of the AAA+ ring, while the other state positions ZBD_2_ closer to ClpP (Fig. 3a)^29^. We named these two states distal and proximal. This movement of ZBD_2_ was also modulated by the adaptor SspB. We argued that this ZBD_2_ movement promotes substrate translocation and subsequent degradation by ClpP. Therefore, we tested whether TF modulates this movement.

We took advantage of an HV-tagged ClpX construct (refer to Fig. 3b for sequence) that we had previously used as a proxy for ZBD_2_ movements^29^. In these assays, a lower molecular weight ClpX is produced when HV-ClpX is clipped by bound ClpP in active ClpXP complexes in the presence of ATP^29^. Interestingly, this clipping of HV-ClpX was drastically enhanced by TF (Fig. 3b). With the addition of ClpP and ATP, the rate of disappearance of HV-ClpX was >2× faster in the presence of TF than in its absence (Fig. 3b). Interestingly, the enhanced clipping of the HV-ClpX by TF was seen even in the absence of ATP and was further confirmed using the ATPase-deficient HV-ClpX(E185Q) Walker B mutant^26^ (Fig. 3b, c). A ClpX mutant unable to bind to ClpP, HV-ClpX(F269W)^30^, was not significantly degraded or clipped in the presence of TF and wild-type ClpXP (Fig. 3d), demonstrating that clipping is not from free HV-ClpX^29^. In our previous study, we found that mutations in two conserved proline residues (P64A and P66A) located between the ZBD and the AAA+ domains of ClpX affect nucleotide-dependent changes transmitted from the AAA+ to the ZBD^29^. Hence, we checked if the enhancement of clipping by TF was through these proline residues (Supplementary Fig. 5d). Our results show that TF was still able to enhance HV-ClpX(P64A/P66A) clipping, suggesting a mechanism that does not act through these residues and, importantly, that the PPIase activity of TF is not needed for this activity. Together, our results show that TF promotes the movement of ZBD_2_ from the distal to the proximal state even in the absence of ATP (Fig. 3a).

We hypothesize that TF binds an individual ZBD_2_ and allows the unbound ZBD_2_s to switch to a proximal state and that this would lead to the enhancement of ClpXP-dependent λO degradation. To test this, we prepared complexes of ClpXP with different combinations of WT ClpX and ClpX∆ZBD protomers whose ZBD_2_ composition per complex can be predicted using combinatorial statistics (see “Methods”). ClpX molecules were obtained by mixing subunits of ClpX and ClpX∆ZBD at different ratios. The initial rates of λO degradation in the absence of TF were obtained (Fig. 3e). We observed an increase in the rate of λO degradation upon increasing ClpX∆ZBD in the complex with maximal degradation rate obtained when the population of complexes consisted of 17% without ZBD_2_, 13% with one ZBD_2_, 13% with two ZBD_2_, and 17% three ZBD_2_ (Fig. 3e, Supplementary Fig. 5e). The degradation rate then decreased upon further increasing the ClpX∆ZBD concentration, and no λO degradation was observed in the absence of ZBD (Fig. 3e, Supplementary Fig. 5e) consistent with the fact that ZBD is needed for λO degradation^25^. These results demonstrate that reducing steric hindrance among the three ZBD_2_ allows for faster translocation of λO through ClpX and into ClpP. Hence, we propose that TF acts by keeping one of the ZBD_2_s in a distal conformation and, as a result, promoting the proximal conformation of the other ZBD_2_s resulting in a faster λO degradation rate (Fig. 3a).

### Direct binding of TF to ClpX

Subsequently, we investigated whether TF directly binds to ClpX, ClpX∆ZBD (AAA+ domain), ZBD, ClpP, and the λO substrate using surface plasmon resonance (SPR) experiments. In these assays, TF was immobilized on the sensor chip and different proteins were injected over the chip (Supplementary Fig. 6). TF was found to interact with ClpX but not ClpP and, within ClpX, it interacted with both the ZBD and AAA+ domains (Supplementary Fig. 6). TF has a higher affinity to the AAA+ domain compared to ZBD (2 µM vs. 12.8 µM, respectively; Supplementary Fig. 6b). It should be noted that, given the oligomeric state of ClpX, the *K*_d_ obtained is an apparent dissociation constant, but it provides a qualitative assessment of the binding between TF and ClpX. TF bound λO with a slightly better affinity than to ClpX (3.8 µM vs. 9.3 µM, respectively; Supplementary Fig. 6b); however, this suggests that λO can partition or be transferred between the two systems.

**Fig. 4:**
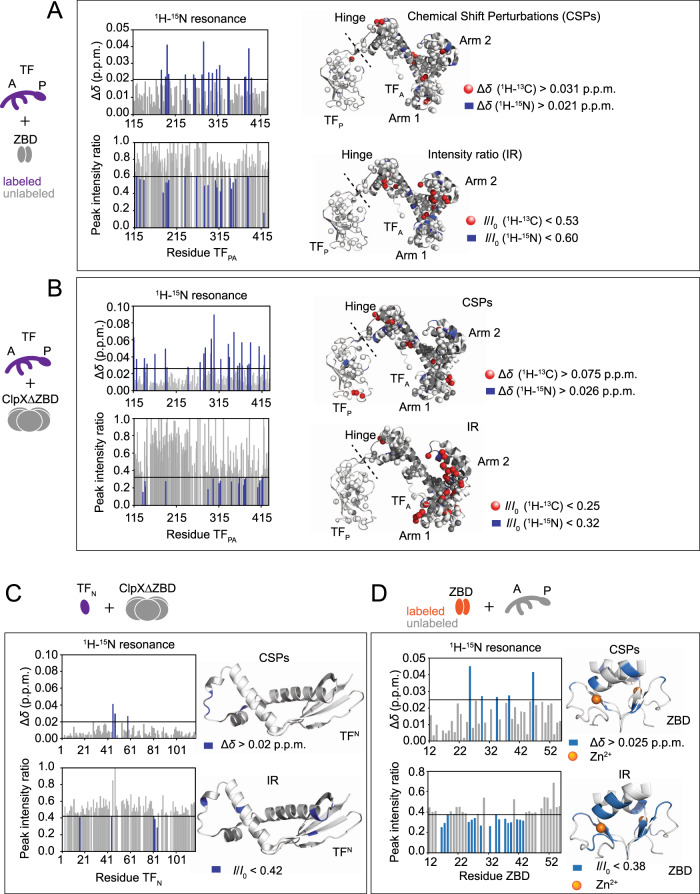
Mapping the interactions between TF and ClpX using NMR. **A**–**D** NMR interaction study for isotopically labeled TF_PA_ and ZBD (**A**) or ClpX∆ZBD (AAA+ domain) (**B**), isotopically labeled TF_N_ and ClpX∆ZBD (**C**), and isotopically labeled ZBD and TF_PA_ (**D**). Upper left and lower left panels represent the graphs for the chemical shift difference and the peak intensity ratio, respectively, of the backbone ^1^H–^15^N resonances. The upper right and lower right panels represent the mapping of the chemical shift perturbations (CPSs) and intensity ratios (IRs) to the crystal structure of TF (PDB: 1W26)^32^ or the solution structure of ClpX ZBD dimer (PDB: 1OVX)^71,72^. The dashed line indicates the domain boundary between TF_A_ and TF_P_. The backbone structure is represented using the ribbon model and the methyl groups of Ile, Leu, Val, Met, and Ala in TF_PA_ are represented as spheres. The methyl groups and backbone of the residues indicating significant changes are colored red and blue, respectively. The zinc ions in ZBD are represented by orange spheres in panel (**D**). The cutoffs of the chemical shift perturbation mapping were determined by ∆*δ*_ave_ + ∆*δ*_SD_, where ∆*δ*_ave_ and ∆*δ*_SD_ are average and standard deviation, respectively, of the chemical shift change upon the addition of the ligand. The cutoff of the intensity change mapping was determined by *IR*_int,ave_ − *IR*_int,SD_, where *IR*_int,ave_ and *IR*_int,SD_ are average and standard deviation, respectively, of the intensity ratio.

To further investigate the multiple interactions between TF and ClpX or λO, nuclear magnetic resonance (NMR) titration experiments were performed. For higher sensitivity and resolution of the NMR spectra, TF was divided into two segments: TF_PA_ (113–432) and TF_N_ (1–117). Although full-length TF exists in a monomer–dimer equilibrium, TF_PA_ and TF_N_ were shown to exist as monomers in solution^31^. Thus, the perturbations are expected to be derived from direct interaction with the titrant and are distinct from the monomer–dimer transition. NMR spectra for isotopically labeled TF_PA_ and TF_N_ in the absence and presence of λO, ZBD, or ClpX∆ZBD (AAA+ domain) were acquired and the chemical shift perturbation (CSP) and intensity reductions (IR) were monitored (Fig. 4a–c, Supplementary Figs. 7–11). For TF_PA_, the resonances from backbone amide groups as well as methyl groups were monitored, whereas only those from backbone amide groups were monitored for TF_N_.

First, the interaction between TF and λO was investigated. The addition of λO induced significant CSP and IR to the resonances of TF_PA_ (Supplementary Fig. 7). On the other hand, much less significant perturbations were observed for the resonances of TF_N_ upon the addition of λO (Supplementary Fig. 8), indicating that λO is mainly recognized by P and A domains of TF. Mapping of the perturbations onto the structure of TF shows that the affected amino acid residues are located at multiple sites, especially at the P domain and Arm 1 of the A domain (Supplementary Fig. 7b, c), which are known to be involved in the recognition of client proteins^31^.

Next, we investigated the interaction between TF and ZBD or ClpX∆ZBD (Fig. 4a–c). The addition of ZBD induced significant perturbations to several resonances of TF_PA_ (Fig. 4a, Supplementary Fig. 9). The mapping of the perturbed resonances on the structure of TF indicated that multiple regions of TF, including arm 2 and the hinge region connecting the TF_P_ and TF_A_ domains are involved in the recognition of ZBD (Fig. 4a). On the other hand, the resonances from TF_N_ indicated very small perturbations (Supplementary Fig. 10). Thus, the NMR data indicate that TF_A_ is responsible for the recognition of ZBD in ClpX.

The addition of ClpX∆ZBD induced significant perturbations to the resonances from TF_PA_ (Fig. 4b, Supplementary Fig. 11a, b). More specifically, significant chemical shift changes and/or peak IR were observed for the resonances derived from the tip of the TF_P_, the hinge, arm 1, and arm 2, indicating that these regions of TF are responsible for the recognition of ClpX∆ZBD. On the other hand, the addition of ClpX∆ZBD to TF_N_ induced minor chemical shift changes for the resonances located around the loop region, indicating that the loop region of TF_N_ may weakly interact with ClpX∆ZBD (Fig. 4c, Supplementary Fig. 11c).

The NMR data show that the perturbed resonances are derived from multiple regions of TF. Although some of these perturbations can be derived from allosteric effects and not always indicate direct contacts, the data suggests that multiple regions of TF are involved in the binding of ZBD and AAA+ domains of ClpX. It is important to note that more regions of TF are perturbed by the addition of ClpX∆ZBD compared to ZBD, suggesting that the greater affinity of TF for the AAA+ domain obtained by SPR (Supplementary Fig. 6) can be attributed to more residues involved in the interaction. Notably, several hydrophobic amino acid residues of TF, which were identified to be involved in the interaction with ClpX, are also responsible for the recognition of the client protein λO (Supplementary Fig. 7), as well as, other unfolded proteins as shown in the solution structure of TF in complex with PhoA^31^ (Supplementary Fig. 12a, b). The overlap between ClpX-binding sites and the substrate-binding sites on TF suggests competition between the substrate protein and ClpX for binding to TF. Furthermore, some of the ClpX-binding sites on TF overlap with the interface of the TF dimer^8^ (Supplementary Fig. 12c), which suggests that TF binds to ClpX as a monomer.

To identify the TF-binding site on ZBD dimer, we measured the NMR for isotopically labeled ZBD in the absence and presence of TF_PA_ (Fig. 4d, Supplementary Fig. 11d). Our data show that the addition of TF_PA_ induced significant intensity reduction for the resonances originating from the residues located on the hydrophobic surface of ZBD (Supplementary Fig. 12d). This observation highlights yet again, the importance of the hydrophobic interaction between TF and ClpX.

### Peptide array analysis indicates binding between TF and the AAA**+** domain of ClpX

**Fig. 5:**
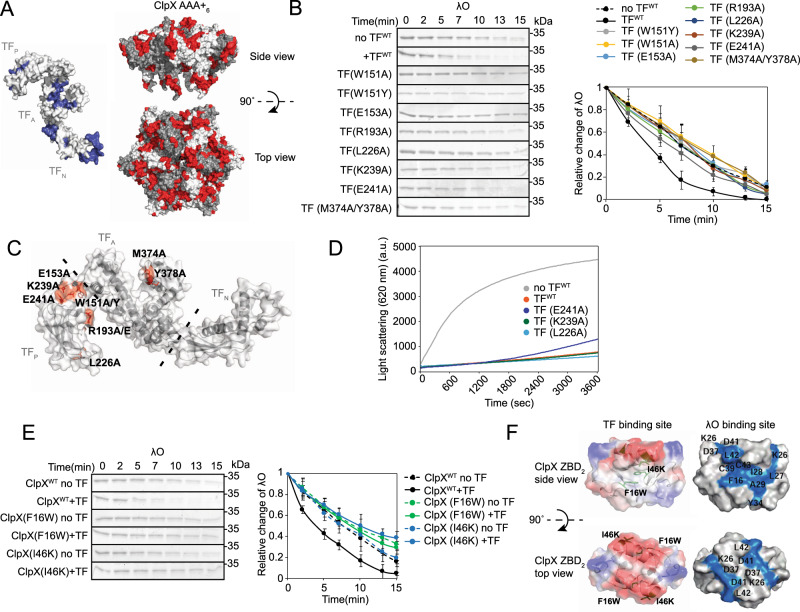
Peptide array and mutagenesis analyses confirm binding sites between TF and ClpX. **A** Surface representation of the 3D structure of TF (PDB:1W26)^32^ and the ClpX∆ZBD hexamer (PDB:6PP5)^33^ highlighting binding motifs between ClpX and TF. For the TF structure, regions that bound overlapping ClpX peptides (motifs 1–6) are shown in blue, and regions that bound overlapping TF peptides (motif 1–12) on ClpX∆ZBD (AAA+) are in red. ClpX∆ZBD protomers are shown in alternating shades of gray. **B** Densitometry of in vitro degradation assays using the indicated TF mutants that abolish the enhancement effect by TF on λO degradation by ClpXP. Proteins were analyzed by SDS-PAGE and band intensities were quantified and plotted as shown. Representative gel images are shown. Data are presented as mean values and error bars indicate the standard deviations of at least three repeats. **C** Location of residues from (**B**) on the TF protein structure with the side chains highlighted in red. TF domains are labeled, and the dashed lines indicate the protein domain boundaries. **D** Aggregation of GAPDH in the absence and presence of the indicated TF wild type and mutants using light scattering measurements. **E** Same as in (**B**) but using ClpX with mutations in the ZBD domain. Data are presented as mean values and error bars indicate the standard deviations of at least three repeats. **F** Combined ribbon and surface representation of the ZBD dimer. The location of the residues from (**E**) is shown with their side chains in green. The structures also show the electrostatic surface potential of ZBD with negatively charged, positively charged, and hydrophobic residues in red, blue, and gray, respectively. The bottom panel is rotated 90° along the horizontal axis with respect to the top panel. The ZBD dimer structure on the right shows the λO-binding site in blue color^35^. Source data are provided as a Source Data file.

Given the difficulty in obtaining and assigning the NMR spectra of ClpX∆ZBD, we further characterized the interacting motifs between ClpX AAA+ and TF using peptide array technology. Miniaturized libraries of overlapping 12mer peptides were synthesized by walking through the amino acid sequence of ClpX∆ZBD or TF, advancing two (ClpX∆ZBD) or three (TF) amino acids at a time (Supplementary Fig. 13). The TF peptide array was first probed with His-ClpX∆ZBD. Binding of His-tagged protein was detected using 6×His mAB-HRP. Peptides that were recognized directly by the antibody probe were eliminated from the analysis. TF peptides recognized by His-ClpX∆ZBD and containing at least two consecutive spots on the membrane were grouped, resulting in six consensus motifs (motifs 1–6) (Supplementary Fig. 13a, c). The sequence of the four isolated peptides, which did not have neighboring spots on the membrane, are also shown (Supplementary Fig. 13a). Consensus motifs and binding peptides were mapped onto the crystal structure of TF^32^ for visualization (Fig. 5a). The ClpX∆ZBD peptide array was probed in a similar manner using purified His-TF resulting in 12 consensus motifs (motifs 1–12) in ClpX∆ZBD (Supplementary Fig. 13B, D), which were mapped onto the cryoEM structure of ClpX∆ZBD^33^ (Fig. 5a). Together, our peptide array analyses are consistent with the NMR results (Fig. 4) and support a model whereby TF_P_ and TF_N_ predominantly bind the AAA+ domain of ClpX, and TF_A_ mainly binds the ZBD.

### Mutagenesis confirms the binding sites between TF and ZBD of ClpX

To better understand the mechanism by which TF enhances λO degradation by ClpXP, we performed a mutagenesis screen to identify sites within TF that abolish this effect. A total of 38 individual or combined mutations of TF were generated mainly by replacing the target residue with mostly Ala (Supplementary Fig. 14a), and each mutant was tested in degradation assays. Eight TF mutants were found to significantly prevent the enhancement of ClpXP-dependent degradation of λO (Fig. 5b, Supplementary Fig. 14b). Consistent with our previous results from NMR and peptide array analysis, the location of these mutations are found at the tip of the P-domain, the hydrophobic pocket of arm 2, and the residues surrounding the hinge region connecting the P- and the A-domains (Fig. 5c). Interestingly, the TF(F198A) mutation known to abolish peptidyl-prolyl *cis/trans* isomerase (PPIase) activity^34^ behaved like wild-type TF suggesting that the PPIase function of TF is not required for enhancement of ClpXP activity (Supplementary Fig. 14b). This is consistent with our previous results (Supplementary Fig. 5d).

To test whether these residues affect chaperone function of TF, we performed anti-aggregation assays using GAPDH as a substrate. GAPDH denatured in guanidine was diluted into a solution lacking guanidine and the formation of GAPDH aggregates was monitored by light scattering. In the absence of TF, GAPDH forms aggregates as indicated by the rapid increase in light scattering, while the presence of TF significantly suppressed the formation of aggregates demonstrating the anti-aggregation activity of TF (Fig. 5d). No effect on anti-aggregation activity was observed for TF mutants K239A and L226A, while TF mutant E241A had a small change in anti-aggregation activity (Fig. 5d). Taken together, these results suggest that these three residues are important in the functional interaction between TF and ClpX (Fig. 5b, c), rather than the interaction between TF and its substrate.

Our previous results indicated that ZBD of ClpX is a critical binding site for TF (Figs. 1e, 3, and 4a, d); hence, we performed degradation assays of λΟ using ClpX variants with mutations in ZBD in the absence and presence of wild type TF. Out of the ten residues mutated, Phe16 and Ile46 located within the hydrophobic side pocket of the ZBD dimer were found to completely abolish the TF-enhancement of λO degradation by ClpXP (Fig. 5e, f, Supplementary Fig. 14d). This is also consistent with our NMR results (Fig. 4d). Importantly, these mutations did not affect ClpX activity as ClpX was still able to recognize and unfold λO for degradation by ClpP (Fig. 5e) in agreement with our previous work using these mutants^35^.

This observation highlights the importance of the hydrophobic interactions between TF and ClpX. Furthermore, the hydrophobic region encompassing F16 and I46 overlaps with the binding site of the λO substrate^35^ (Fig. 5f), suggesting that: (1) TF and λO might alternate in binding to the same ZBD_2_ or (2) TF and λO bind simultaneously to ClpX but use different ZBD_2_s within the hexameric complex.

### TF and ClpX adaptors use a similar mechanism to enhance λO degradation by ClpXP

Based on our previous data, we hypothesize that TF restricts the conformation of some but not all ZBD dimers upon binding and this, in turn, allows access of other ZBD_2_s into the pore of ClpXP (Fig. 3a). Some adaptor proteins, like SspB and RssB, have also been shown to bind the ZBD of ClpX and to regulate the ClpXP-mediated degradation of their cognate substrates^36–38^. Indeed, they bind to the same hydrophobic region bound by TF (Fig. 5f).

We tested the degradation of λO by ClpXP in the absence and presence of equimolar concentrations of SspB, RssB, SspB with TF, and RssB with TF. As shown in Supplementary Fig. 15, the presence of SspB or RssB alone also enhanced the degradation of λO. To show that the enhancement of λO degradation by ClpXP requires the binding of SspB to ZBD, we used the truncation mutant SspB127 lacking the ClpX-binding sequence^37^ and observed that the enhancement effect was abolished (Supplementary Fig. 15b). In addition, we find that, like TF, SspB, and RssB are unable to enhance the degradation of another ZBD-binding substrate such as MuA (Supplementary Fig. 15c). These results show that ZBD is a common docking site for multiple adaptors including TF and that binding to ZBD is enough to enhance λO degradation.

### TF facilitates the degradation of newly synthesized proteins in *E. coli*

**Fig. 6:**
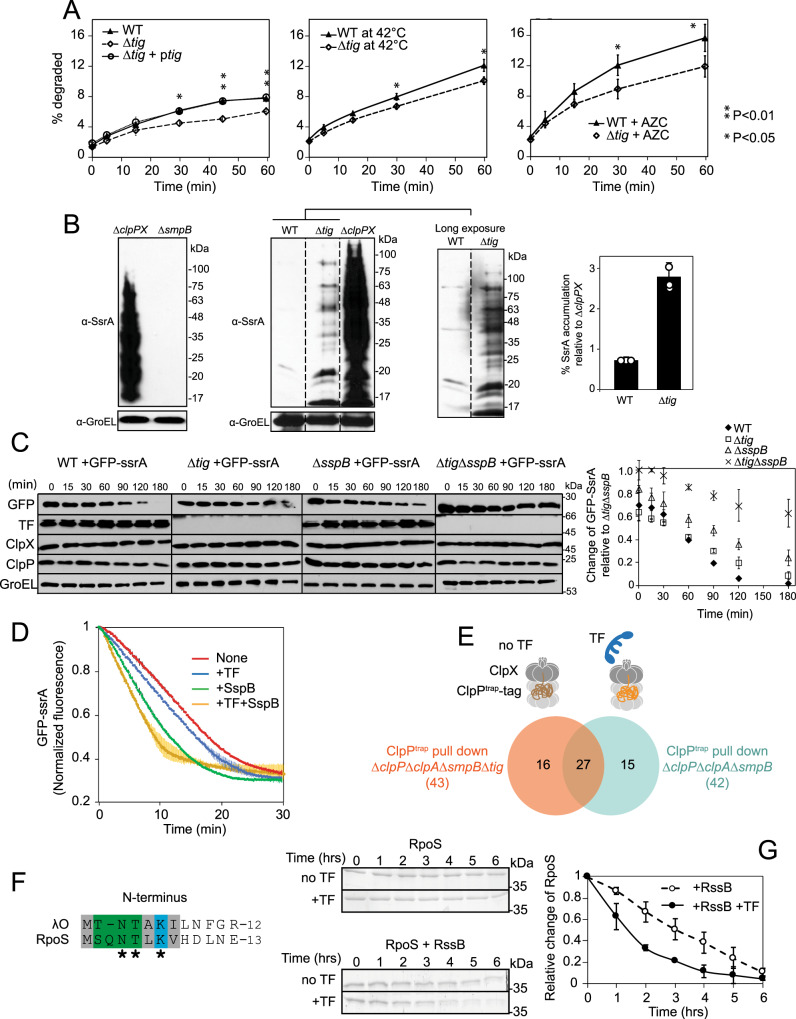
TF affects the ClpXP-mediated degradation of other substrates in the cell. **A** Pulse-chase experiments were carried out using [^35^S] methionine to assess the degradation of newly synthesized proteins in different strain backgrounds at mid-log phase at 37 °C (left panel), after heat shock at 42 °C for 10 min (middle panel), or after growth in the presence of 100 µg/mL AZC for 30 min at 37 °C (right panel). Statistical tests: *P* values determined by two-sided unpaired *t* test. Error bars represent the standard deviations from *n* = 3 independent replicates. **B** Western blot of SsrA-tagged proteins in MC4100 WT, ∆*clpPX*, ∆*tig*, and ∆*smpB* strains using an anti-SsrA antibody. The immunoblot on the left has less exposure than the ones on the right. The bar plot on the right shows the mean quantification values of SsrA-tagged protein accumulation in the WT and ∆*tig* strain relative to ∆*clpPX*. Error bars show standard deviations of three replicates. **C** Western blot analysis of the degradation of overexpressed GFP-ssrA as a function of time after inhibition of protein synthesis in MC4100 WT, ∆*tig*, ∆*sspB*, and ∆*tig*∆*sspB* cells. About 10^5^ cells were loaded for each time point. Curves on the right show the quantification of the bands. The error bars are the standard deviation based on three independent experimental repeats. SspB deletion strains were confirmed by PCR. **D** Degradation of GFP-ssrA by ClpXP in the presence and absence of TF, SspB, and TF+SspB measured by fluorescence (*λ*_em_ = 395 nm/*λ*_ex_ = 509 nm). Curves represent the average of triplicate experiments and the error bars the standard deviations. **E** *E. coli* MC4100 cultures of strains Δ*clpP*Δ*clpA*Δs*mpB*Δ*tig* (no TF strain) and Δc*lpP*Δ*clpA*Δ*smpB* (TF strain) expressing the ClpP^trap^ were grown to mid-log phase followed by pull-down of the ClpP^trap^ allowing the identification of trapped proteins by LC–MS/MS. The numbers of identified proteins are represented in a Venn diagram. **F** Sequence alignment of the N-termini of λO and RpoS. **G** In vitro degradation assays of RpoS by ClpXP in the presence or absence of RssB or TF. The curves shown on the right are derived from three independent reactions and the error bars are the standard deviations for each time point. Source data are provided as a Source Data file.

To determine if TF has a global effect on the degradation of newly synthesized proteins in *E. coli*, we performed pulse-chase experiments in WT and Δ*tig* cells under normal growth conditions (37 °C in LB media). The overall proteolysis of newly synthesized proteins was slower in the Δ*tig* strain compared to WT (Fig. 6a). One hour after the chase, about 7.7% and 6.0% of newly synthesized proteins were degraded in WT and Δ*tig* cells, respectively. The lower degradation capacity in Δ*tig* strain was complemented by placing the *tig* gene under its own promoter on a plasmid back into Δ*tig* cells (Δ*tig* + p*tig*, Fig. 6a left panel). Evidently, TF is responsible for facilitating the degradation (rather than folding) of about 2% of newly synthesized proteins under regular growth conditions.

Under heat shock conditions (42 °C), there was an overall increase in the total amount of proteins degraded compared to 37 °C but the difference between WT and Δ*tig* at 42 °C was similar to that at 37 °C (12.0% in WT and 10.0% in Δ*tig* cells after 1 hr, Fig. 6a middle panel). This could possibly be related to the fact that TF is cold shock-induced rather than heat shock induced^39^. When protein misfolding was induced in cells by adding the proline analogue azetidine-2-carboxylate (AZC), the overall proteolysis of newly synthesized proteins after 1 h of the chase was 15.6% in WT and 11.8% in Δ*tig* cells (Fig. 6a right panel), higher than that for normal growth conditions. Hence, under this condition, TF promoted the degradation of twice the number of newly synthesized proteins (about 4%) compared to the regular growth condition.

### Degradation of SsrA-tagged proteins is affected in *tig* deletion mutant strain

To test if TF has a global effect on the ClpXP-mediated degradation of SsrA-tagged proteins, we grew WT, ∆*tig*, ∆*clpPX*, and ∆*smpB* cells at 37 °C to mid-log phase (OD600 ~0.6); cells were then harvested, and total proteins were isolated using trichloroacetic acid (TCA) precipitation. The *smpB* gene encodes for the RNA chaperone essential for SsrA-tagging on stalled ribosomes^40^. Subsequently, western blot analysis was performed of equally loaded samples using an anti-SsrA antibody. As expected, the ∆*smpB* strain showed no accumulation of SsrA-tagged proteins. In contrast, ∆*clpPX* strain exhibited a significant accumulation of such proteins highlighting the fact that SsrA-tagged proteins are predominantly degraded by ClpXP under our experimental conditions (Fig. 6b). Importantly, the ∆*tig* strain exhibited about threefold higher accumulation of SsrA-tagged proteins compared to WT (Fig. 6b).

Since the ClpXP-dependent degradation of GFP-SsrA is also modulated by TF (Fig. 1b), we tested if that holds true in vivo. Overexpressed GFP-ssrA was degraded at a faster rate in WT compared to ∆*tig* strain (Fig. 6c). It is known that the SspB adaptor can enhance GFP-SsrA degradation by about tenfold^41–43^. In vivo, we found that the absence of *sspB* slowed GFP-ssrA degradation more than in the strain lacking *tig* (Fig. 6c). However, the double mutant (∆*tig*∆*sspB*) significantly accumulated more GFP-ssrA. These results strongly suggest that TF has a similar role as SspB for the degradation of SsrA-tagged proteins.

We confirmed the effect of TF on GFP-ssrA by performing degradation assays measuring fluorescence and, for comparison, in the presence of SspB (Fig. 6d). We noticed that, when TF and SspB were added together, the rate further increased suggesting they have an additive effect on ClpX in enhancing GFP-ssrA degradation. Taken together, our data suggest two possible scenarios: (1) TF may work together with the SspB adaptor in the delivery or processing of SsrA-tagged proteins by ClpXP, or (2) TF might solely be responsible for the ClpXP-dependent degradation of a small subset of SsrA-tagged proteins. In this regard, it is important to note that SspB activity was initially identified in partially purified ribosomes^44^.

The above results imply that the lack of both TF and SspB could lead to the accumulation of incomplete polypeptides in the cell, which should cause fitness defects. To test this, we checked the growth of MC4100 ∆*tig*, ∆*sspB*, and ∆*tig*∆*sspB* strains at 30 °C. There were no major differences in growth between the single mutants ∆*tig* and ∆*sspB* compared to WT cells (Supplementary Fig. 16a), suggesting that TF and SspB are not essential for cell growth at these temperatures. In contrast, the double mutant ∆*tig*∆*sspB* showed growth impairment that was restored by overexpressing *tig* under the control of its native promoter from a plasmid (Supplementary Fig. 16a). These results suggest that *tig* and *sspB* have a negative genetic interaction and, hence, likely function in related pathways.

### The ClpXP-dependent degradation of RpoS is also modulated by TF

To discover other ClpXP substrate proteins whose degradation is modulated by the TF chaperone, we used a catalytically inactive ClpP protease that would act as a trap (ClpP^trap^) for substrates that are translocated into the protease chamber. The ClpP^trap^ was C-terminally tagged by a SPA-tag, which can be used for pulldown experiments^45^, and was expressed in *E. coli* MC4100 cells lacking the endogenous *clpP* as well as the *clpA* genes since ClpA can also act as a cap for ClpP. The *smpB* gene was deleted to prevent the formation of SsrA-tagged proteins that would also be trapped. Proteins that co-purified with ClpP^trap^-SPA in Δ*clpP*Δ*clpA*Δ*smpB* strain were compared to those identified in the Δ*clpP*Δ*clpA*Δ*smpB*Δ*tig* strain using mass spectrometry approaches. Control experiments were carried out using the WT ClpP-SPA construct.

Identified proteins from each background were filtered at a peptide count greater or equal to three and of high confidence (>95%) (Supplementary Fig. 16b). In total, 43 proteins were identified from the strain lacking TF and 42 from the strain expressing TF (Fig. 6e). From these, 15 were trapped only in the presence of TF including RpoS (also known as *σ*^S^; Fig. 6e, Supplementary Fig. 16b), the master regulator of stationary phase^46^. RpoS is a well-established ClpXP substrate^47^.

Interestingly, the N-terminus of RpoS shares sequence similarity with the N-terminus of λO (Fig. 6f). The degradation of RpoS by ClpXP was tested in vitro and, consistent with previous findings, the RpoS protein was not degraded or only slowly degraded in the absence of its ClpX-specific adaptor protein RssB^48^. We note that the degradation was not significantly influenced by the TF chaperone (Fig. 6g). When RssB and TF were present together, the RpoS protein was degraded ~1.5-fold faster by ClpXP (Fig. 6g). Indeed, phosphorylated RssB is known to interact and modify the structure of RpoS likely by exposing a sequence in the protein that is quickly recognized by ClpX leading to RpoS degradation by ClpXP^49,50^. Hence, TF can modulate ClpXP-mediated degradation of RpoS only after its recognition tag is exposed by RssB, suggesting that TF can enhance RpoS degradation after it binds to RssB.

### Degradation of ribosome-stalled λO is enhanced by TF

**Fig. 7:**
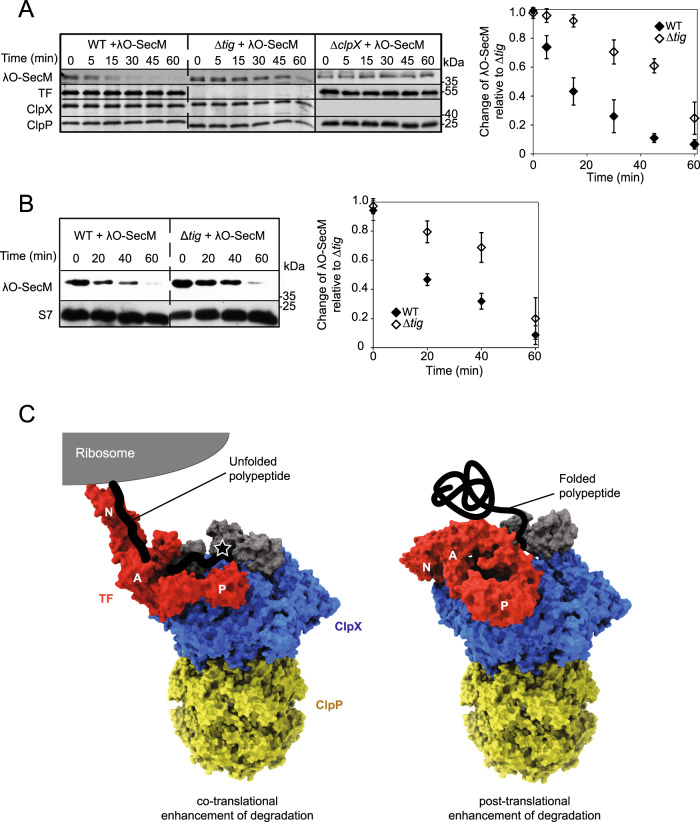
TF enhances the ClpXP-dependent degradation of λO in a cotranslational manner. **A** Western blot analysis showing λO-SecM degradation. λO-SecM was induced from a plasmid in different strain backgrounds and then translation was inhibited. Quantification of the λO-SecM band as a function of time is shown on the right based on three experimental repeats. Error bars show the standard deviation for each time point. **B** Same experiment as in (**A**) but shown is the amount of λO-SecM in the ribosomal fraction obtained after separation of cell lysates on sucrose cushions. Samples with equal OD_260_ were loaded to ensure that each point contained equal amount of ribosomal material as indicated by the western blot for S7 of the small ribosomal subunit. Quantification of the λO-SecM band as a function of time is shown on the right based on three experimental repeats. Error bars show the standard deviation for each time point. **C** Cartoon model of the role of TF in the ClpXP-dependent degradation of newly synthesized proteins in a co-translational and post-translational context (see text for details). The star represents the ClpX recognition sequence in the substrate. Source data are provided as a Source Data file.

We showed above (Figs. 2a and 6a) that TF promotes the degradation of newly translated proteins. This could occur either cotranslationally or shortly after release from the ribosome. To further establish that TF stimulation of ClpXP-dependent degradation of λO can occur cotranslationally we performed the following experiment. The C-terminus of λO was fused to the SecM motif composed of 17 amino acids, which has been found to interact with the ribosomal polypeptide exit tunnel and to cause translational arrest^51^. We verified that λO-SecM is indeed arrested on ribosomes by fractionation on sucrose cushion. The degradation of the fusion protein was visualized by western blot analysis after inhibiting translation by tetracycline. Unlike the case for λO (Fig. 2a–c), the levels of λO-SecM were similar in WT, Δ*tig*, and Δ*clpX* cells at the zero time point (Fig. 7a). However, like λO, the degradation of λO-SecM was slower in the absence of TF compared to WT (Fig. 7a).

To ensure that the degradation of λO-SecM occurred while the protein was attached to the ribosome, cell lysates at different time points of the experiment were separated on sucrose cushions (see “Methods”) and the ribosomal fractions were blotted for λO-SecM. Ribosome bound λO-SecM was degraded at a slower rate in Δ*tig* compared to WT strain (Fig. 7b). Overall, these results indicate that the ClpXP-dependent degradation of λO-SecM is predominantly cotranslational and is facilitated by TF.

### TF complements for the lack of endogenous ClpX-adaptor function in other bacterial species

We noticed that the *sspB* gene is present in most Proteobacteria but absent in the rest of the bacterial kingdom (Supplementary Figs. 1 and 17a). In contrast, the *tig* gene is conserved. For example, the Gram-positive dental pathogen *Streptococcus mutans* does not contain *sspB* or SspB-like adaptors of ClpX but has *tig, clpX, clpP*, and *smpB* genes. Therefore, to demonstrate that TF could act as an ancestral adaptor of ClpX, we tested for any TF-dependent effects on the degradation of GFP-ssrA in *S. mutans* by performing in vitro degradation assays. The *S. mutans* SsrA tag contains a valine residue (VAA) instead of leucine in the last three residues of the ClpX recognition motif (Supplementary Fig. 17b); so, we used GFP with the *S. mutans* SsrA-tag fused to it at the C-terminus as a substrate. Our experiments show that in the absence of TF^sm^, degradation of 5 µM GFP-ssrA^sm^ was slow (2 µM ClpX^sm^ and 2.4 µM ClpP^sm^), but surprisingly when TF^sm^ (15 µM) was added to the reaction, GFP-ssrA^sm^ was degraded >2x faster (Supplementary Fig. 17b). GFP-ssrA containing the *E. coli* sequence was not recognized by ClpXP^sm^. This suggests that TF has an ancestral SspB-like function in *S. mutans* capable of modulating the ClpXP-mediated degradation of SsrA-tagged proteins.

## Discussion

In this work, we have demonstrated a functional interaction between TF and ClpXP. The presence of such a functional interaction was initially suggested by the close proximity of the *tig* gene to the *clpX* and *clpP* genes as well as *lon* across bacteria. Using purified proteins, we found that, while different chaperones had no effect or slowed down the degradation of ClpXP substrates, TF was the only chaperone capable of enhancing the degradation of λO, GFP-ssrA, and RpoS substrates. Indeed, we estimate that about 2% of newly synthesized proteins are degraded in a TF-dependent manner. Based on a kinetic analysis, TF acts to enhance the enzymatic selectivity of ClpXP for certain substrates such as λO and to increase the catalytic efficiency of the protease almost threefold. TF also enhances the ATPase activity of ClpX.

The presence of an exposed degron sequence on the substrate protein is important as well. The results of the in vitro and in vivo experiments of Fig. 2 demonstrate that the N-terminus of λO is required for the enhancement of its ClpXP-dependent degradation by TF. Fusing the N-terminus of λO to other proteins such as S7 also allowed for such enhancement to be observed. This requirement is essential for selective TF-mediated enhancement of ClpXP degradation as TF is highly abundant in the cell.

Interestingly, the experiments of Fig. 3 indicate that TF interacts with a ZBD dimer, thus allowing the other ZBD_2_s, which perhaps are bound to a substrate, to move freely towards the ClpP chamber. In doing so, we proposed that TF enhances the degradation of these substrates. Thus, the binding of TF to the ZBD primes ClpXP to degrade ZBD-bound substrates. Indeed, a similar effect is seen with the *Caulobacter crescentus* adaptor CpdR, where its binding to the ZBD primes ClpX to recruit downstream components^52^. Our results highlight the critical role of ZBD in the enhancement effect TF has on substrate degradation by ClpXP. ZBD is a well-known substrate and adaptor protein recognition hotspot on ClpX, and, hence, this further suggests that TF functions as an adaptor protein of ClpX.

To gain an understanding of the molecular basis by which TF does this, we mapped the binding interfaces between TF and ClpX using SPR, NMR, peptide array analysis, and mutational analysis. These data show that the binding interface between TF and ClpX involves all three domains of TF (P, A, and N) and both domains of ClpX (ZBD and AAA+). The TF_P_ and the TF_N_ domains interact mainly with the AAA+ domain of ClpX and more specifically to regions located between the large and small subunits of the hexameric ring. The TF_A_ domain interacts with the ZBD of ClpX in a region that partially overlaps with the λO binding site on ZBD. Notably, several hydrophobic amino acid residues of TF, which were identified to be involved in the interaction with ClpX, are also responsible for recognizing λO and other unfolded protein substrates as shown in the solution structure of TF in complex with PhoA^31^. Our data show that some of the single point mutations of TF have no effect on substrate recognition, but induce significant reduction in the interaction with ClpX, which suggests a specific interaction between TF and ClpX. Overlap between ClpX-binding sites and the substrate-binding sites on TF implies competition between the substrate protein and ClpX for binding to TF. A similar mechanism was observed recently between the RssB adaptor, ClpX and its cognate substrate RpoS^38^. Furthermore, some of the ClpX-binding sites on TF overlap with the interface of the TF dimer^8^, suggesting that TF binds to ClpX as a monomer.

TF also enhanced the ClpXP-dependent degradation of SsrA-tagged proteins both in vitro and in vivo. This observation leads us to propose that ribosome-bound TF is also important for mediating the degradation of SsrA-tagged proteins soon after their release from the ribosome. This might be a distinguishing feature of TF compared to SspB, as SspB might act to promote the degradation of proteins at later stages after their release from ribosomes. In this regard, it was very striking to find that the impaired growth of cells lacking both TF and SspB can be restored upon overexpression of TF, implying an overlap in the function of these two proteins.

Interestingly, the N-terminus of RpoS is similar to that of λO. However, the effect of TF on RpoS degradation required the presence of the RssB adaptor. We speculate that RssB exposes the N-terminus of RpoS to allow for its degradation by ClpXP. This leads us to propose that the ClpXP-dependent degradation of proteins having an unstructured N-terminal sequence similar to that of λO can be modulated by TF.

Indeed, as shown in Supplementary Fig. 17, bacterial species lacking SspB likely rely on TF to promote the ClpXP-dependent degradation of their SsrA-tagged proteins. Hence, while TF might act as a chaperone to fold proteins in some instances, it can also act as a chaperone to promote protein degradation rendering TF a central hub in modulating the flux of proteins between folding and degradation.

Based on our work, we propose the presence of both a (1) co-translational and a (2) post-translational model of protein degradation in prokaryotes (Fig. 7c). In the first scenario, TF acts as a bridge between the ribosome and ClpX. It is possible that TF_N_ binds the ribosome, TF_P_ binds the AAA+ domain of ClpX and TF_A_ binds ZBD. A nascent polypeptide chain exiting the ribosomal exit tunnel would first interact with TF_A_ and then be transferred to the ZBD or AAA+ domain of ClpX via their respective recognition sequences (Fig. 7c, left panel). The transfer of the nascent chain from TF to ClpX would also lead to the enhancement of ClpX ATPase activity. This model assumes a static interaction of TF with the ribosome. Alternatively, since TF interaction with the ribosome is dynamic^53^, the second scenario would involve the formation of a complex of TF-ClpXP that is capable of binding ZBD-bound substrates. Here, TF_N_ and TF_p_ anchor to the AAA+ domain of ClpX and the TF_A_ partially binds to the ZBD. It is important to note that for each ClpX ring, there are six AAA+ domains and three ZBD_2_ that have potential binding sites for TF and for substrates. TF-bound to a ZBD_2_ would then allow another ZBD_2_ bound to a substrate to move towards the ClpXP pore (Fig. 7c; right panel).

The association between a highly promiscuous and abundant chaperone-like TF with a main protease like ClpXP implies a system in which many diverse proteins are sampled promptly for folding or degradation in the cell. The role of TF as an adaptor protein of ClpXP is not only a new function of the chaperone but also describes a novel mechanism of proteostasis in bacteria.

## Methods

### Phylogenetic analysis

The nucleotide sequences of *tig*, *smpB*, *sspB*, *rssB*, *clpP*, *clpX*, and *lon* were retrieved from the NCBI nonredundant protein database using the blastx function of BLAST v. 2.6.0+^54^. The resulting sequences were aligned, and the generated multiple sequence alignments were employed to create the phylogenetic tree using the CLC Genomics Workbench v. 11.0.1 (Aarhus, Denmark). The phylogenies were calculated using the Neighbor-Joining method with BLOSSUM62 distance matrix^55^, and the confidence of the topologies were estimated using a bootstrap approach with 500 iterations.

### Bacterial strains

Gene deletions were performed with the P1 phage transduction method using the *E. coli* Keio library^56^. Clean knockouts were obtained by using the temperature-sensitive pCP20 plasmid encoding the FLP recombinase to remove the resistance cassette. The generated strains had no resistance markers and were verified by western blotting using α-TF (1:5000 dilution), α-ClpX (1:5000), α-ClpP (1:3000), and α-SsrA (1:3000) antibodies (Supplementary Fig. 18). ClpA and SspB KO were confirmed by PCR. Refer to Supplementary Table 1 for strains used in this study.

### Plasmids

The gene for the catalytically inactive ClpP mutant S111A was fused to a SPA affinity purification tag at its 3′ end and cloned into a modified pPAL7 (Bio-Rad) vector to generate a ClpP^trap^-SPA construct. As a control, the same construct was made using WT ClpP. S7 was cloned into p11 vector^57^ with an N-terminal His-tag followed by a TEV protease cleavage site for purification of S7 protein. To monitor S7 degradation in vivo, untagged S7 was cloned into pET3a vector under the control of the T7 promoter and transformed into WT, *tig::cat*, and *clpX::cat* MC4100 strains. To express S7 in MC4100 strains, the pT7pol26 plasmid expressing T7 RNA polymerase^58^ was also transformed into these strains. The fusion construct N_λO_-S7 was made by fusing the N_λO_: MTNTAKILNFGR encoding sequence to the N-terminus of S7. the pBAT-His-Sumo-Barnase-SecM plasmid was a gift from Dr. Bernd Bukau (University of Heidelberg, Germany). Barnase was removed and λO was inserted between the AflII and BamH1 sites of pBAT plasmid to generate pBAT-His-Sumo-λO-SecM. To generate a construct without His-Sumo, the sequence of λO-SecM was PCR amplified from pBAT-His-Sumo-λO-SecM and inserted into pET15b plasmid between the NcoI and HindIII cut sites. For endogenous λO protein turnover experiments, WT, *tig::cat*, and *clpX::cat* MC4100 strains were lysogenized with λ*cI*857Sam7 phage, which was provided by Dr. Alan Davidson (University of Toronto, Canada). *Streptococcus mutans* UA159 was used to obtain TF^*S. mutans*^, ClpP^*S. mutans*^ and ClpX^*S. mutans*^, that were inserted into pProEXHTb. The SsrA^*S. mutans*^ sequence -AKNTNSYAVAA was used to make GFP-ssrA^*S. mutans*^.

Plasmid pPK823 (pET11a) overexpressing Fnr was from Dr. Patricia K. Kiley (University of Wisconsin, USA). Plasmid pUHE21‐2 expressing the FlhDC complex was from Dr. Tomoko Yamamoto (Chiba University, Japan)^59^. Plasmid pHF010 expressing λN-His protein was from Dr. Irene Lee (Case Western Reserve University, USA). RssB was cloned into a pET-SUMO vector with a N-terminal SUMO-tag followed by a His-tag and a SUMO protease cleavage site for purification of RssB protein. Mutagenesis was carried out using the QuickChange kit (Stratagene).

Primers used for cloning several of the plasmids and mutants are given in Supplementary Table 2.

### Protein purification

All recombinant proteins were overexpressed in *E. coli* BL21(DE3) cells. The plasmid pProEXHTa TF expressing trigger factor was from Dr. José M. Barral (University of Texas, USA). TF was induced at mid-log phase with 1.0 mM IPTG for 3.5 h at 37 °C. His-tagged TF was purified on Ni-NTA column (Qiagen) and washed with 50 mM imidazole in buffer A (25 mM Tris-HCl, pH 7.5, 300 mM NaCl, 10% glycerol, and 1 mM DTT) to remove ATPase contaminants before elution. Eluted TF fractions were tested and only fractions without ATPase activity were collected. The His-tag was removed using His-tagged TEV protease during dialysis in buffer B (25 mM Tris-HCl, pH 7.5, 200 mM KCl, 10% glycerol, and 1 mM DTT). Purification of TF was performed in one day and the protein was flash-frozen in liquid nitrogen after removing TEV. Purification of the ClpX protein using the plasmid pProEXHTb ClpX was performed the same way as TF. Purification of RssB expressed from the pET-SUMO plasmid was carried out as for TF but using SUMO protease to remove the SUMO-His tag.

ClpP^60^, ClpA^61^, λO^62^, λN^63^, MuA^64^, S7^65^, Fnr^66^, RpoS^23^, and SspB^25^ were expressed and purified according to published protocols. HtpG was precipitated in 70% ammonium sulfate and further purified using an anion exchange Q Sepharose column followed by size exclusion chromatography in 50 mM NaH_2_PO_4_, 300 mM NaCl, 10% glycerol. FlhDC complex proteins were purified as described^59^.

### Peptide array synthesis and analysis

Miniaturized peptide libraries of *E. coli* TF and *E. coli* ClpX were synthesized by Fmoc chemistry on a cellulose membrane using an ASP222 Autospot robot (Intavis AG) essentially as described^67^. The membrane was blocked with 1× Superblock (Pierce) in TBS buffer (20 mM Tris-HCl pH 7.5, 137 mM NaCl) containing 0.05% Tween-20 and probed for 1 h at 22 °C with 0.5 µM of purified His-ClpXΔZBD and 1 mM ATPγS or 0.5 µM of His-TF in TBS buffer containing 0.05% Tween-20, 10% Superblock, and 5% sucrose. The membrane was washed three times for 10 min in TBS buffer containing 0.05% Tween-20 and 50 µM ATPγS (His-ClpXΔZBD). His-protein binding was detected by probing the membrane directly with an anti-6×His monoclonal antibody conjugated to horseradish peroxidase used at a dilution of 1:5000 (mAB-HRP; BD Bioscience) and visualized by chemiluminescence. Non-specifically bound peptides were eliminated by subtracting spots that were also detected in a control experiment using non-tagged protein and mAB-HRP. Protein binding motifs were mapped onto the available crystal structure for TF (PDB: 1W26)^32^ and the cryoEM structure of ClpX∆ZBD (PDB: 6PP5)^33^ for visualization.

### NMR experiment

For NMR titration experiments, [U-^15^N; Ala-^13^CH_3_; Met-^13^CH_3_; Ile-δ1-^13^CH_3_; Leu/Val-^13^CH_3_/^13^CH_3_]-labeled TF_PA_ (TF 113-432) and [U-^15^N]-labeled TF_N_ (TF 1-117) were expressed in *E. coli* BL21(DE3) cells and purified as described previously^8,31^. TF_PA_ (113-432) was cloned into the pCold vector (Takara Bio) and fused to His_6_-tag. TF_N_ (1–117) was cloned into pET16b vector (Novagen) and fused to His_6_-MBP and a TEV protease cleavage site. Cells were grown in a medium containing ^15^NH_4_Cl (1 gL^−1^) and ampicillin (100 mg L^−1^). For the preparation of ^1^H–^13^C methyl-labeled samples, α-ketobutyric acid (50 mg L^−1^) and α-ketoisovaleric acid (85 mg L^−1^), [^13^CH_3_] methionine (50 mg L^−1^), [2-^2^H, ^13^CH_3_] alanine (50 mg L^−1^) were added to the culture 1 h before the addition of IPTG. Protein expression was induced by the addition of 0.5 mM IPTG at OD_600_ ~0.6, followed by ~16 h of incubation at 18 °C. Cells were harvested and resuspended in the lysis buffer containing 50 mM Tris-HCl pH 8.0, 500 mM NaCl. Cells were disrupted by a sonicator and centrifuged at 50,000×*g* for 45 min. Proteins were purified using Ni Sepharose 6 Fast Flow resin (GE Healthcare). In the case of TF_N_ that contains TEV cleavage site, the His_6_-MBP tag was removed by TEV protease at 4 °C (incubation for 16 h). Proteins were further purified by gel filtration using Superdex 75 16/60 or 200 16/60 columns (GE Healthcare) in 25 mM 50 mM Tris-HCl (pH 7.5), 400 mM NaCl, 10% glycerol, 5 mM β-mercaptoethanol.

ClpX ZBD and AAA+ were cloned into pProEXHTa and expressed in *E. coli* BL21(DE3) cells. Protein expression was induced by the addition of 1 mM IPTG at OD_600_ ~0.6, followed by ~16 h of incubation at 18 °C. Proteins were purified by the same procedure as TF_N_ as described above, but 100 μM ZnCl_2_ was supplemented to ZBD prior to size exclusion chromatography.

All of the proteins were prepared in the NMR buffer containing 50 mM Tris-HCl (pH 7.5), 200 mM KCl, 25 mM MgCl_2_, 2% glycerol, 5 mM β-mercaptoethanol. TF domains were concentrated to ~0.2 mM. The AAA+ domain was added to the TF domains in the presence of ADP. NMR experiments were performed on a Bruker 600 MHz NMR at 22 °C. Perturbation of the backbone and side chain methyl resonances were monitored using 2D ^1^H-^15^N transverse relaxation-optimized spectroscopy (TROSY) spectra and ^1^H-^13^C heteronuclear multiple quantum coherence (HMQC), respectively. Spectra were processed using the NMRPipe software^68^. Data analysis was performed by Olivia software (http://fermi.pharm.hokudai.ac.jp/olivia/). The resonance assignments were obtained from the BMRB Entries 19835 and 5665 for TF and ClpX ZBD, respectively. The perturbations of the resonances were evaluated by chemical shift change or intensity change. CSPs for backbone amide groups were calculated using the equation

1 $${\Delta}\delta = \sqrt {{\Delta}\delta _{\mathrm{H}}^2 + \left( {0.2{\Delta}\delta _{\mathrm{N}}} \right)^2},$$

where ∆*δ*_H_ and ∆*δ*_N_ are the chemical shift changes of ^1^H and ^15^N, respectively. CSPs for methyl groups were calculated by the equation

2 $${\Delta}\delta = \sqrt {\left( {\frac{{{\Delta}\delta _{\mathrm{H}}}}{\alpha }} \right)^2 + \left( {\frac{{{\Delta}\delta _{\mathrm{C}}}}{\beta }} \right)^2},$$

where ∆*δ*_H_ and ∆*δ*_C_ are the chemical shift changes of ^1^H and ^13^C, respectively, upon the addition of the ligand, and *α* and *β* are the chemical shift distribution of ^1^H and ^13^C, respectively, of methyl groups as reported in the Biological Resonance Data Bank (http://www.bmrb.wisc.edu). The perturbed resonances were mapped onto the structure of TF or ClpX ZBD. The cutoff of the CSP mapping was determined by ∆*δ*_ave_ + ∆*δ*_SD_, where ∆*δ*_ave_ and ∆*δ*_SD_ are average and standard deviation, respectively, of the chemical shift change by the addition of the ligand. The cutoff of the intensity perturbation mapping was determined by *r*_int,ave_ − *r*_int,SD_, where *r*_int,ave_ and *r*_int,SD_ are average and standard deviation, respectively, of the intensity ratio.

### GAPDH anti-aggregation assay

Aggregation of denatured GAPDH from rabbit muscle (Sigma; G-2267) was measured as described previously^8,31^. Totally, 125 µM GAPDH was denatured by 3 M guanidine-HCl in 20 mM potassium phosphate (pH 7.0) 100 mM KCl, 4 mM 2-mercaptoethanol, 0.5 mM EDTA, 0.05% NaN_3_ for overnight at 4 °C. Upon 50-fold dilution of the denatured enzyme into the buffer without guanidine-HCl, aggregation was monitored by 90° light scattering at 620 nm in a spectrofluorometer (FP-8500, JASCO Corporation) in the absence or presence of 1 µM of TF or TF mutant. The measurement was carried out at 20 °C.

### In vitro degradation assays

Degradation assays were carried out in the absence or presence of TF. Degradation reaction mixture contained 1.2 μM ClpP, 3.9 μM substrate, 0 or 5.0 μM TF, and an ATP regeneration system (13 units/mL of creatine kinase and 16 mM creatine phosphate) in PD buffer (25 mM HEPES, pH 7.5, 5 mM MgCl_2_, 5 mM KCl, 0.03% (w/v) Tween 20, and 10% glycerol). All concentrations are those of protomers. Components were incubated at 37 °C for 3 minutes before adding 1.0 μM ClpX to start the reaction. At given time points, aliquots were withdrawn and mixed with 4× Laemmli buffer to stop the reaction. Proteins were then resolved on SDS-PAGE gels and visualized by Coomassie blue staining. Protein bands on the gels were quantified using GelEval (FrogDance). Anti-MuA (dilution 1:3000) antiserum was kindly provided by Dr. George Chaconas (University of Calgary, Canada). GFP-SsrA degradation was monitored by fluorescence using a Fluorolog spectrofluorometer (JobinYvon) with excitation wavelength set at 395 nm and emission wavelength set at 509 nm. RpoS degradation assays were performed using 3.9 µM of RpoS, 0.005 µM RssB, and 20 µM lithium potassium acetyl phosphate as phosphate donor^50^.

### ATPase activity measurement

The effect of TF on ClpX ATPase activity was measured using a coupled assay^69^ in a 96-well microtiter plate read using the Molecular Devices SpectraMax 340 PC 384 Microplate Reader. The reaction volume was 150 µL and assays were performed by preincubating 1.0 µM ClpX, various concentrations of TF, 0.2 mM NADH, 3.0 mM phosphoenolpyruvate, 4.7 units of pyruvate kinase, and 7.4 units of lactate dehydrogenase in PD buffer at 37 °C for 3 min. ClpP was added at 1.2 µM, and λO at 3.9 µM if needed. After preincubation for 3 min, 5 mM ATP was added to start the reaction and the change in absorbance at 340 nm was measured for 20 min at 37 °C. ATPase assays using ClpA were performed under the same concentration of the different components but in a high salt buffer (25 mM HEPES, pH 7.5, 20 mM MgCl_2_, 300 mM KCl, 0.03% (w/v) Tween 20, and 10% glycerol). ATP hydrolysis was calculated by measuring the disappearance of NADH at 340 nm, assuming 1:1 correspondence between ADP formation and NADH oxidation. The extinction coefficient used for NADH is 6220 M^−1^ cm^−1^ (ref. ^69^). Each reaction was performed at least three times.

### SPR assay

SPR measurements were carried out on a Biacore X from GE Healthcare; all experiments were done at 22 °C. TF was immobilized on a CM5 chip (GE Healthcare) using the Biacore Amine Coupling kit. The analyte was injected at a flow rate of 20 μL/min in running buffer (10 mM HEPES, pH 7.5, 150 mM NaCl, 3 mM EDTA, and 0.005% (w/v) P20 surfactant), and adequate injection time (90 s) was allowed for the interaction to reach steady-state if possible. To regenerate the surface between binding experiments, 2 M NaCl was injected for 1 min and washed with running buffer. From the results of the sensorgrams, response units at steady state were plotted against the corresponding analyte concentrations to generate the binding curve, and the *K*_d_ was calculated using WinCurveFit program assuming one-site Langmuir binding model for most binding experiments.

### Calculation of protomer distributions in mixed ClpX and ClpX∆ZBD complexes

In order to calculate the subunit composition upon mixing ClpX and ClpX∆ZBD in defined ratios (Fig. 3e), the following combinatorial statistics were used. We use *p* to represent the fractional concentration of ClpX (containing a trimer-of-dimers of ZBD)

3 $$p = \frac{{{\mathrm{[ClpX]}}}}{{\left[ {{\mathrm{ClpX}}} \right]{\mathrm{ + [ClpX}}{\Delta}{\mathrm{ZBD]}}}}.$$

The concentrations are those of protomers. The mixed ClpX:ClpX∆ZBD hexameric complexes formed have full-length ClpX composition (i.e., ZBD_2_ composition) that can be calculated, assuming random mixing, according to the following formula.

4 $$C_k^3p^k\left( {1 - p} \right)^{3 - k},$$

where *k* is the number of full-length ClpX subunits in the complex and

5 $$C_k^3 = \frac{{3^!}}{{k!\left( {3 - k} \right)!}}.$$

### Pulldowns using ClpP^trap^-SPA

The ClpP^trap^-SPA and the T7 polymerase containing plasmid pT7pol26 were cotransformed into different MC4100 mutant strains. For control experiments, a vector expressing WT ClpP was transformed into ∆*clpP*∆*clpA*∆*smpB* strain. 11-mL cultures were incubated overnight at 37 °C. The next day, these cultures were used to inoculate 4 L cultures, which were then grown to mid-log phase (OD_600_ = 0.4). Cultures were induced for 4 h using 1 mM IPTG at 30 °C. Cell pellets were then resuspended in lysis buffer (25 mM Tris-HCl pH 7.5, 300 mM NaCl, 10% glycerol, and 1 mM DTT containing protease inhibitor cocktail (P8849; Sigma-Aldrich) and lysed using French press followed by centrifugation at 13,000×*g* for 30 min. The supernatants were collected for pull-down assays.

Totally, 200 μL of pre-washed FLAG antibody resin used at a dilution of 1:5000 (A2220; Sigma-Aldrich) was added to 20 mL supernatant and incubated with gentle rocking overnight at 4 °C. Beads were then transferred into flow columns and washed extensively with buffer (25 mM TrisHCl pH 7.5, 300 mM NaCl, 10% Triton X-100, 0.5 mM DTT, protease inhibitor cocktail 25×). Proteins were eluted from the FLAG beads using 1 mg/ml FLAG peptide (Sigma-Aldrich). Eluted proteins were subsequently digested with trypsin and identified using an Orbitrap mass spectrometer (ThermoFisher Scientific). The resulting MS/MS spectra were then queried against the protein-coding sequences of *E. coli* using the SEQUEST search engine. Matches with high confidence (>95% probability) were then evaluated using spectral counts and probability scores computed by the STATQUEST algorithm. Only proteins with three or more unique peptides were considered. To quantify the data, the normalized spectral abundance factor method was used^70^, which employs spectral counts normalized to protein length and to the total spectral count in each sample.

### Monitoring λO and GFP-ssrA degradation in cells

Plasmid pRLM266 expressing untagged λO under the T7 promoter was transformed into WT, *tig::cat*, and *clpX::cat* MC4100 strains. The strains were cotransformed with the pT7pol26 plasmid expressing the T7 polymerase^58^. Cells were grown in LB broth at 37 °C until mid-log phase (OD_600_ = 0.6); IPTG was then added at a final concentration of 0.4 mM to induce λO expression for 2 h. Tetracycline was added at a final concentration of 30 μg/mL to inhibit translation (time 0), and 100 µL of cells were withdrawn after the addition of tetracycline at 0, 10, 13, 30, 45, 60, and 90 min. Cell density was measured using OD_600_ for each time point. Cells were pelleted and resuspended in 1× SDS loading buffer with equal cell density. Whole-cell lysates were subjected to western blot analysis using rabbit antisera specific for λO, ClpX, ClpP, and TF proteins. About 10^5^ cells were loaded for each time point to monitor the degradation of λO proteins. The same procedure was performed to monitor GFP-ssrA levels.

For monitoring λO-SecM degradation, pET15b λO-SecM was transformed into MC4100 strains also containing pT7pol26 plasmid, and the same procedures were followed as above except that 10 times more cells (10^6^) were loaded for western blot analysis to monitor λO-SecM due to lower expression level of the fusion protein.

For ribosome fractionation, experiments were carried out as described above. For each time point, 10 mL of cells were taken, pelleted, and resuspended in buffer R [10 mM Tris-HCl, pH 7.5, 10 mM MgCl_2_, 60 mM NH_4_Cl, 400 mM potassium acetate, 1:1000 dilution of RNAse inhibitor (Promega), 1 U/mL RNAse free DNase I, and EDTA-free protease inhibitor cocktail from Sigma (1 tablet in 50 mL buffer)] to the same cell density. Cells were lysed by sonication and debris was spun down at 20,000*g* for 30 min. Equal amounts of supernatant were loaded on a 3-fold or greater volume of sucrose cushion (0.7 M sucrose, 10 mM TrisHCl, pH 7.5, 10 mM MgCl_2_, 60 mM NH_4_Cl, 400 mM KOAc, and 1:1000 dilution of RNAse inhibitor) and centrifuged at 148,379×*g* (average) for 5 h using Beckman MLS-50 ultracentrifuge. Pelleted ribosomes were resuspended in buffer S (20 mM TrisHCl, pH 7.5, 10 mM KCl, 10 mM MgCl_2,_ and 10% sucrose). Samples containing equal amounts of ribosomal material were separated on SDS-PAGE gels and analyzed by western blot.

### Monitoring λO degradation in lysogens

WT and *tig::cat* MC4100 strains were lysogenized with the heat-inducible prophage λ*cI*857Sam7. The lysogens were grown in M63 minimal media supplemented with 0.2% glucose at 30 °C until mid-log phase (OD_600_ = 0.6) and then heat shocked at 42 °C for 8 min to induce phage replication. After heat shock, cells were pulse labeled with 200 µCi of [^35^S] methionine (PerkinElmer, 1000 Ci/mmol) for 2 min, and chased with 200 µg/mL nonradioactive methionine at 37 °C for 30 min. At different time points, samples were withdrawn during the chase period, and λO protein was immunoprecipitated using α-λO polyclonal rabbit antiserum (dilution of 1:3000) and 300 µg Protein A-Acrylic beads from *Staphylococcus aureus* (Sigma).

### Monitoring degradation of newly synthesized proteins

WT, *tig::cat*, and *tig::cat* MC4100 cells complemented with a plasmid expressing *tig* under its own promoter were grown at 37 °C in M9 minimal medium supplemented with 0.4% glucose (MG medium) until mid-log phase, OD_600_ = 0.6. Cells were then pulse-labeled with 60 µCi/mL [^35^S] methionine (PerkinElmer, 1000 Ci/mmol) for 2 min and chased with 0.5 mg/mL non-radioactive methionine for 3 min. To remove excess [^35^S] methionine, cells were washed three times in MG medium containing non-radioactive methionine and resuspended in MG medium with non-radioactive methionine and 30 µg/mL tetracycline to inhibit translation. Aliquots of cells were taken at different time points, and samples were precipitated with TCA. To determine the relative amount of degraded proteins, radioactivity in the TCA-soluble fraction, containing small peptides, and TCA-insoluble fraction, containing larger polypeptides, was measured by liquid scintillation counting (Beckman Coulter LS6500 Multipurpose Scintillation Counter). TCA-insoluble fractions were resolubilized in 8 M urea before measuring radioactivity. The percentage of degraded proteins was calculated by taking the ratio of the counts from TCA-soluble fraction over total scintillation counts (TCA-soluble plus TCA-insoluble). For heat shock and AZC treatment conditions, the procedures were the same except that cells were heated at 42 °C for 10 min or treated with 100 µg/mL AZC before pulse labeling with [^35^S] methionine, respectively.

### Reporting summary

Further information on experimental design is available in the Nature Research Reporting Summary linked to this paper.

## Supplementary information

Supplementary Information

Peer Review File

Reporting Summary

## Data Availability

The data that support this study are present in the paper and Supplementary information, and are available from the corresponding author upon request. Source data are provided with this paper.

## Source data

Source Data
