# Author Correction: Functional cooperativity between the trigger factor chaperone and the ClpXP proteolytic complex

**DOI:** 10.1038/s41467-021-23109-9

**Published:** 2021-05-06

**Authors:** Kamran Rizzolo, Angela Yeou Hsiung Yu, Adedeji Ologbenla, Sa Rang Kim, Haojie Zhu, Koichiro Ishimori, Guillaume Thibault, Elisa Leung, Yi Wen Zhang, Mona Teng, Marta Haniszewski, Noha Miah, Sadhna Phanse, Zoran Minic, Sukyeong Lee, Julio Diaz Caballero, Mohan Babu, Francis T. F. Tsai, Tomohide Saio, Walid A. Houry

**Affiliations:** 1 Department of Biochemistry, University of Toronto, Toronto, ON M5G 1M1 Canada; 2 Graduate School of Chemical Sciences and Engineering, Hokkaido University, Sapporo, Hokkaido 060-8628 Japan; 3 Department of Chemistry, Faculty of Science, Hokkaido University, Sapporo, Hokkaido 060-0810 Japan; 4 School of Biological Sciences, Nanyang Technological University, Singapore, 637551 Singapore; 5 The Donnelly Centre, University of Toronto, Toronto, ON M5S 3E1 Canada; 6 Department of Biochemistry, University of Regina, Regina, Saskatchewan S4S 0A2 Canada; 7 Department of Chemistry and Biomolecular Science, University of Ottawa, John L. Holmes Mass Spectrometry Facility, Ottawa, ON K1N 1A2 Canada; 8 Department of Biochemistry and Molecular Biology, Baylor College of Medicine, Houston, TX 77030 USA; 9 Department of Cell and Systems Biology, University of Toronto, Toronto, ON M5S 3G5 Canada; 10 Department of Molecular and Cellular Biology, Baylor College of Medicine, Houston, TX 77030 USA; 11 Department of Molecular Virology and Microbiology, Baylor College of Medicine, Houston, TX 77030 USA; 12 Institute of Advanced Medical Sciences, Tokushima University, Tokushima, 770-8503 Japan; 13 Department of Chemistry, University of Toronto, Toronto, ON M5S 3H6 Canada; 14 Present Address: Dewpoint Therapeutics, 6 Tide Street, Boston, MA 02210 USA; 15 Present Address: Pfizer Inc., 401 N Middletown Rd, Pearl River, NY 10965 USA

**Keywords:** Protein quality control, Chaperones

Correction to: *Nature Communications* 10.1038/s41467-020-20553-x, published online 12 January 2021.

The original version of this Article contained an error in the spelling of the author Sa Rang Kim, which was incorrectly given as Sa-Rang Kim.

This has now been corrected in both the PDF and HTML versions of the Article.

