# Supplementary material for: Functional cooperativity between the trigger factor chaperone and the ClpXP proteolytic complex
Source: Nat Commun. 2021 Jan 12;12:281. doi: 10.1038/s41467-020-20553-x (PMC7804408; doi:10.1038/s41467-020-20553-x)
Supplement: Supplementary file 1 — Supplementary Information [file 41467_2020_20553_MOESM1_ESM.pdf]

Supplementary Information

Functional cooperativity between the trigger factor chaperone and the ClpXP proteolytic complex

Kamran Rizzolo[1,*,#], Angela Yeou Hsiung Yu[1,*], Adedeji Ologbenla[1], Sa-Rang Kim[1], Haojie Zhu[2], Koichiro Ishimori[2,3], Guillaume Thibault[1,4], Elisa Leung[1], Yi Wen Zhang[1], Mona Teng[1], Marta Haniszewski[1], Noha Miah[1], Sadhna Phanse[1,5,6], Zoran Minic[6,7], Sukyeong Lee[8], Julio Diaz Caballero[9], Mohan Babu[6], Francis T. F. Tsai[8,10,11], Tomohide Saio[2,3] and Walid A. Houry[1,12, †]

[1]Department of Biochemistry, University of Toronto, Toronto, Ontario M5G 1M1, Canada

[2]Graduate School of Chemical Sciences and Engineering, Hokkaido University, Sapporo, Hokkaido 060-8628, Japan

[3]Department of Chemistry, Faculty of Science, Hokkaido University, Sapporo, Hokkaido 060-0810, Japan

[4]Temasek Life Sciences Laboratory, National University of Singapore, Singapore

[5]The Donnelly Centre, University of Toronto, Toronto, Ontario M5S 3E1, Canada

[6]Department of Biochemistry, University of Regina, Regina, Saskatchewan S4S 0A2, Canada

[7]Department of Chemistry and Biomolecular Science, University of Ottawa, John L. Holmes, Mass Spectrometry Facility, Ottawa, Ontario K1N 1A2, Canada

[8]Department of Biochemistry and Molecular Biology, Baylor College of Medicine, Houston, TX 77030, USA

[9]Department of Cell and Systems Biology, University of Toronto, Ontario M5S 3G5, Canada

[10]Department of Molecular and Cellular Biology, Baylor College of Medicine, Houston, Texas 77030, USA

[11]Department of Molecular Virology and Microbiology, Baylor College of Medicine, Houston, Texas 77030, USA

[12]Department of Chemistry, University of Toronto, Toronto, Ontario M5S 3H6, Canada

*These authors contributed equally to this work.

[#]Current affiliation: Dewpoint Therapeutics, 6 Tide Street, Boston, MA 02210, USA

[†]Corresponding author. W. A. Houry, Department of Biochemistry, University of Toronto, 661 University Avenue, MaRS Centre, West Tower, Room 1612, Toronto, ON M5G 1M1, Canada. Tel.: (416) 946-7141; Fax: (416) 978-8548; Email: walid.houry@utoronto.ca

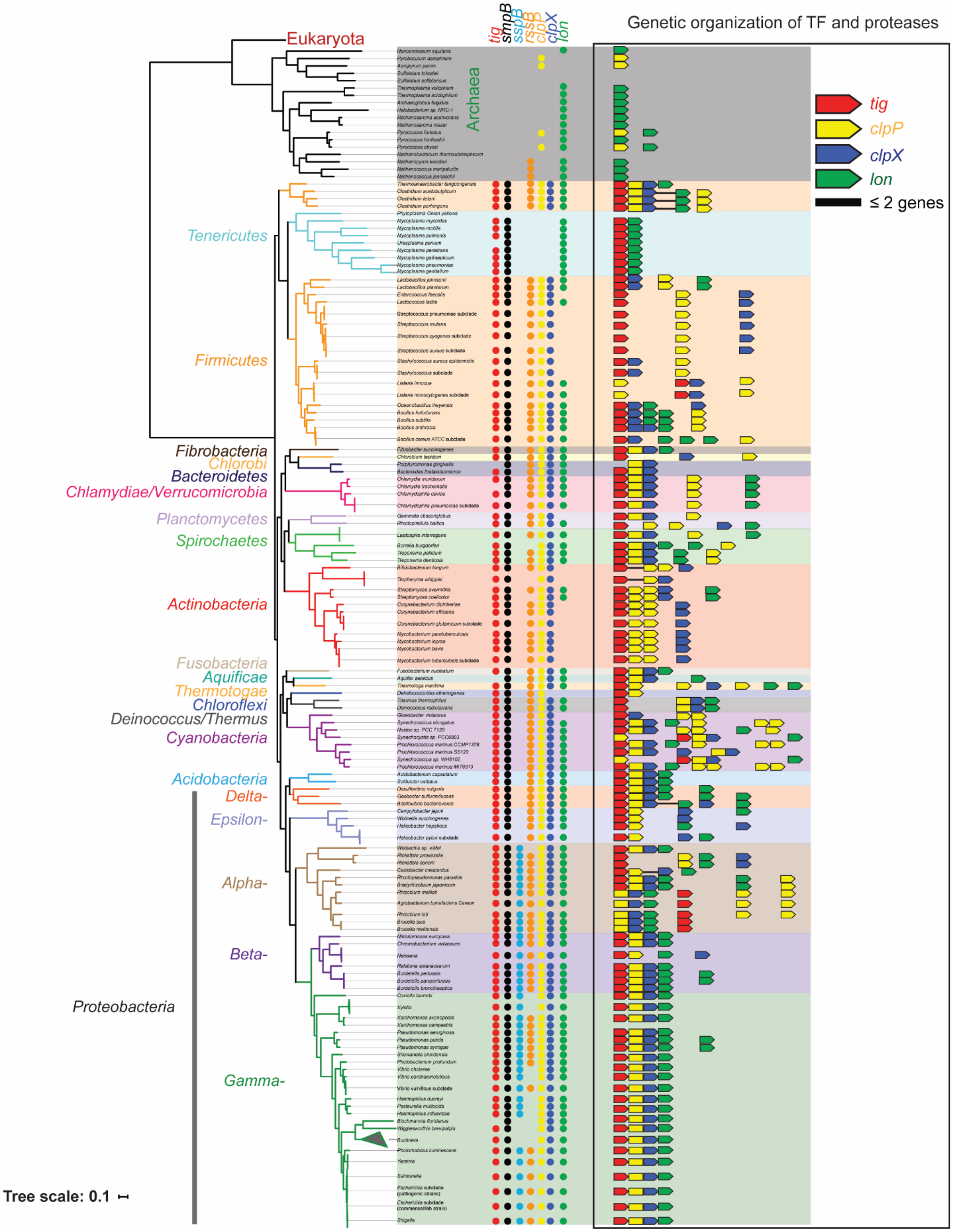
Genetic organization of TF and proteases
tig
smpB
sspB
rssB
clpP
clpX
lon
Eukaryota
Archaea
Tenericutes
Firmicutes
Fibrobacteria
Chlorobi
Bacteroidetes
Chlamydiae/Verrucomicrobia
Planctomycetes
Spirochaetes
Actinobacteria
Fusobacteria
Aquificae
Thermotogae
Chloroflexi
Deinococcus/Thermus
Cyanobacteria
Acidobacteria
Delta-
Epsilon-
Alpha-
Beta-
Proteobacteria
Gamma-
tig
clpP
clpX
lon
≤ 2 genes
Tree scale: 0.1

Supplementary Figure 1. Phylogeny of *tig, clpXP* and *lon*.

Tree of life[15, 73] highlighting the presence (dot) or absence (no dot) of the *tig* (red), *smpB* (black), *sspB* (light blue), *rssB* (orange), *clpP* (yellow), *clpX* (blue) and *lon* (green) genes in each genome. Also shown are the relative genomic position of these genes plotted in the 5′ to 3′ orientation indicated from left to right. The thick lines between *clpP* and *clpX*, between *tig* and *clpP*, or between *clpX* and *lon* indicate the presence of 1 and 2 genes in between. Data were collected from NCBI, ExPASy (2016 version), KEGG and UniProt databases for all available bacterial genomes with representatives shown in the figure. Gene presence or absence was also confirmed from the literature in some cases. Bacterial phyla are color coded along with the respective species branches. Few species do not have genomic information available so were left blank. Tree scale represents the number of substitutions per site.

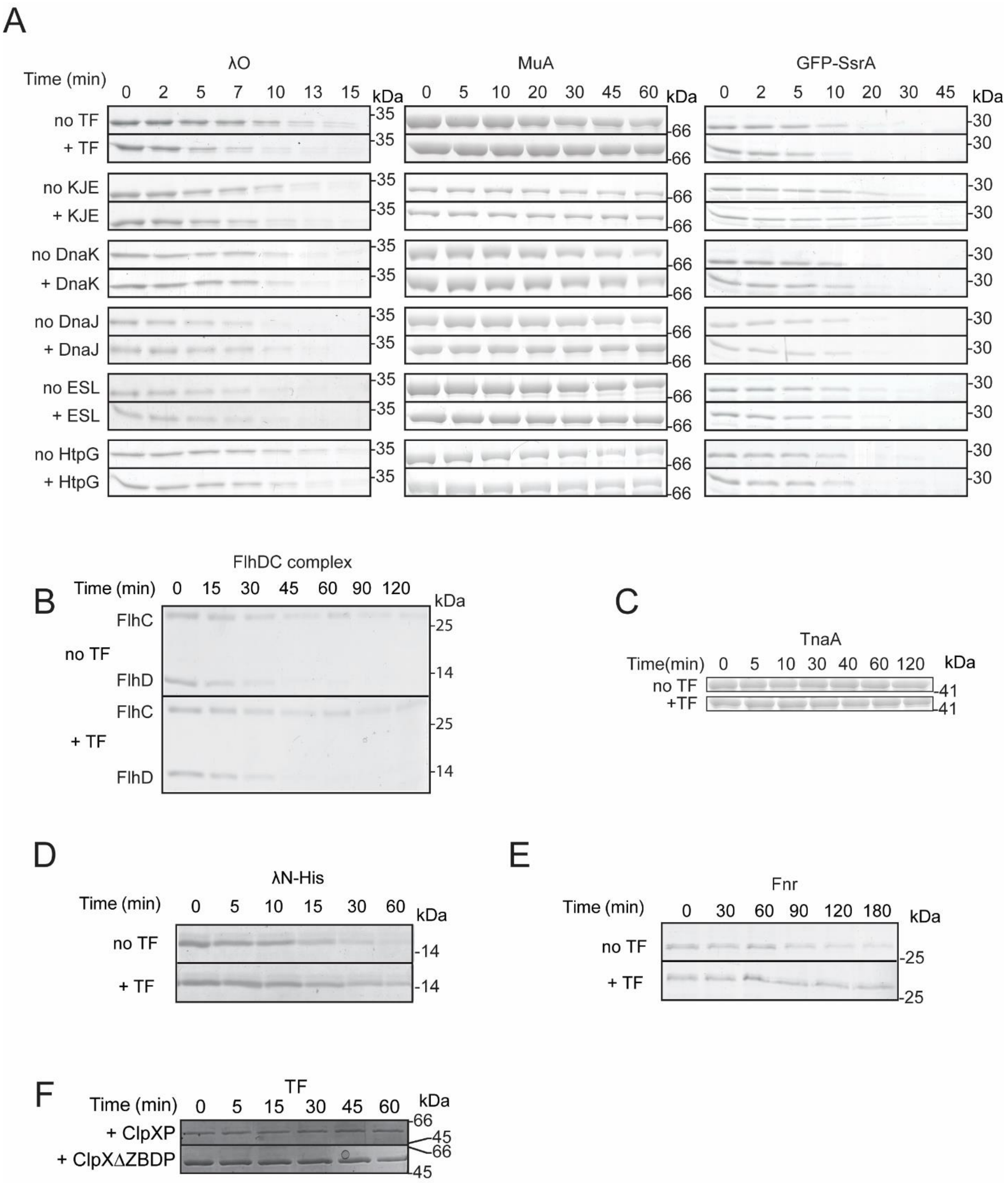

A
λO
MuA
GFP-SsrA
Time (min)
0 2 5 7 10 13 15 kDa
0 5 10 20 30 45 60 kDa
0 2 5 10 20 30 45 kDa
no TF
+ TF
no KJE
+ KJE
no DnaK
+ DnaK
no DnaJ
+ DnaJ
no ESL
+ ESL
no HtpG
+ HtpG
35
66
30
B
FlhDC complex
Time (min) 0 15 30 45 60 90 120
kDa
FlhC
FlhD
no TF
+ TF
25
14
C
TnaA
Time(min) 0 5 10 30 40 60 120
kDa
no TF
+TF
41
D
λN-His
Time (min) 0 5 10 15 30 60
kDa
no TF
+ TF
14
E
Fnr
Time (min) 0 30 60 90 120 180
kDa
no TF
+ TF
25
F
TF
Time (min) 0 5 15 30 45 60
kDa
+ ClpXP
+ ClpXΔZBDP
66
45

Supplementary Figure 2. Effect of TF on ClpXP-mediated degradation.

(A) The effect of chaperones on the ClpXP-mediated degradation of model substrates. Representative SDS-PAGE gels, visualized by Coomassie staining, are shown for the degradation of λO, MuA, and GFP-SsrA in the absence or presence of TF, DnaJ/DnaK/GrpE, DnaK, DnaJ, GroEL/GroES, and HtpG respectively. At least three independent reactions were performed for A-F.

(B-E) Effect of TF on the ClpXP-dependent degradation of FlhDC complex, TnaA, λN-His, and Fnr proteins.

(F) Gels showing that TF is not degraded by ClpP in the presence of ClpX or ClpXΔZBD. Source data are provided as a Source Data file.

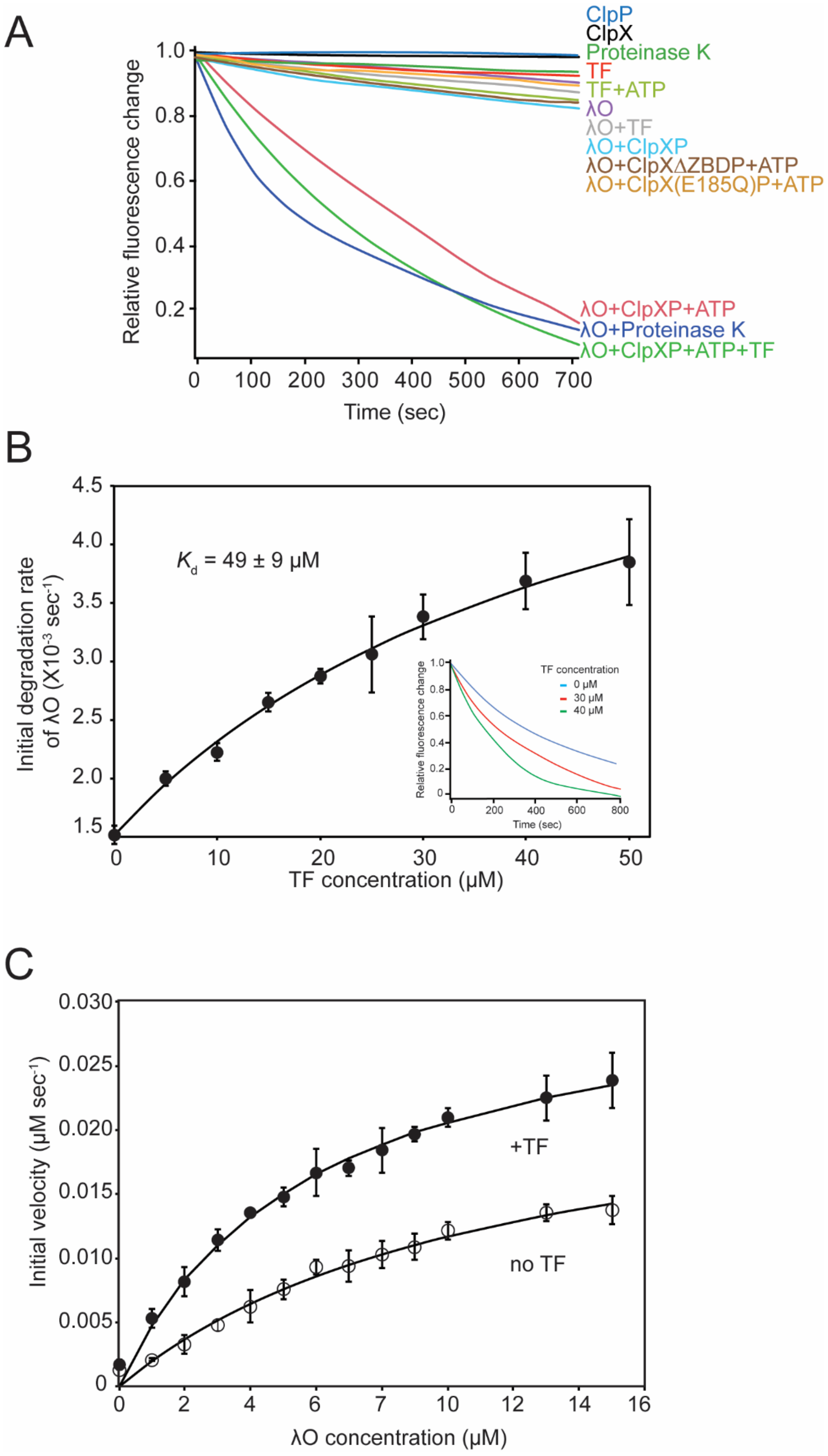
A
Relative fluorescence change
Time (sec)
ClpP
ClpX
Proteinase K
TF
TF+ATP
λO
λO+TF
λO+ClpXP
λO+ClpXΔZBDP+ATP
λO+ClpX(E185Q)P+ATP
λO+ClpXP+ATP
λO+Proteinase K
λO+ClpXP+ATP+TF
B
Initial degradation rate of λO (X10-3 sec-1)
K_d = 49 ± 9 μM
TF concentration (μM)
Relative fluorescence change
TF concentration
0 μM
30 μM
40 μM
Time (sec)
C
Initial velocity (μM sec-1)
+TF
no TF
λO concentration (μM)

Supplementary Figure 3. Kinetic analysis of the effect of TF on ClpXP-mediated degradation of λO.

(A) Tryptophan fluorescence (λ_{em} = 280nm/λ_{ex} = 350nm) measurements as a function of time for the indicated proteins and reactions.

(B) Initial degradation rate of λO by ClpXP in the presence of increasing TF concentration. The inset shows examples of such degradation reactions. K_d is derived from the fitted curve. Measurements at higher protein concentrations needed to reach saturation were not possible because of protein aggregation.

(C) Michaelis-Menten plot of the variation of the degradation rates as a function of concentration of λO in the absence or presence of TF (related to Figure 1C). All reactions were repeated at least 3 times to obtain the averages and standard deviations. Source data are provided as a Source Data file.

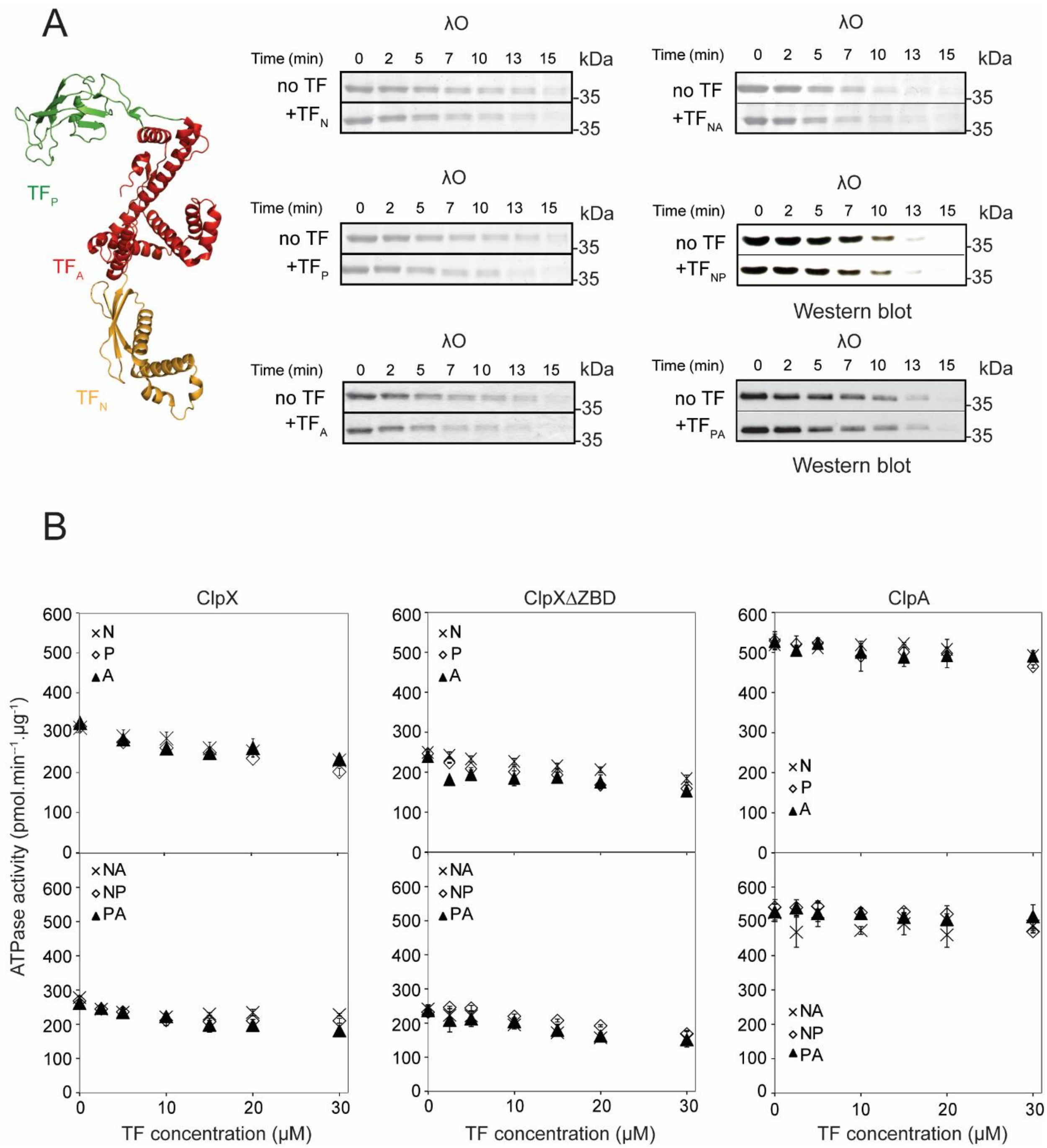
A
TFP
TFA
TFN
λO
Time (min)
0 2 5 7 10 13 15
kDa
no TF
+TFN
+TFP
+TFA
+TFNA
+TFNP
+TFPA
35
Western blot
B
ClpX
ClpXΔZBD
ClpA
×N
◇P
▲A
×NA
◇NP
▲PA
ATPase activity (pmol.min⁻¹.µg⁻¹)
TF concentration (µM)

Supplementary Figure 4. Effect of TF domains on ClpXP-mediated substrate degradation and on ClpX ATPase activity.

(A) Coomassie blue stained gels showing the ClpXP-dependent degradation of λO in the presence of different TF domains. On the left is a ribbon diagram of TF. For TF_{NP} and TF_{PA}, the degradation was monitored by western blot analysis since TF_{NP} and TF_{PA} have a similar mass as λO. At least three independent reactions were performed.

(B) The ATPase activities of ClpX, ClpXΔZBD and ClpA were measured in the presence of increasing concentrations of different TF domains. Error bars represent the standard deviations from n=3 independent reactions. Source data are provided as a Source Data file.

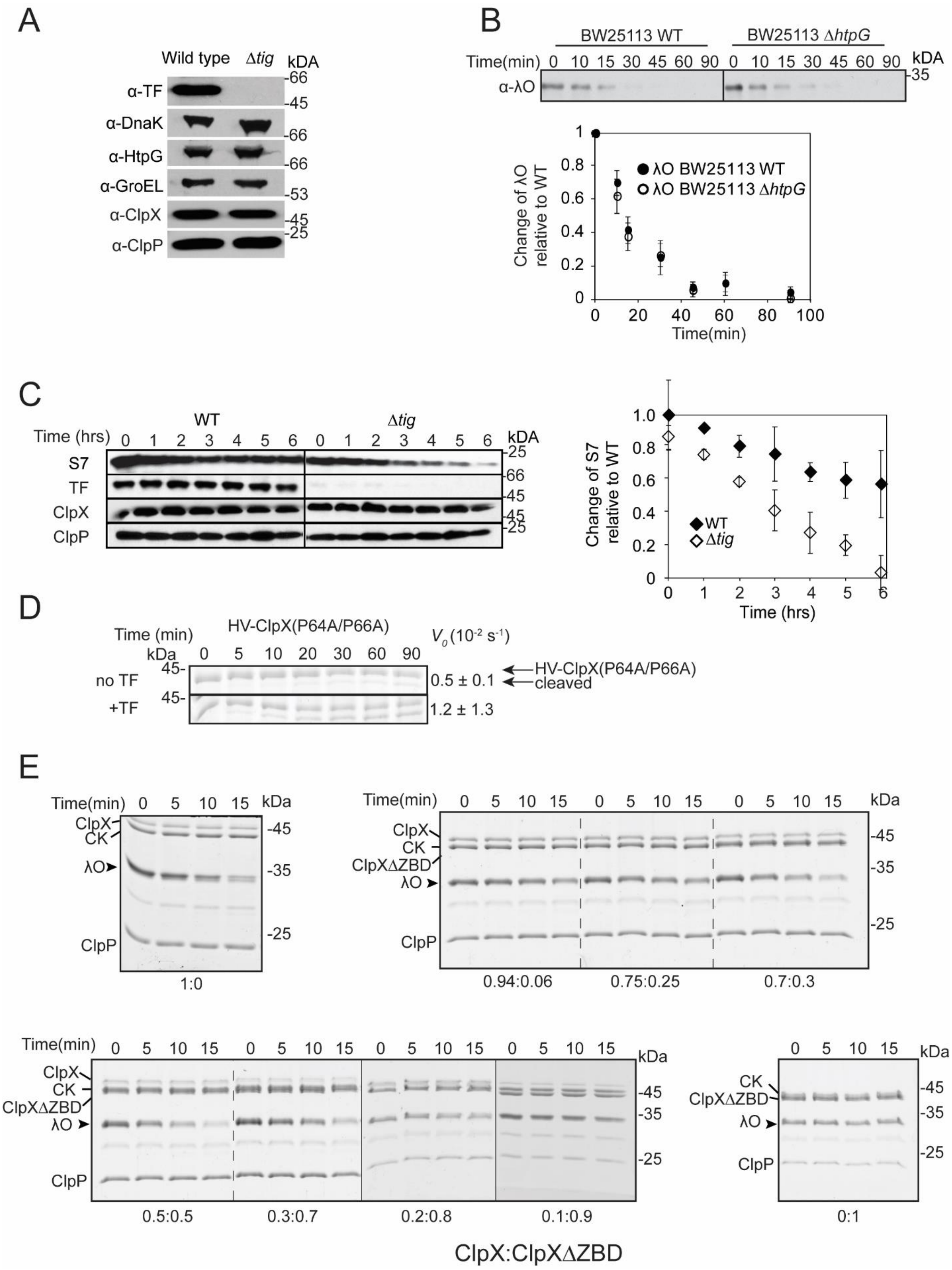
A
Wild type
Δtig
kDA
α-TF
α-DnaK
α-HtpG
α-GroEL
α-ClpX
α-ClpP
B
BW25113 WT
BW25113 ΔhtpG
Time(min) 0 10 15 30 45 60 90 0 10 15 30 45 60 90
α-λO
λO BW25113 WT
λO BW25113 ΔhtpG
Change of λO relative to WT
Time(min)
C
WT
Δtig
Time (hrs) 0 1 2 3 4 5 6 0 1 2 3 4 5 6
S7
TF
ClpX
ClpP
Change of S7 relative to WT
WT
Δtig
Time (hrs)
D
Time (min)
HV-ClpX(P64A/P66A)
V_0 (10^{-2} s^{-1})
no TF
+TF
0.5 ± 0.1
1.2 ± 1.3
HV-ClpX(P64A/P66A)
cleaved
E
Time(min) 0 5 10 15
ClpX
CK
ClpXΔZBD
λO
ClpP
1:0
0.94:0.06
0.75:0.25
0.7:0.3
0.5:0.5
0.3:0.7
0.2:0.8
0.1:0.9
0:1
ClpX:ClpXΔZBD

Supplementary Figure 5. Effect of TF on ClpXP-dependent degradation in cells and effect of mixed ClpX complexes on the rate of degradation of λO.

(A) Immunoblot staining of the indicated chaperones in *E. coli* MC4100 WT and Δ*tig* strains.

(B) Immunoblot staining of *in vivo* degradation of λO expressed in *E. coli* WT and Δ*htpG* strains. Error bars represent the standard deviations from n=3 independent replicates.

(C) Effect of TF on the *in vivo* degradation of S7. The assay was performed as described previously for λO. 10^6 cells were loaded for each time point for western blot analysis using sheep antisera for S7 (kind gift from Dr. Knud Nierhaus, Max Planck Institute for Molecular Genetics, Germany). Quantification of substrate bands are shown on the right. Error bars represent the standard deviations from n=3 independent replicates.

(D) *In vitro* clipping and degradation of HV-ClpX(P64A/P66A) mutant by ClpP in the presence of TF. V_0 refers to initial rate of disappearance of HV-ClpX(P64A/P66A). Standard deviations were calculated from at least three independent experiments.

(E) Representative SDS-PAGE gels of the degradation of λO as a function of time in the presence of ClpP and ClpX:ClpXΔZBD mixed in the following ratios: 1:0, 0.94:0.06, 0.75:0.25, 0.7:0.3, 0.5:0.5, 0.3:0.7, 0.2:0.8, 0.1:0.9 and 0:1, which resulted in the complex proportions shown in Figure 3E. No TF is present in the reactions. At least three independent reactions were performed. Source data are provided as a Source Data file.

A

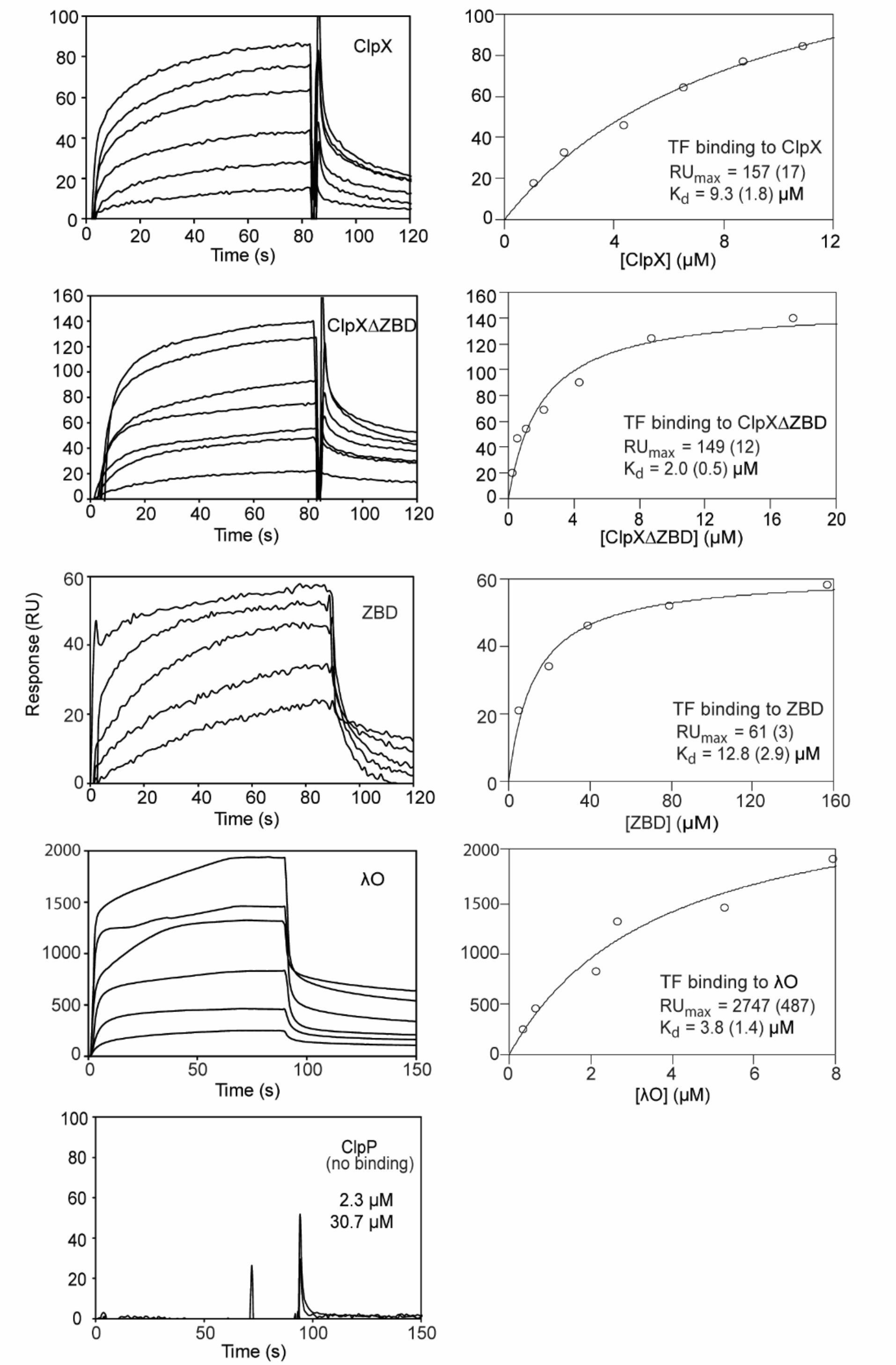

B

	Apparent K_d (μM)				
	ClpX	ClpXΔZBD	ZBD	ClpP	λO
TF	9.3 (1.8)	2.0 (0.5)	12.8 (2.9)	no interaction	3.8 (1.4)

Supplementary Figure 6. SPR binding analysis between TF, ClpX, and substrate.

(A) Sensorgrams of the binding of different proteins to full length TF, which was coupled on the chip; right panels show the binding curves as response units (RU) vs. the respective protein concentration at steady state and fitted to a one-site Langmuir binding model. For ClpP, injections did not generate any response over time even at high concentrations (30.7 µM) indicating no binding. For TF binding to ClpX, measurements at higher protein concentrations needed to reach saturation were not possible because of protein aggregation.

(B) Table listing the apparent K_d values obtained from A.

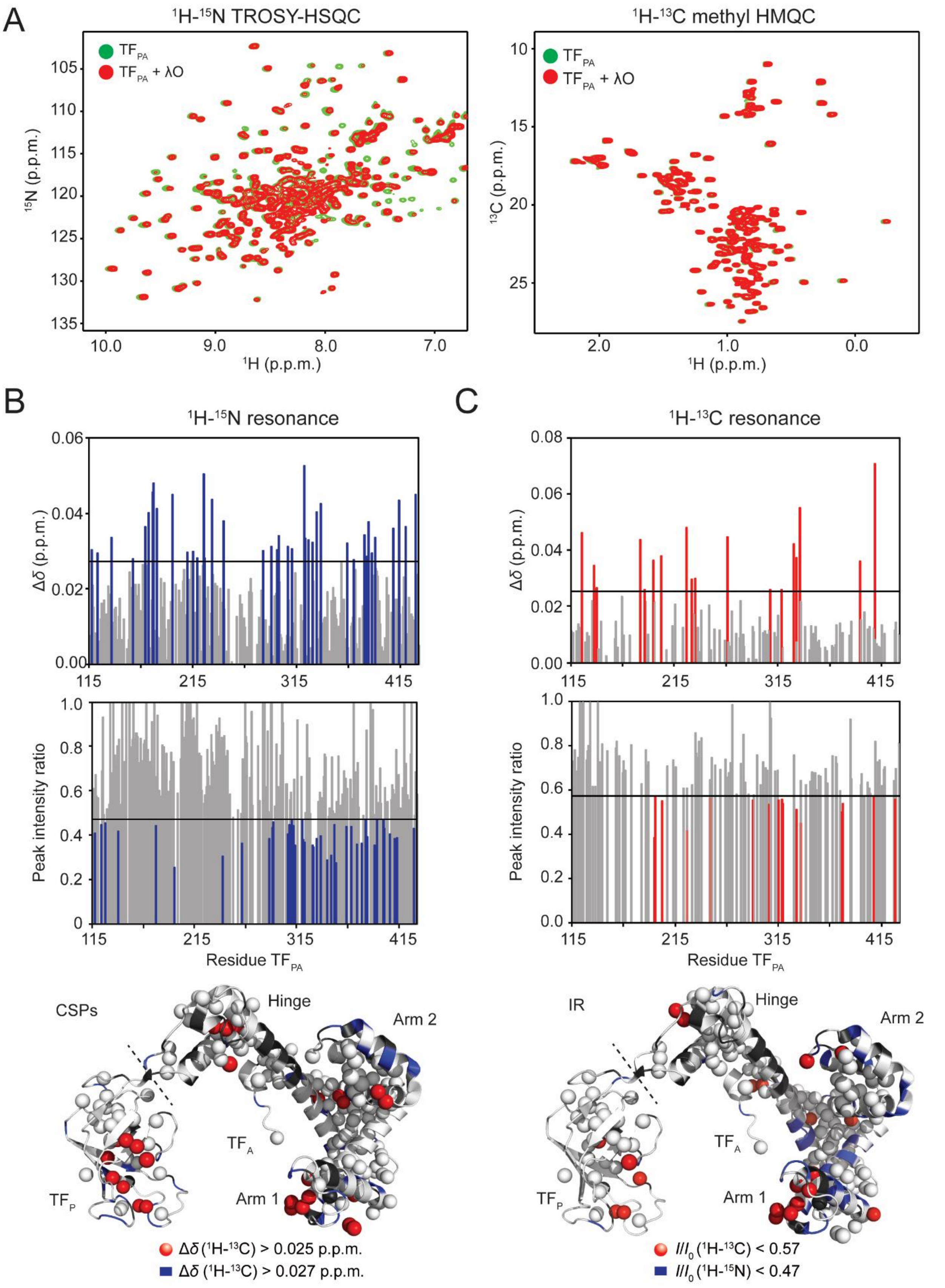

A
^{1}H-^{15}N TROSY-HSQC
TF$_{PA}$
TF$_{PA}$ + λO
^{15}N (p.p.m.)
^{1}H (p.p.m.)
^{1}H-^{13}C methyl HMQC
^{13}C (p.p.m.)
B
^{1}H-^{15}N resonance
C
^{1}H-^{13}C resonance
Δδ (p.p.m.)
Peak intensity ratio
Residue TF$_{PA}$
CSPs
IR
Hinge
Arm 2
Arm 1
TF$_A$
TF$_P$
Δδ (^{1}H-^{13}C) > 0.025 p.p.m.
Δδ (^{1}H-^{13}C) > 0.027 p.p.m.
I/I_0 (^{1}H-^{13}C) < 0.57
I/I_0 (^{1}H-^{15}N) < 0.47

Supplementary Figure 7. Mapping the interaction between TF_{PA} and λO by NMR.

(A) Overlay of the ^{1}H-^{15}N TROSY-HSQC (left panel) and ^{1}H-^{13}C HMQC (right panel) spectra of TF_{PA} in the absence (green) and presence (red) of λO.

(B, C) Analysis of the chemical shift change (top panels) and peak intensity change (middle panels) of the ^{1}H-^{15}N backbone amide (B) and ^{1}H-^{13}C methyl (C) resonances of TF_{PA} upon addition of λO. Bottom panels show mapping of the perturbations on the structure of TF (PDB code: 1W26)[32]. Dotted line indicates the domain boundary between TF_{A} and TF_{P}. The backbone structure is represented as the ribbon model and the methyl groups of Ile, Leu, Val, Met, and Ala in TF_{PA} are represented as spheres. The methyl groups and backbone of the residues indicating significant changes are colored red and blue, respectively. Other residues are colored in gray.

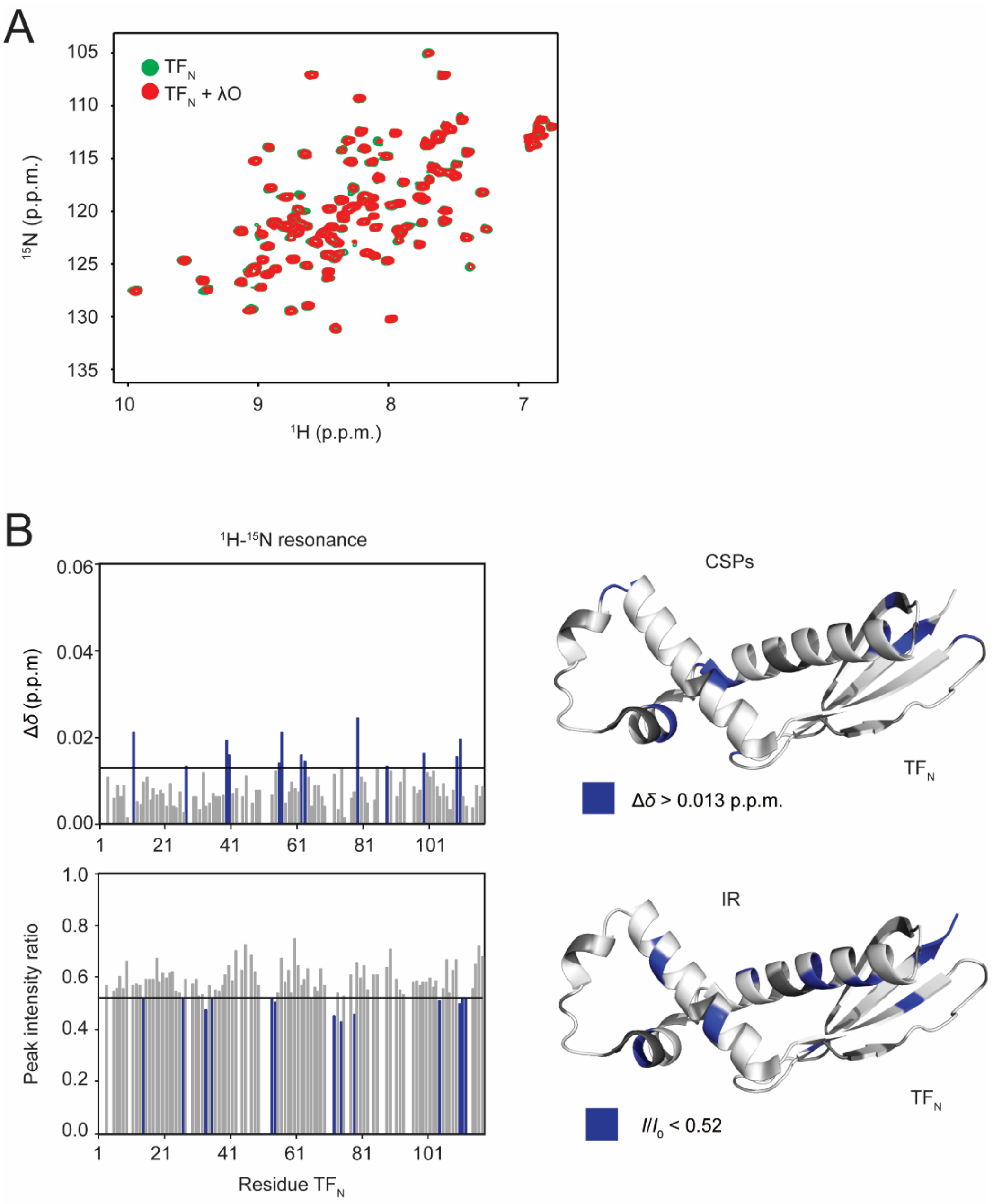
A
TF$_N$
TF$_N$ + λO
^{15}N (p.p.m.)
^{1}H (p.p.m.)
B
^{1}H-^{15}N resonance
Δδ (p.p.m)
CSPs
TF$_N$
Δδ > 0.013 p.p.m.
Peak intensity ratio
IR
TF$_N$
I/I_0 < 0.52
Residue TF$_N$

Supplementary Figure 8. Mapping the interaction between TF_N and λO by NMR.

(A) Overlay of the ^1H-^{15}N TROSY spectra of TF_N in the absence (green) and presence (red) of λO. (B) Analysis of the chemical shift change (upper left panel) and peak intensity change (lower left panel) of the ^1H-^{15}N backbone amide resonances of TF_N upon addition of λO. The right panels show mapping of the perturbations on the structure of TF (PDB code: 1W26)[32]. Residues indicating larger changes than cutoffs are colored blue, otherwise they are colored gray.

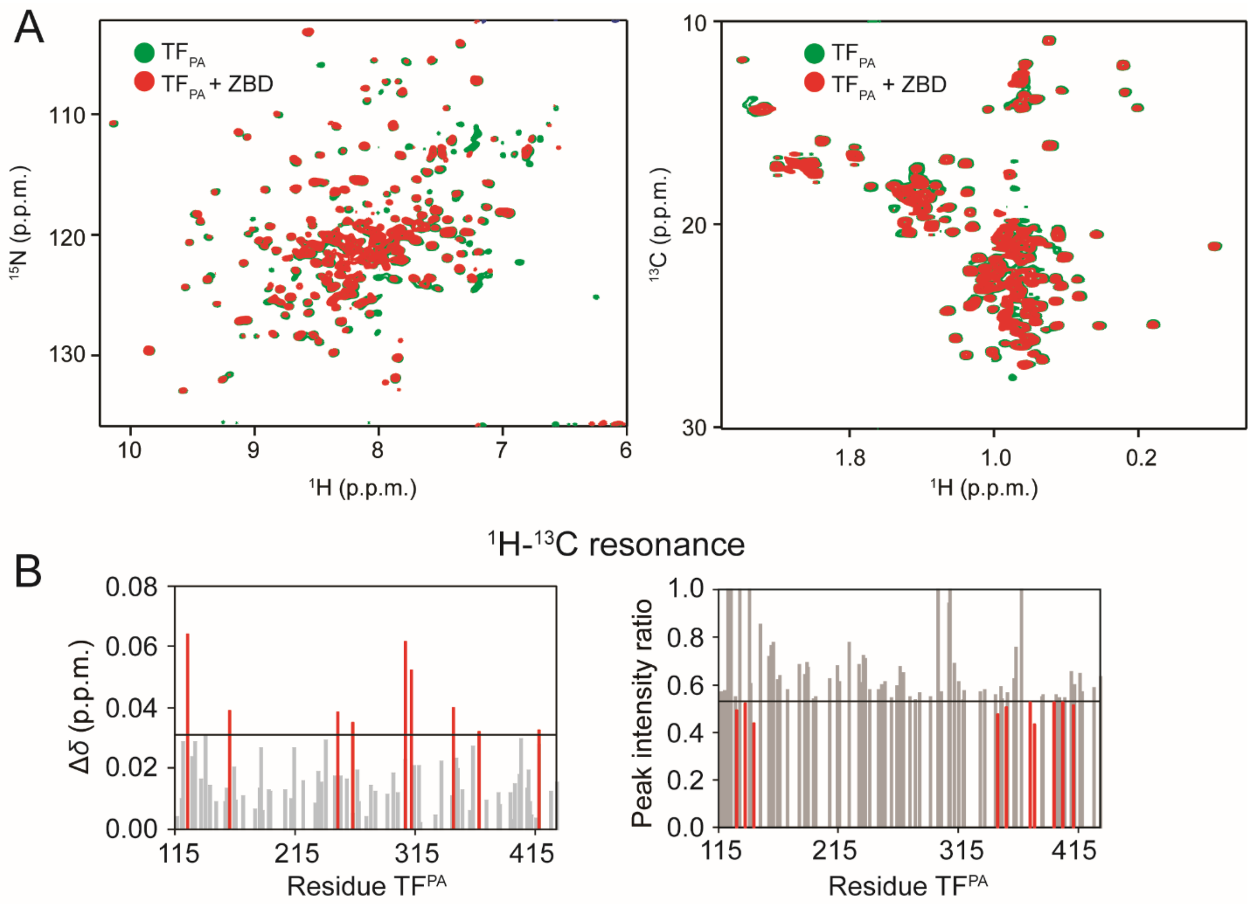
A
TF$_{PA}$
TF$_{PA}$ + ZBD
^{15}N (p.p.m.)
110
120
130
10
9
8
7
6
^{1}H (p.p.m.)
^{13}C (p.p.m.)
10
20
30
1.8
1.0
0.2
^{1}H-^{13}C resonance
B
Δδ (p.p.m.)
0.08
0.06
0.04
0.02
0.00
115
215
315
415
Residue TF^{PA}
Peak intensity ratio
1.0
0.8
0.6
0.4
0.2
0.0

Supplementary Figure 9. Mapping the interaction between TF_{PA} and ZBD by NMR.

(A) Overlay of the ^{1}H-^{15}N TROSY-HSQC (left panel) and ^{1}H-^{13}C HMQC (right panel) spectra of TF_{PA} in the absence (green) and presence (red) of ZBD.

(B) Analysis of the chemical shift change (left panel) and peak intensity change (right panel) of the ^{1}H-^{13}C methyl resonances of TF_{PA} upon addition of ZBD. Analysis for ^{1}H-^{15}N backbone amide CSPs and IRs are shown in Figure 4A.

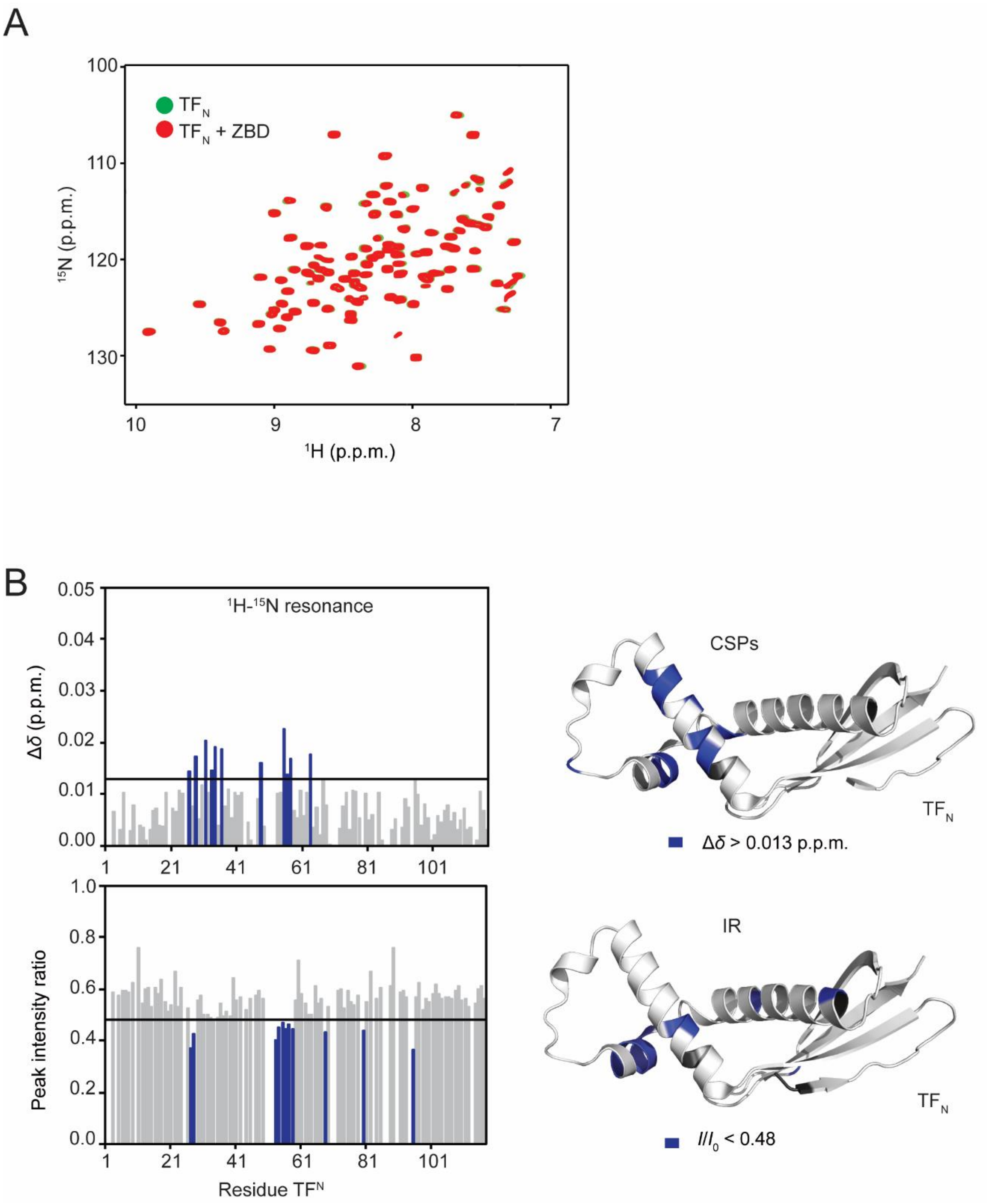
A
100
TF_N
TF_N + ZBD
110
15N (p.p.m.)
120
130
10
9
8
7
1H (p.p.m.)
B
0.05
1H-15N resonance
0.04
Δδ (p.p.m.)
0.03
0.02
0.01
0.00
1
21
41
61
81
101
CSPs
TF_N
Δδ > 0.013 p.p.m.
1.0
0.8
Peak intensity ratio
0.6
0.4
0.2
0.0
Residue TF^N
IR
TF_N
I/I_0 < 0.48

Supplementary Figure 10. Mapping the interaction between TF_N and ZBD by NMR.

(A) Overlay of the ^{1}H-^{15}N TROSY spectra of TF_N in the absence (green) and presence (red) of ZBD.

(B) Analysis of the chemical shift change (upper left panel) and peak intensity change (lower left panel) of the ^{1}H-^{15}N backbone amide resonances of TF_N upon addition of ZBD. The right panels show mapping of the perturbations on the structure of TF (PDB code: 1W26)[32]. The residues indicating significant changes are colored blue, others are colored gray.

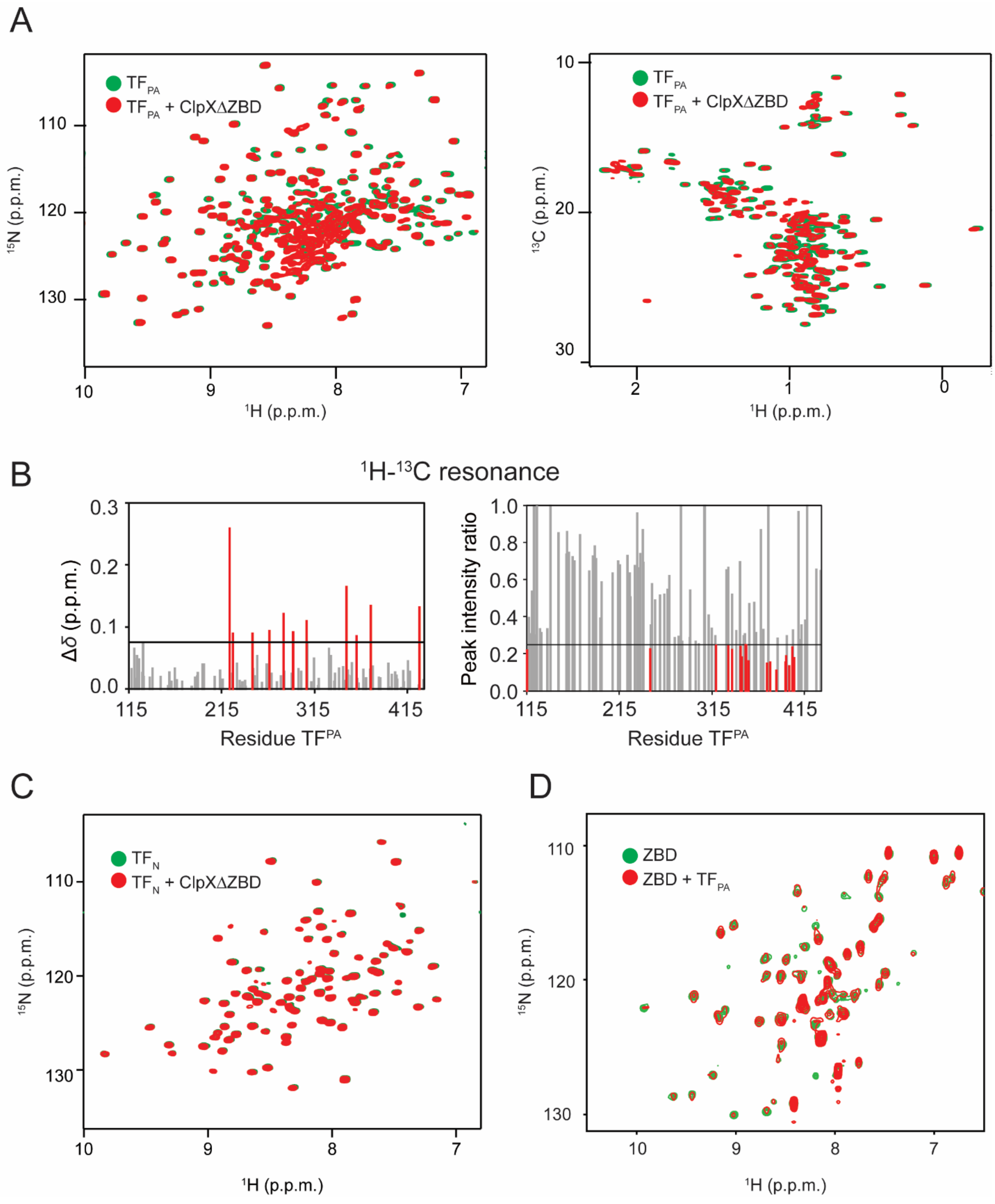
A
TF_PA
TF_PA + ClpXΔZBD
15N (p.p.m.)
1H (p.p.m.)
13C (p.p.m.)
B
1H-13C resonance
Δδ (p.p.m.)
Residue TF^PA
Peak intensity ratio
Residue TF^PA
C
TF_N
TF_N + ClpXΔZBD
15N (p.p.m.)
1H (p.p.m.)
D
ZBD
ZBD + TF_PA
15N (p.p.m.)
1H (p.p.m.)

Supplementary Figure 11. Mapping the interactions between TF_{PA}/ TF_N and ClpXΔZBD/ZBD by NMR.

(A) Overlay of the ^{1}H-^{15}N TROSY-HSQC (left panel) and ^{1}H-^{13}C HMQC (right panel) spectra of TF_{PA} in the absence (green) and presence (red) of ClpXΔZBD.

(B) Analysis of the chemical shift change (left panel) and peak intensity change (right panel) of ^{1}H-^{13}C methyl resonances of TF_{PA} upon addition of ClpXΔZBD. The analysis for ^{1}H-^{15}N backbone amide are summarized in Figure 4B.

(C) Overlay of the ^{1}H-^{15}N TROSY-HSQC spectra of TF_N in the absence (green) and presence (red) of ClpXΔZBD.

(D) Overlay of the ^{1}H-^{15}N TROSY-HSQC spectra of ZBD in the absence (green) and presence (red) of TF_{PA}.

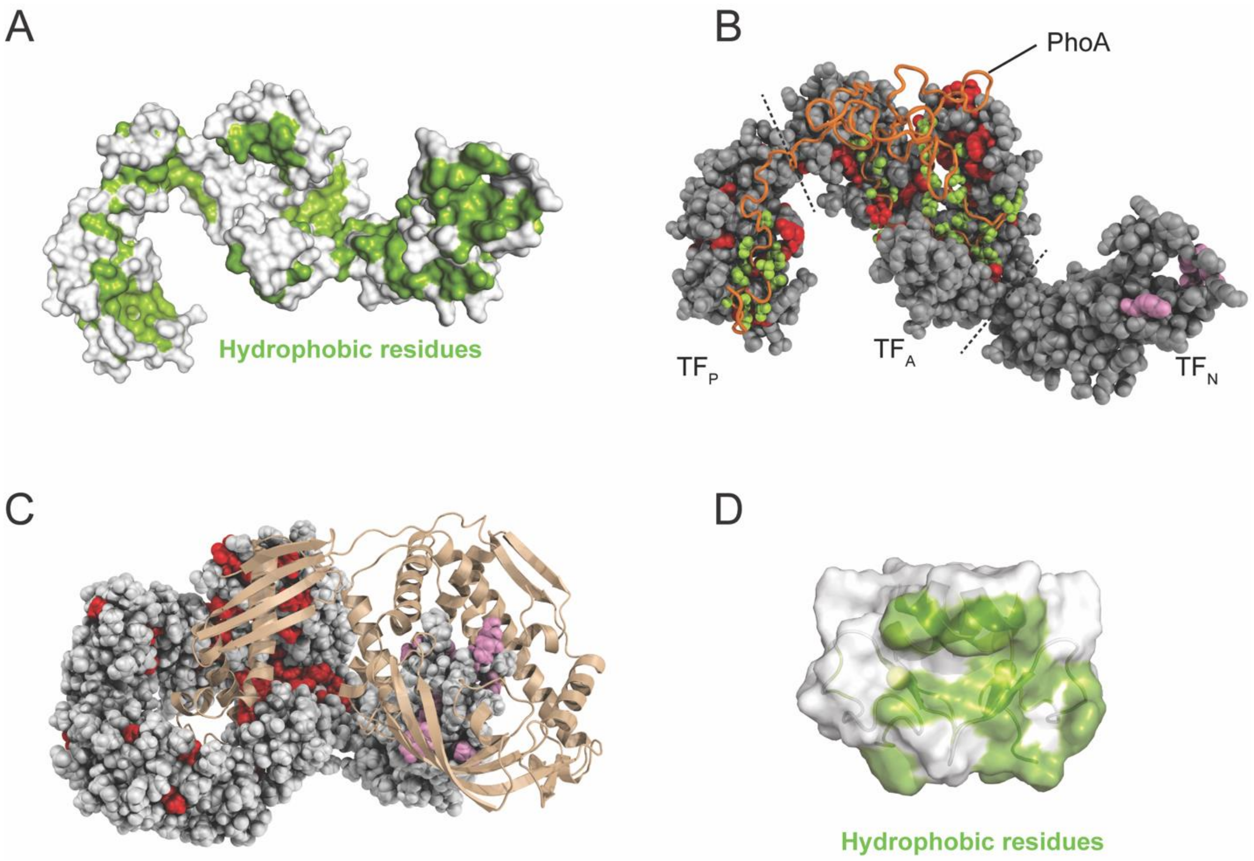
A
Hydrophobic residues
B
PhoA
TF_P
TF_A
TF_N
C
D
Hydrophobic residues

Supplementary Figure 12. Overlap of the ClpX-binding sites, substrate-binding sites, and dimer interaction sites in TF.

(A) Mapping of hydrophobic residues onto the structure of TF. The hydrophobic amino acid residues are colored green.

(B) Mapping of the amino acid residues of TF involved in the recognition of ClpX to the structure of TF in complex with the unfolded substrate protein PhoA (PDB code: 2MLY)[31]. Residues of TF_{PA} that showed significant perturbations upon addition of ZBD or ClpXΔZBD (Figures 4A,B) are colored red. Residues of TF_{N} that showed perturbations upon addition of ClpXΔZBD (Figurer 4C) are colored pink. TF is shown as white space-filling model and PhoA is shown as orange ribbon. The residues of PhoA recognized by TF are shown as green ball-stick model. Domain boundaries are indicated by dotted lines.

(C) Mapping of the amino acid residues of TF involved in the recognition of ClpX to the structure of TF dimer (PDB code: 6D6S)[8]. One subunit of the dimer is shown as ribbon model in brown, and the other subunit is shown as a space-filling model in gray in which the perturbed resonances are colored as in the panel B.

(D) Mapping of the hydrophobic residues on the structure of ClpX ZBD dimer (PDB code: 1OVX)[72]. The hydrophobic amino acid residues are colored green and zinc ions are shown as spheres.

A

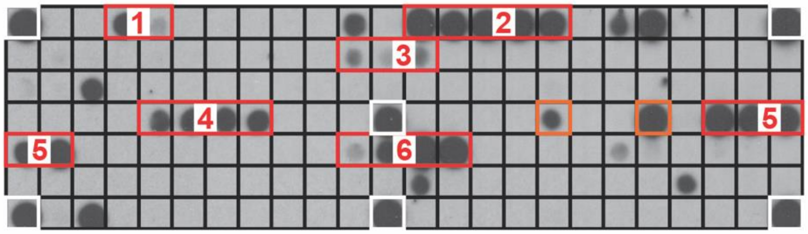

B

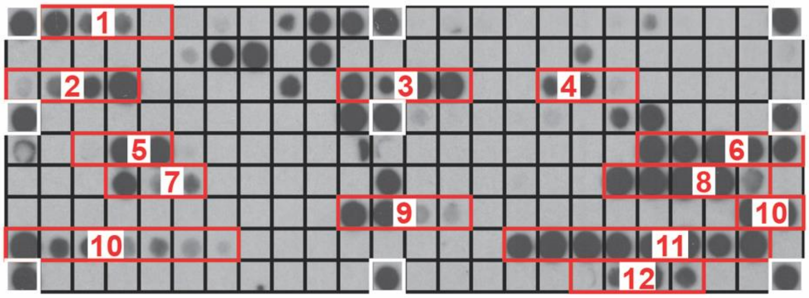

C **Consensus motifs of TF peptides bound by ClpXΔZBD**

Motif 1 (residues 7-21): T T Q **G L G R R V T I T** I A A

Motif 2a (residues 37-48): K K V R I D **G F R K G K**
Motif 2b (residues 43-57): **G F R K G K** V P M N I V A Q R

Motif 3 (residues 100-111): G E D **F T Y S V E** F E V

Motif 4 (residues 229-237): K A A **K F A** I N L

Motif 5a (residues 265-267): **G L R** (only found when "GLR" is at N- or C-end)
Motif 5b (residues 274-288): M E R E L K **S A I R N R** V K S
Motif 5c (residues 274-288): M E R E L K **S A I R N R** V K S

Motif 6 (residues 316-324): R Q A **A Q R** F G G

D **Consensus motifs of ClpXΔZBD peptides bound by TF**

Motif 1 (residues 3-14): D K **R K D G S G K L** L Y
Motif 2 (residues 95-104): H Y **K R L R N G** D T
Motif 3 (residues 115-124): I L **L I G P T G** S G
Motif 4 (residues 125-136): K T **L L A E T L A R** L L
Motif 5 (residues 187-198): D K **I S R K S D N P** S I
Motif 6 (residues 225-232): Q G **G R K H** P Q
Motif 7 (residues 237-248): Q V **D T S K I L F I** C G
Motif 8 (residues 271-278): G A **T V K A** K S
Motif 9 (residues 301-310): I P **E F I G R L** P V
Motif 10a+10b+10c+10d (residues 327-342): K E P K **N A L T K Q Y Q** A L F N
Motif 11a+11b+11c (residues 361-374): K A M A **R K T G A R** G L R S
Motif 12 (residues 407-416): S K **P L L I Y G** K P

SupplementaryFigure 13. TF and ClpXΔZBD consensus motifs obtained by peptide array analysis. Miniaturized libraries of overlapping 12mer peptides were synthesized by walking through the amino acid sequence of *E. coli* TF or *E. coli* ClpX, advancing three (TF) or two amino acids (ClpX) at a time. Overlapping peptides were grouped into consensus motifs. For each consensus motif, the common region (consensus) is underlined. Amino acids belonging to one or more consensus motif are highlighted in bold if a complete set of overlapping peptides featuring this amino acid were found. Peptides that miss one or both neighbors and do not form a consensus motif were eliminated. Spots belonging to the same consensus motif are boxed in red. Motifs are numbered sequentially. Positive binding controls are boxed in white.

(A) Overlapping peptides bound by ClpXΔZBD. Motif 5a is boxed in orange.

(B) Overlapping peptides bound by TF.

(C,D) Sequences of consensus motifs of TF and ClpXΔZBD respectively.

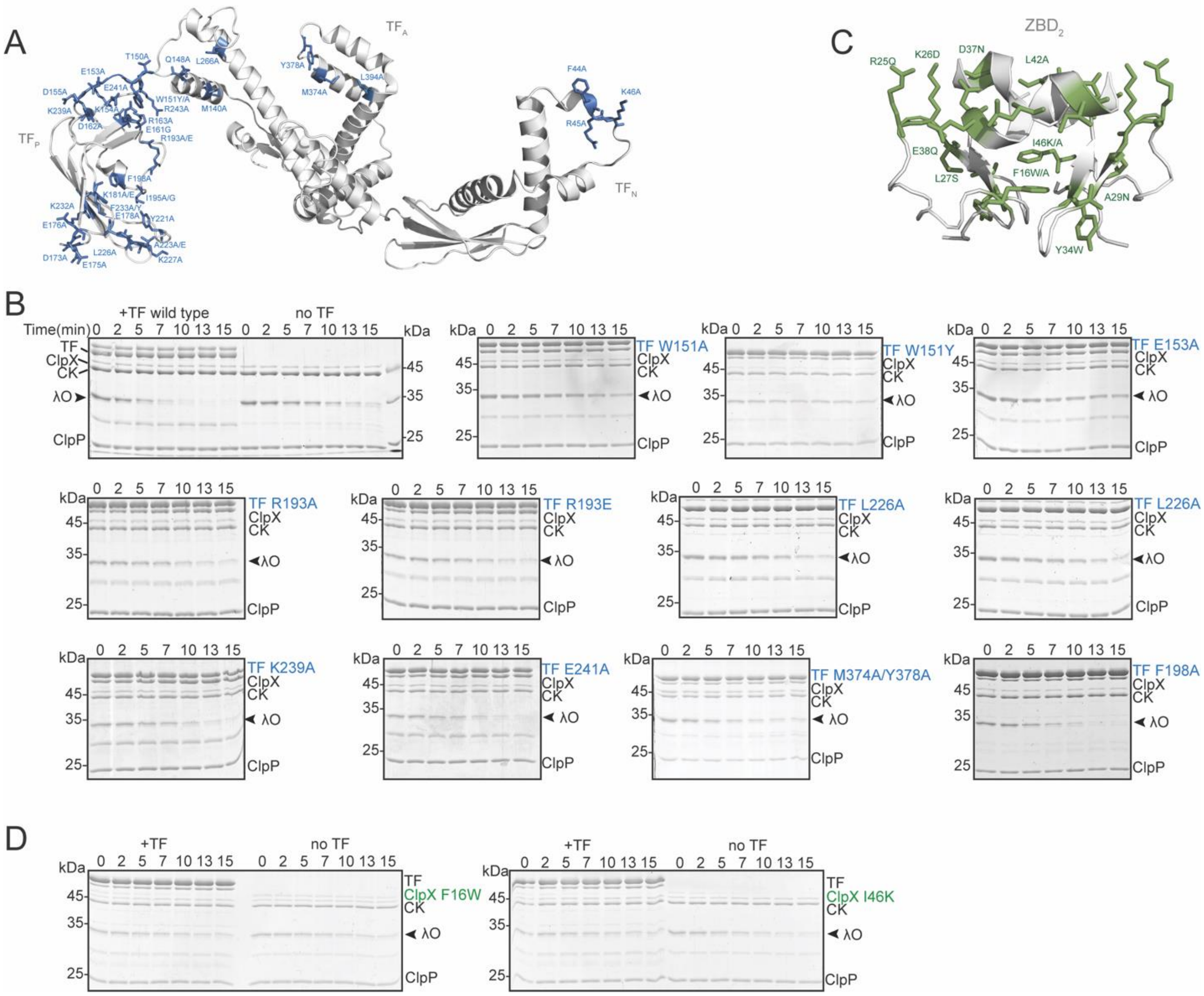
A
TF$_P$
TF$_A$
TF$_N$
C
ZBD$_2$
R25Q
K26D
D37N
L42A
E38Q
I46K/A
L27S
F16W/A
A29N
Y34W
B
+TF wild type
no TF
Time(min) 0 2 5 7 10 13 15
kDa
TF
ClpX
CK
λO
ClpP
TF W151A
TF W151Y
TF E153A
TF R193A
TF R193E
TF L226A
TF K239A
TF E241A
TF M374A/Y378A
TF F198A
D
+TF
no TF
ClpX F16W
ClpX I46K

Supplementary Figure 14. Mutagenesis of TF and ClpX ZBD.

(A and C) Ribbon representation of the TF structure (PDB:1W26) and ZBD_2 (PDB: 1OVX) highlighting the residues chosen for mutagenesis screen in *in vitro* degradation assays of λO. Residues mutated are shown in both ZBD protomers. Labeled residues face the reader.

(B and D) Representative SDS-PAGE gels of the degradation of λO as a function of time in the absence and presence of WT TF, eleven TF mutants (blue), WT ClpX, and two ClpX ZBD mutants (green). At least three replicate reactions were performed in B and D. Source data are provided as a Source Data file.

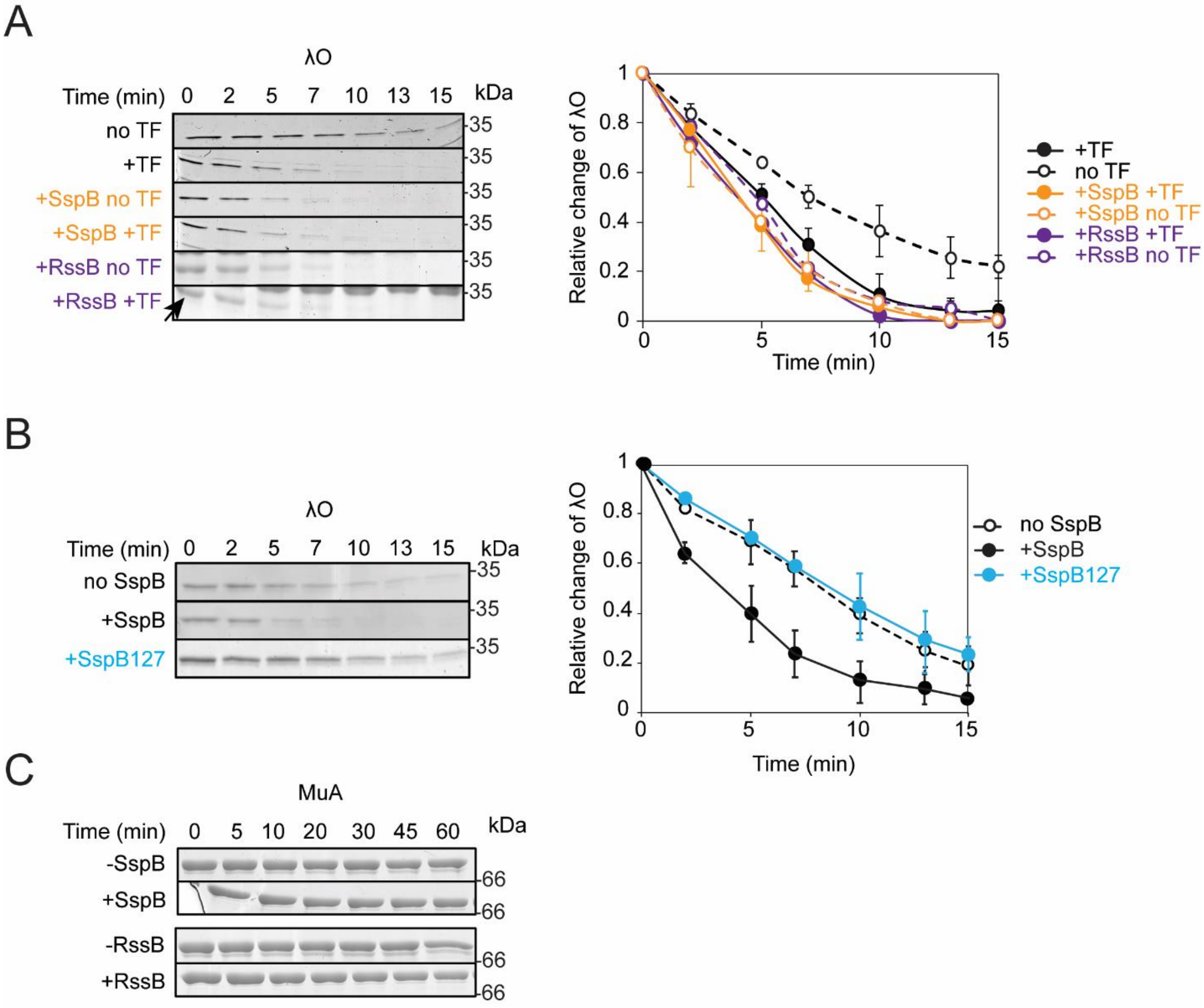
A
λO
Time (min) 0 2 5 7 10 13 15 kDa
no TF
+TF
+SspB no TF
+SspB +TF
+RssB no TF
+RssB +TF
35
Relative change of λO
Time (min)
+TF
no TF
+SspB +TF
+SspB no TF
+RssB +TF
+RssB no TF
B
λO
Time (min) 0 2 5 7 10 13 15 kDa
no SspB
+SspB
+SspB127
35
Relative change of λO
Time (min)
no SspB
+SspB
+SspB127
C
MuA
Time (min) 0 5 10 20 30 45 60 kDa
-SspB
+SspB
-RssB
+RssB
66

Supplementary Figure 15. SspB and RssB enhance ClpXP-dependent degradation of λO.

(A) Degradation of λO in the presence and absence of SspB, RssB, TF, SspB with TF, and RssB with TF. SDS-PAGE gels are shown on the left. Curves on the right represent the average value and standard deviations of at least three independent reactions.

(B) Degradation of λO in the absence and presence of SspB and SspB127 truncation mutant that is unable to bind to ClpX. Error bars represent the standard deviations from at least three independent reactions. Source data are provided as a Source Data file.

(C) Degradation assay of MuA in the absence and presence of SspB and RssB.

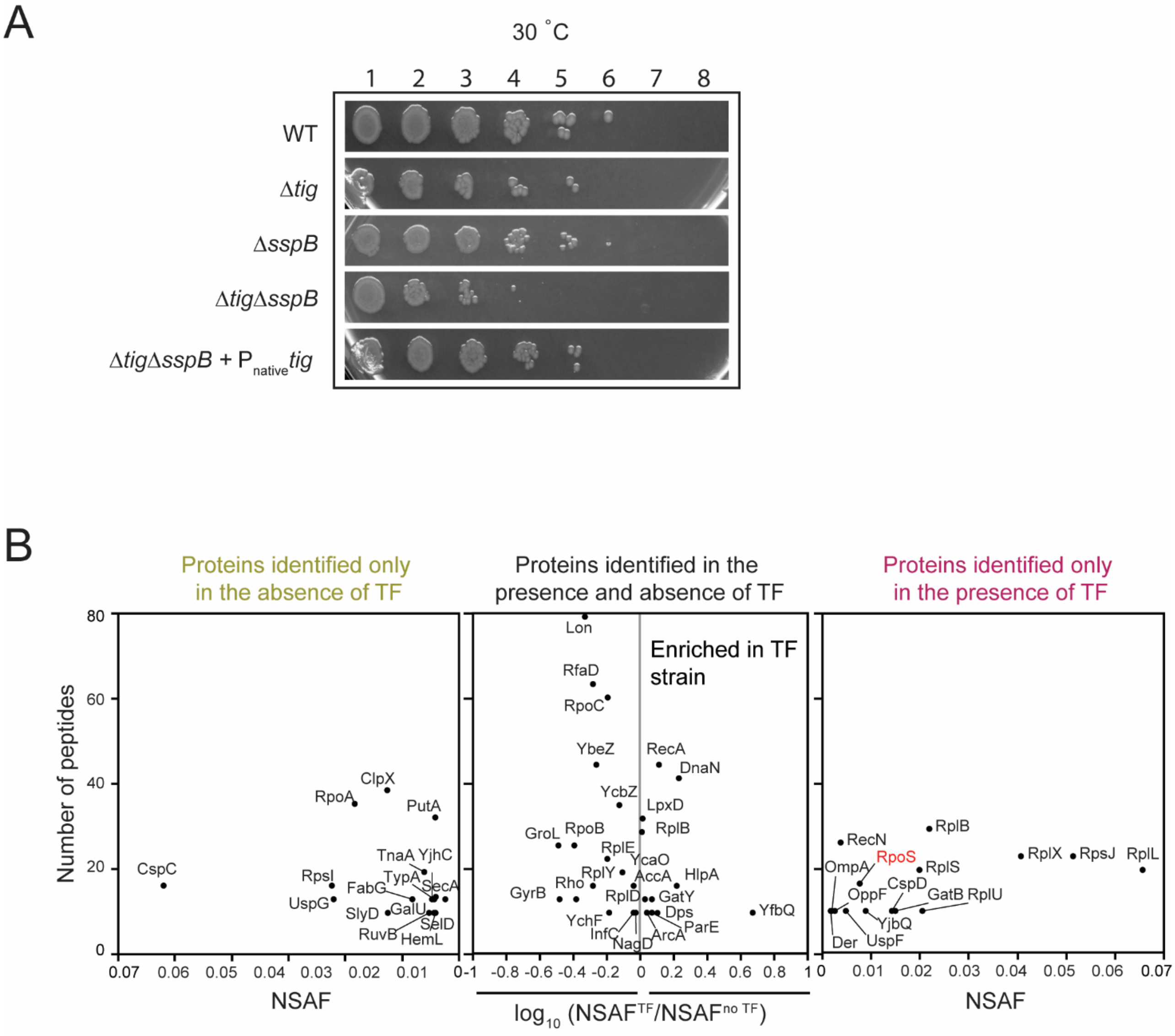
A
30 ˚C
1 2 3 4 5 6 7 8
WT
Δtig
ΔsspB
ΔtigΔsspB
ΔtigΔsspB + P native tig
B
Proteins identified only in the absence of TF
Proteins identified in the presence and absence of TF
Proteins identified only in the presence of TF
Number of peptides
Enriched in TF strain
NSAF
log10 (NSAF TF/NSAF no TF)
CspC
RpoA
ClpX
PutA
TnaA
YjhC
RpsI
TypA
FabG
SecA
UspG
SlyD
GalU
SelD
RuvB
HemL
Lon
RfaD
RpoC
YbeZ
RecA
DnaN
YcbZ
LpxD
RplB
GroL
RpoB
RplE
YcaO
RplY
AccA
HlpA
GyrB
Rho
RplD
GatY
Dps
YfbQ
YchF
InfC
NagD
ArcA
ParE
RecN
RplB
OmpA
RpoS
RplS
RplX
RpsJ
RplL
CspD
OppF
GatB
RplU
YjbQ
Der
UspF

Supplementary Figure 16. Effect of TF and SspB on *E.* coli MC4100 fitness and trapping substrates of ClpXP in a TF-dependent manner.

(A) Growth of WT MC4100, Δ*tig*, Δ*sspB*, Δ*tig*Δ*sspB* and Δ*tig*Δ*sspB* expressing TF from its native promoter using the p11 plasmid were grown overnight and serially diluted 10-fold, and spotted on LB agar plates then incubated for 20 hrs at 30 °C.

(B) Plots of peptide counts vs. NSAF (normalized spectral abundance factor)[71] of proteins captured by $ClpP^{trap}$ and identified by MS in cell lines containing (Δ*clpP*Δ*clpA*Δ*smpB*) or lacking TF (Δ*clpP*Δ*clpA*Δ*smpB*Δ*tig*). The ClpP (bait) protein is not shown. Source data are provided as a Source Data file.

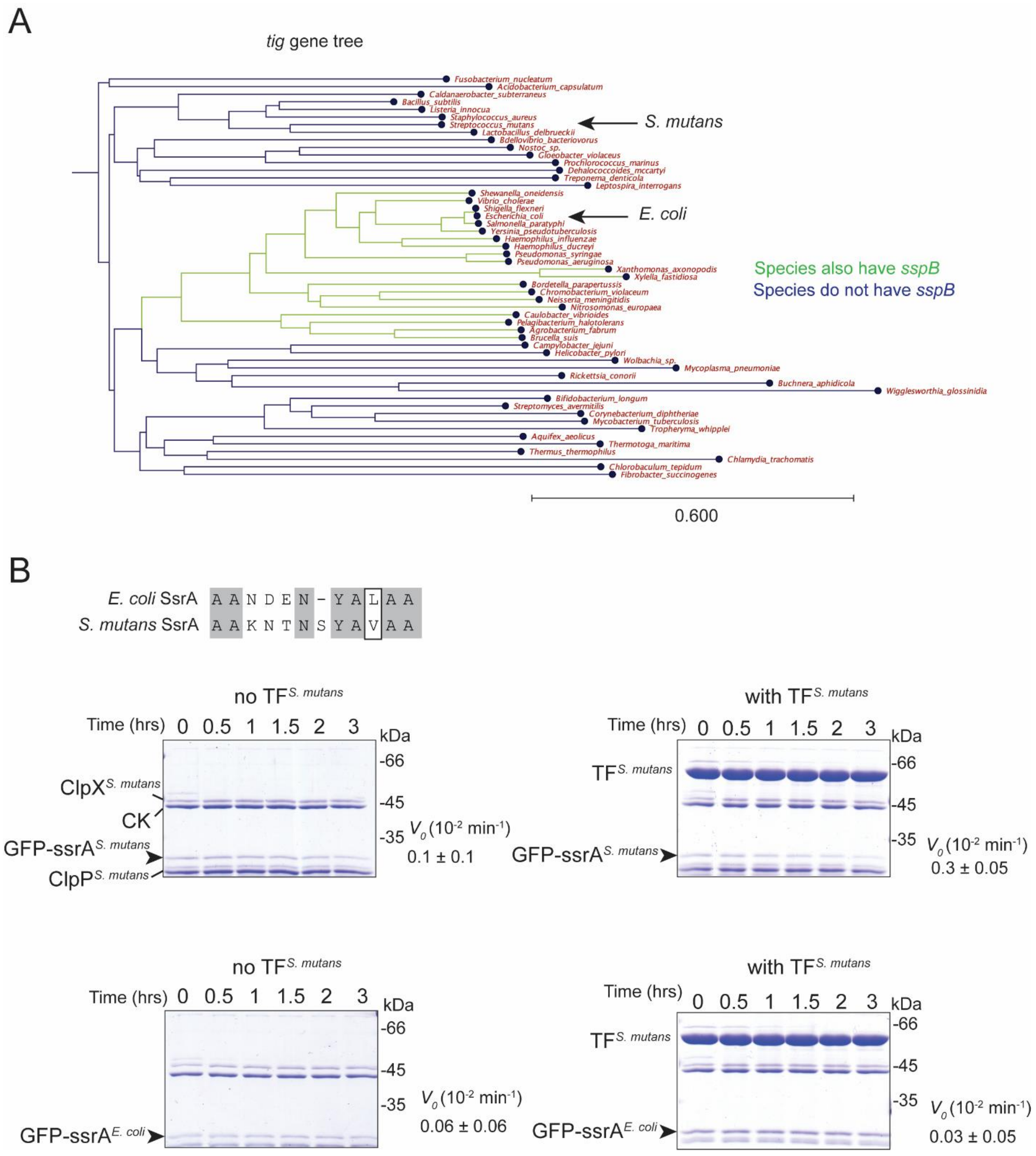
A
tig gene tree
Fusobacterium_nucleatum
Acidobacterium_capsulatum
Caldanaerobacter_subterraneus
Bacillus_subtilis
Listeria_innocua
Staphylococcus_aureus
Streptococcus_mutans
Lactobacillus_delbrueckii
Bdellovibrio_bacteriovorus
Nostoc_sp.
Gloeobacter_violaceus
Prochlorococcus_marinus
Dehalococcoides_mccartyi
Treponema_denticola
Leptospira_interrogans
S. mutans
Shewanella_oneidensis
Vibrio_cholerae
Shigella_flexneri
Escherichia_coli
Salmonella_paratyphi
Yersinia_pseudotuberculosis
Haemophilus_influenzae
Haemophilus_ducreyi
Pseudomonas_syringae
Pseudomonas_aeruginosa
Xanthomonas_axonopodis
Xylella_fastidiosa
E. coli
Bordetella_parapertussis
Chromobacterium_violaceum
Neisseria_meningitidis
Nitrosomonas_europaea
Caulobacter_vibrioides
Pelagibacterium_halotolerans
Agrobacterium_fabrum
Brucella_suis
Campylobacter_jejuni
Helicobacter_pylori
Wolbachia_sp.
Mycoplasma_pneumoniae
Rickettsia_conorii
Buchnera_aphidicola
Wigglesworthia_glossinidia
Bifidobacterium_longum
Streptomyces_avermitilis
Corynebacterium_diphtheriae
Mycobacterium_tuberculosis
Tropheryma_whipplei
Aquifex_aeolicus
Thermotoga_maritima
Thermus_thermophilus
Chlamydia_trachomatis
Chlorobaculum_tepidum
Fibrobacter_succinogenes
Species also have sspB
Species do not have sspB
0.600
B
E. coli SsrA A A N D E N - Y A L A A
S. mutans SsrA A A K N T N S Y A V A A
no TF S. mutans
Time (hrs) 0 0.5 1 1.5 2 3
kDa
-66
-45
-35
ClpX S. mutans
CK
GFP-ssrA S. mutans
ClpP S. mutans
V0 (10-2 min-1)
0.1 ± 0.1
with TF S. mutans
Time (hrs) 0 0.5 1 1.5 2 3
kDa
-66
-45
-35
TF S. mutans
GFP-ssrA S. mutans
V0 (10-2 min-1)
0.3 ± 0.05
no TF S. mutans
Time (hrs) 0 0.5 1 1.5 2 3
kDa
-66
-45
-35
GFP-ssrA E. coli
V0 (10-2 min-1)
0.06 ± 0.06
with TF S. mutans
Time (hrs) 0 0.5 1 1.5 2 3
kDa
-66
-45
-35
TF S. mutans
GFP-ssrA E. coli
V0 (10-2 min-1)
0.03 ± 0.05

Supplementary Figure 17. TF acts as an adaptor protein in *Streptococcus mutans*.

(A) Phylogenetic tree of the *tig* gene in bacteria made using the CLC Genomics Workbench version 11.0.1 (Aarhus, Denmark). Bacterial species that also contain *sspB* are highlighted. Tree scale represents the number of substitutions per site.

(B) Sequence alignment of *E. coli* and *S. mutans* SsrA tags with identical residues highlighted in grey and the key L/V residues boxed. The upper gels show the *in vitro* degradation of the GFP-ssrA tag from *S. mutans* using *S. mutans* ClpXP in the absence and presence of *S. mutans* TF. The lower gels show the same assay but using GFP-ssrA with the *E. coli* tag. V_0 refers to initial rate of disappearance of GFP-ssrA$^{S.\ mutans}$. Standard deviations were calculated from three independent experiments. Source data are provided as a Source Data file.

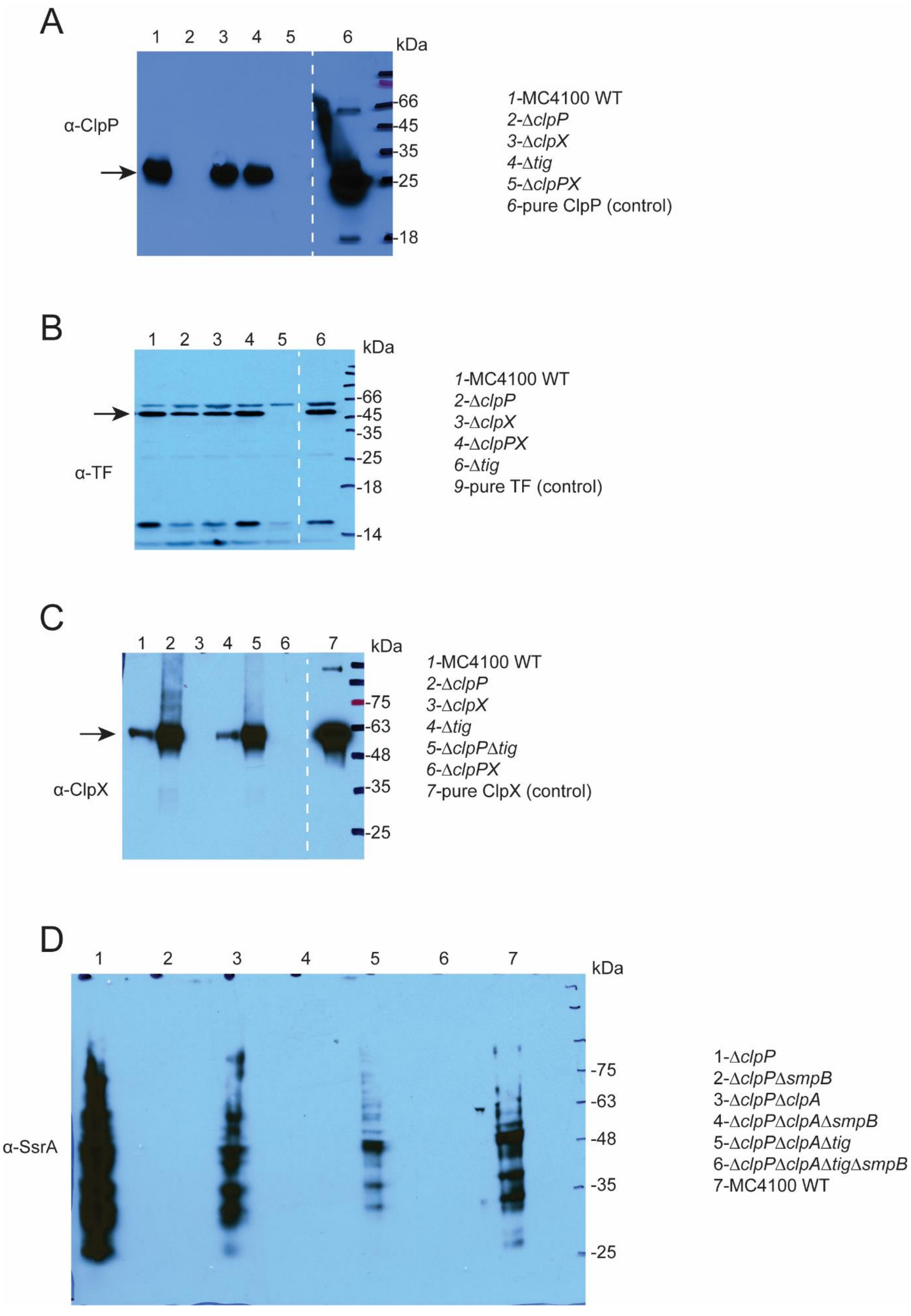
A
α-ClpP
1 2 3 4 5 6 kDa
-66
-45
-35
-25
-18
1-MC4100 WT
2-ΔclpP
3-ΔclpX
4-Δtig
5-ΔclpPX
6-pure ClpP (control)
B
α-TF
1 2 3 4 5 6 kDa
-66
-45
-35
-25
-18
-14
1-MC4100 WT
2-ΔclpP
3-ΔclpX
4-ΔclpPX
6-Δtig
9-pure TF (control)
C
α-ClpX
1 2 3 4 5 6 7 kDa
-75
-63
-48
-35
-25
1-MC4100 WT
2-ΔclpP
3-ΔclpX
4-Δtig
5-ΔclpPΔtig
6-ΔclpPX
7-pure ClpX (control)
D
α-SsrA
1 2 3 4 5 6 7 kDa
-75
-63
-48
-35
-25
1-ΔclpP
2-ΔclpPΔsmpB
3-ΔclpPΔclpA
4-ΔclpPΔclpAΔsmpB
5-ΔclpPΔclpAΔtig
6-ΔclpPΔclpAΔtigΔsmpB
7-MC4100 WT

Supplementary Figure 18. Immunoblot staining for strain validation.

(A-D) Blots are shown for the indicated strains using the respective antibodies. Blots with dashed lines are from the same gel.

Supplementary Table 1: Strains used in this study

Name	Background	Genotype	References
Wild type MC4100	K12	F⁻ *araD139 Δ(argF-lac)U169 rpsL150* (Str^R)*relA1 flbB5301 deoC1 ptsF25 rbsR*	(1)
SG1146	BL21(DE3)	*clpP*::Cm *flhDC*::*Km*	(2)
Δ*clpP*	MC4100	clpP::*cat*	This study
Δ*tig*	MC4100	*tig*::*cat*	This study
Δ*clpX*	MC4100	*clpX*::*cat*	This study
Δ*clpPX*	MC4100	no marker	This study
Δ*sspB*	MC4100	no marker	This study
Δ*tig*Δ*sspB*	MC4100	no marker	This study
Δ*smpB*	MC4100	no marker	This study
Δ*clpP*Δ*clpA*	MC4100	*clpA*::*kan*	This study
Δ*clpP*Δ*clpA*Δ*tig*	MC4100	*clpA*::*kan*	This study
Δ*clpP*Δ*clpA*Δ*smpB*	MC4100	*smpB*::*kan*	This study
Δ*clpP*Δ*clpA*Δ*tig*Δ*smpB*	MC4100	*smpB*::*kan*	This study
Δ*htpG*	BW25113	*htpG*::*kan*	(3)

Supplementary Table 2: Primers used in this study

ClpP_S111A_SPA_F_Xba1	TCAGTCTAGAATGGCGCTGGTGCCGATGG
ClpP_NdeI_F	GATCCATATGGCGCTGGTGCCGATGGTC
ClpP_BamHI_F	GATCGGATCCGCGCTGGTGCCGATGGTC
ClpP-SPA_link_F2	GAATACGGTCTGGTCGATTCGATTCTGACCCATCG TAATTCCATGGAAAAGAGAAGATG
ClpP-SPA_link_F	TTCTGACCCATCGTAATTCCATGGAAAAGAGAAG AT
ClpP-SPA_link_R	TCTTCTCTTTTCCATGGAATTACGATGGGTCAGAA T
ClpP-SPA_link_R2	GGCTATGAAATTCTTTTTCCATCTTCTCTTTTCCAT GGAATTACGATGGGTCAGAATCG
RssB_BamHI_F	ATCGGGATCCATGACGCAGCCATTGGTCGGAAAA CAG
RssB_HindIII_R	GACTGCGCTTGATGTTGTCTGCAGAATGAAAGCTT ATCG
Rpos_HindIII_R	CGATAAGCTTTTTTACTCGCGGAACAGCGC
Rpos_EcoRI_F	ATCGGAATTCAAATGAGTCAGAATACGCT
R_S7	TACCTGATAAGTAGAACCACC
F_S7	GGTGGTTCTACTTATCAGGTA
LO_N_motif_S7_F_NdeI	ACGTCCCATGGCAAATACAGCAAAAATACTCAAC TTCGGCAGAATGCCACGTCGTCGCGTCATTGG
Tig_S.mutans_F_BamHI	CTGAGGA TCC ATGTCTACATCATTTGAAAACAAAGC
Tig_S.mutans_R_XhoI	TCGACTCGAGGATTATTTAACTTTAGCAGAATCAG TAATAACG
ClpX_S.mutans_F_BamHI	CTGAGGATCCATGGCTGGAAATAGAACCAATGAT G
ClpX_S.mutans_R_XhoI	TCGACTCGAGGATTAGGAAGCTGTCTCAAGAATA GGTTTGTCGG
ClpP_S.mutans_F_BamHI	CTGAGGATCCATGATTCCTGTAGTTATTGAACAAA CGAGCCG
ClpP_S.mutans_R_XhoI	TCGACTCGAGGATTATTTTAATTCATTATTTTCCAT GATTTCATCGATGAAGCC
R_smGFP_ssra_BamHI	GCGCGGATCCTTAGGCAGCTACTGCGTAAGAATT TGTATTTTTTGCTGCAGCGCT
F_Ehe1_GFP	CGCGTAGGCGCCATGAGTAAAGGAGAAGAACTTT TC
F2_tig_pro	GCTTTTCCAGTATGTTGCTAAAGAT
R2_tig_pro	CATCACCATATTTCAAGCATAACAA
F_TF_M374AY378AV384AF37 8A	CTGATCGAAGAGGCGGCTTCTGCGGCCGAAGATC CGAAAGAAGCTATCGAGGCC

R_TF_M374Y378V384F378	GTTTTTGCTGTAGGCCTCGATAGCTTCTTTCGGAT CTTCGGCCGCAGAAGCCGC
ClpA_F	GAATTTATCCACCACCTGAT
ClpA_R	TGTTCTGTGGATTTGGTTGC
F_tig_198A	GATCCCGGGCGCTGAAGACGGTATCAAAGGCCA
R_tig_F198A	TACCGTCTTCAGCGCCCGGGATCATACGACCCT
SmpB_F	GCGACCATCGCGCTTAACAA
SmpB_R	TACGTGCTTTATCCACCTGCC

SUPPLEMENTARY REFERENCES

1. Casadaban M J. Transposition and fusion of the lac genes to selected promoters in Escherichia coli using bacteriophage lambda and Mu. *J Mol Biol*, **104**, 4510-455 (1976).

2. Bakkouri El M, Rathore S, Calmettes C, Wernimont AK, Liu K, Sinha D, *et al*. Structural insights into the inactive subunit of the apicoplast-localized caseinolytic protease complex of Plasmodium falciparum. *J Biol Chem*, **288**, 1022–1031 (2013).

3. Baba T, *et al.* Construction of Escherichia coli K-12 in-frame, single-gene knockout mutants: the Keio collection. *Mol Syst Biol* **2**, 2006 0008 (2006).
