# Supplementary material for: Functional cooperativity between the trigger factor chaperone and the ClpXP proteolytic complex
Source: Nat Commun. 2021 Jan 12;12:281. doi: 10.1038/s41467-020-20553-x (PMC7804408; doi:10.1038/s41467-020-20553-x)
Supplement: Supplementary file 2 — Peer Review File [file 41467_2020_20553_MOESM2_ESM.pdf]

Reviewers' comments:

Reviewer #1 (Remarks to the Author):

The manuscript by Rizzolo et al investigates a functional cooperativity between the chaperone trigger factor (TF) and the ClpX protease. Based on the initial observation that the genes for TF and ClpX are organized in the same operon for many species, the work investigates with a series of biochemical, biophysical, structural and in cell experiments in how far the two proteins could interact physically with each other and which functional consequences this may have. The work consists of an impressively large collection of different assays and experiments, most of which point to the existence of some kind of interaction. The conclusion by the authors is that TF interacts with ClpX via multiple sites in a dynamic fashion. In general, the Figures are of high quality and the experiments rather well document (with some exceptions, see below).

When investigating this question about a functional coupling between TF and ClpX, one should keep in mind that it is per se not surprising that TF and ClpX can exhibit cooperative effects, since they both are well known to interact with unfolded and partially folded proteins and peptides. The crucial point is therefore whether any observed effect is necessarily based on a direct interaction between TF and ClpX or rather an indirect effect, such as mediated through their respective interactions with the clients that can lead to sequestering / protection / competition effects.

Here is a list of technical and interpretational issues that should be addressed.

1.) Figure S6A shows the final results of a either TF or ClpX binding to a peptide binding array of the respective other protein. For this experiment, the underlying data needs to be shown in suitable format, not just the final result. Furthermore, these peptide binding data can not be readily interpreted towards the interaction sites on the folded proteins. It is known that both TF and ClpX bind peptides and at least some of the observed peptide interactions may thus not occur in the folded proteins when the respective peptide is buried as part of the structure.

2.) The SPR experiments use domain constructs of TF termed TF_N, TF_P and TF_A. It is unclear from the manuscript what exactly these constructs are (residue numbers). The NMR experiments use the constructs TF_N (1-117) and TF_PA (113-432). Those domain boundaries are however in disagreement with Figure 1A and Figure 5A. This situation needs to be clarified, see also point 10 below. If the domains are defined by the colors on the left hand side, then the experiment is problematic: TF_N contains a long extension that is aggregation- and degradation-prone and TF_A will not be folded. The blue part is entirely missing.

3.) The experiments in Figure 6A show the analyzed data for 8 selected mutants. The authors need to show the underlying raw data (gels) and the degradation curves for all 38 mutants (in Supplementary) so that readers can judge on the quality of the data and conclusions. It will be also interesting to see the variation in the mutants that do not have an effect.

4.) The authors suggest that a direct interaction between TF and CLpX plays a role for the functional coupling. If this was the case, the same mutants that abrogate the effect (Figure 6) should be tested in the SPR experiments. If the interaction plays a role, then these mutations should also abolish the interaction.

5.) Experiments in Figure 6B require as an additional reference point the activity of ClpX(F16W) and ClpX(I46K) without TF.

6.) The NMR methods section should report, which assignment were used for the analysis (BMRB ID). Furthermore, one of the authors previously reported that TF forms a dimer with a KD in the low uM range. Upon titration of a binding partner / substrate, the dimer dissociates and the monomer binds

the substrate. Many of the "interaction residues" identified here by NMR (Figure 5) are located in the dimerization interface. How was the presence of the dimer-monomer equilibrium accounted for in the interpretation of the chemical shift perturbation and intensity changes? This should be clearly explained, perhaps with additional Supplementary Figures and control titrations with a TF client.

7.) It is unclear to this referee how it is possible that each of the three domains bind with affinities of ~ 10 uM, 10 uM and 0.2 uM (Table in Fig. 5A) and the full-length protein also has only 10 uM affinity to ClpX. The affinity should be at least as strong as the strongest of the individual ones and by avidity it would usually be expected to be even tighter binding. This in particular since ClpX is a hexamer that offers the same binding sites six times, and since the interaction is highly dynamic as the authors suggest.

8.) The experiments in Figure 4A require independent replicates. In particular, the outcome of the experiment at the bottom (NlO-S7 + TF) looks very odd: A high intensity jump from 0 to 0.5 h and then essentially a constant signal until 4 h. I really do not see how a quantification of these gel bands would lead to the curve shown on the right. e.g. the band at 0.75 h is clearly weaker than the one at 1 h next to it.

9.) It is not explained why TF protects degradation of HV-lO (top experiment in Figure 4A). Actually, this data point speaks against the presence of a specific mechanism and would rather point to an indirect interaction, such as via the substrate.

10.) The experiments shown in Figure S3B require independent replicates (usually in triplicate). And in these experiments I can actually clearly see an effect of the individual domains, unlike claimed in the corresponding text. Also in this experiment, the domain boundaries are undefined. If the domains are defined by the colors on the left hand side, then the entire experiment is pointless: TF_N contains a long extension that is aggregation- and degradation-prone and TF_A will not be folded. The blue part is entirely missing.

11.) The in vitro assay in Figure 1G shows that TF stimulates the ATPase activity of ClpX, even in the absence of substrates. This stimulation is not taken into account into the final functional model. Could it be that this effect alone is responsible for many of the in vitro effects (which are small)?

12.) The authors suggest that the interaction is a specific, but dynamic interaction between TF and ClpX. How does this model account for the substrate-specificity?

Taken together, while I find the topic per se interesting, I am not fully convinced about the conclusions of the work. The individual experiments do not result in a coherent picture about the mechanism of this interaction and do not point to a clear functional coupling of the two proteins. It remains unclear, how exactly this interaction is mediated and while some data points point to a tight direct interaction, others point to a rather indirect mechanism. For a majority of substrates (Figure 1), the interaction has no effect and it remains unclear how the overall mechanism could be subtrate-specific. Given these uncertainties and the substantial technical points mentioned above, I see a revised version of the manuscript better suited to a more specialized journal.

Reviewer #2 (Remarks to the Author):

The Manuscript by Rizzolo et al on “Functional cooperativity between the trigger factor chaperone and the ClpXP proteolytic complex” is a very well designed, executed and very comprehensive study showing that Trigger factor (TF) (which is a ribosome associated chaperone (mostly interacting with nascent peptide chains)) is also active as an adaptor protein, allowing and enhancing the specific degradation of a number of well-known ClpXP protein substrates. The study was triggered by the apparent co-localization of the genes encoding TF, ClpX, ClpP and Lon in most bacterial genomes,

which strongly suggests a functional interaction.

The authors clearly demonstrate in the E. coli system that TF can act as an adaptor protein targeting a known ClpX substrate lambda O for ClpXP degradation by a nice set of in vitro and in vivo experiments (Fig 1 & 2 & suppl Fig.). Such a TF mediated ClpXP dependent degradation was also observed in conjunction with other more specialized adaptor proteins such as SspB or RssB involved in protein quality control related general proteolysis of SsrA-tagged unfinished polypeptides (generated by stalled ribosomes) and regulatory proteolysis of RpoS (the general stress sigma factor).
A very interesting experiment also suggest that TF, in organisms lacking the more proteobacteria specific SspB, can by itself enhance the degradation of SsrA-tagged proteins (Fig 7F).

All the important aspects of adaptor enhanced substrate protein degradation by AAA+ protease complexes were comprehensively addressed in this study by various biochemical and biophysical experimental approaches (both qualitative and quantitative). The authors were able to characterize the recognized substrate degron (Fig 4), and could analyze and map specific interactions of TF and ClpX (Fig 5 & 6) in detail. Based on their experiments they can suggest a model for the mechanism also in respect to co- and post-translational states of the new TF adaptor protein function (Fig 7).

I believe that this is a really exciting and groundbreaking study, uncovering really new aspects about the possible mechanisms of monitoring and facilitating early protein quality control by ribosome associated chaperones. Thereby TF which monitors and interacts with the nascent polypeptide chains emanating from the ribosome is directly involved in the very early decision to recognize and target the portion of not well folding or not fully translated marked polypeptide chains for degradation by ClpXP.

Additional question and comments

-The authors demonstrate that the interaction of TF also induces the ATPase of ClpX and the experiments strongly suggest that TF itself is not a substrate of ClpXP.
In their analysis of the specific interaction and the structure function analysis it is not really mentioned whether also the ClpX ATPase was measured or an ATPase induction was observed. One could imagine that by specific interactions of TF (with or without substrate?) with the ZBD or other parts of ClpX a concurrent ATPase induction is part of the targeting and possibly activation mechanism. Or it could be that an ATPase induction of ClpX is maybe just caused by the enhanced ClpX activity of substrate recognition, unfolding and transfer to ClpP?

-The controlled degradation of RpoS during exponential growth is prominent part of many mechanism to allow a stabilization of this alternative sigma factor when nutrients become limited, the cells stop growing and additional environmental stresses can or are arising.
Maybe question can be discussed a little more is how the authors imagine that TF adaptor activity integrates into the logic of regulatory proteolysis of RpoS during the normal exponential growth phase?
It is known that the rpoS gene encodes many rare codons (Kolmsee & Hengge (pubmed ID 21788735)), which could be part of the connection between regulatory proteolysis and translation control? Could TF activity more strongly involved in the general downregulation of translation at the entry into stationary phase, and could therefore TF become titrated away from the RpoS proteolysis? Or is TF only enhancing the proteolysis after the phosphorylation of RssB results in the interaction with RpoS that exposes the degradation tag, which is subsequently also recognized by TF, as discussed in the manuscript (Fig 3). But this would suggest that regulatory proteolysis is controlled by the other adaptor and only enhanced by TF?

-Cyclomarin is a very interesting antibiotic, known to interact with the NTD of ClpC1 of Mtb and possibly deregulating otherwise controlled substrate selection and ClpC1P1/2 activity with toxic effects, resulting in cell death. However, the NTD of ClpC is not at all related to the NTD of ClpX. Is it known if ClpX is also a known target for this antibiotic??? For me many more questions come up and I

do not understand how the presented in vitro data on the interaction of Cyclomarin to the NTD of ClpX could fit into this story. I find it very confusing and not at all related to the discovery that TF can also act as an adaptor protein.
Therefore, I would suggest to leave this part about cyclomarin out of this manuscript. It can easily become part of another new story which could also address the many other questions about this observation, which are not that related to TF.

Reviewer #3 (Remarks to the Author):

In this mansucript by Rizzolo et al., the authors propose that trigger factor (TF) forms a specific interaction with ClpX, to enhance the ClpPX-mediated turnover of specific substrates. The idea that TF works cooperatively with ClpPX (based on its conserved gene organisation) has long been speculated (first proposed by Krandror et al., 1995), however to date no evidence for a direct interaction between ClpX and TF has been described.

In this manuscript the authors present some potentially interesting data to suggest a functional link between TF and ClpPX, and although the in vivo data showing the importance of TF in the ClpPX-mediated turnover of lambda-O is convincing, much of the data surrounding this finding are far less compelling, providing only weak evidence for a specific functional interaction between TF and ClpX. The vast majority of their data is consistent with a substrate-like interaction between the two proteins. The authors need to provide additional evidence to strengthen their conclusion that TF promotes degradation through specific functional interaction with ClpX and rule out that the interaction between ClpX and TF (as defined in their manuscript) is not simply a substrate binding interaction.

Specific comments
1. The in vitro degradation data provided in Figure 1 show only very small differences, with mostly large and overlapping error bars. The authors should provide some statistical analysis (of the rates of degradation) to strengthen the interpretation of these data. This also applies to data presented in Figure 3D.
2. Fig 1C – is the TF enhanced turnover of GFP-ssrA (in the presence of SspB) significant? If the TF-enhanced turnover is mediated through a specific interaction with ClpX, then the authors should be able to confirm this using a specific ClpX or TF mutant that is unable to interact with its partner. – this loss in activity should be specific to the enhance degradation and not other activities of TF or ClpX. This should be clearly demonstrated with specific control experiments (which are currently lacking in the manuscript)
3. To ensure that TF binding to ClpX is not simply a substrate-like interaction, the authors should include an additional control (comparing the stimulation of ClpX ATPase activity (Fig 1F/G) in the presence of a known ClpX substrate). This should also include a titration of the TF mutant(s) which are unable to enhance degradation.
4. I have two concerns about the quantitation. There appears to be a systematic difference between the quantitation and the representative gels (w.r.t the rate of change, compare data in Figure 4A). Can the authors try a different quantitation program (or the method) to obtain more representative quantifications of their data. The second concern relates to the starting point for each quantification. What is the authors definition of relative change? It appears that the authors use different meanings for term relative. In figure 2, the amounts of lambda-O are relative to the delta-tig experiment, hence levels in the wild type experiment start at for example (0.4 or 0,6), while in Figure 4 and 6 the amount appears to be relative to the first time point (i.e. t=0). This is important for determining the rates of degradation (e.g. in Figure 2C and 2D). Indeed from the quantitation of data in Fig 2D, the rates of degradation appear to be similar (within experimental error), and only vary in the starting point of each experiment. The authors should calculate the rates of degradation from revised quantification in Fig 2D.

5. The authors should provide the primary data for peptide library binding (in Supplementary data). The presentation of binding motifs and the explanation/relevance of these motifs needs clarification. The significance underlined regions within a motif is unclear. The motifs appear to be identical to the proposed substrate binding motif for TF (Patzelt et al., 1999 – ref #6). Hence it appears these data are consistent with substrate binding rather than docking to a functional site. The authors should examine the peptide binding using a substrate binding defective TF mutant or PPIase mutant, to clarify which regions are involved in "substrate-binding" and which are for co-factor "docking".
6. The authors model, suggest that TF docks at numerous sites on ClpX to facilitate enhanced degradation of lambda-O. Each site, presumably contributing only a small amount to the interaction. However, fig 6 shows numerous single point mutants at multiple different site are sufficient to completely inhibit the TF-enhanced degradation by ClpX. What is the authors explanation for this?
7. The authors also identified specific mutants (raised above) however additional control experiments are required to demonstrate that this is not due to a loss of all TF (or ClpX) activities / substrate binding. Addition control experiments with ClpX mutants should include control substrates that are not affected by the mutation. Figure 6B should also include a control demonstrating that substrate turnover by each mutant is not compromised (in the absence of TF)
8. The role of CymA appears tacked on to the manuscript. CymA is shown to interact with ClpX, yet has no effect on ClpX-mediated turnover. Does CymA inhibit any ClpX substrates (presumably it should inhibit substrate that interact with the ZBD). Figure 6E – shows a strong inhibitory effect of CymA on TF-enhance degradation of lambda-O. However the authors have not examined the binding of CymA with TF directly? The authors should examine ITC between TF and CymA. If there is an interaction between TF and CymA does this interaction inhibit binding of all substrates to TF?
9. The presentation of the HV-ClpX and cleaved ClpX is ambiguous. Fig 7 D contains 2 protein bands, however they are labelled differently to the other gels (Fig 7B and C). Why does the "truncation" represent the cleaved protein in one gel and uncleaved in another gel?
10. What do the 2 bands in Figure 7F (GFP-ssrA) represent?
11. Why is full-length TF required for the interaction with ZBD of ClpX? This does not seem to fit to the authors model.

Methods
concentration of substrate added to degradation assays not provided
Western verification of strains should be included in Supp figures
Details of TEV and His-SUMO removal (after cleavage) should be included
Gel filtration buffer details missing
SPR performed at room temperature – actual temperature should be included
Cyclomarin (Novartis) – provide catalogue number, or reference/method of production
protease inhibitor cocktail 25x from Sigma – which cocktail, explain meaning of 25x
0.25x FLAG peptide – details are missing (concentration / amount)
1:1000 dilution of RNAse inhibitor - provide details
Was nucleotide included in SPR experiments?

Additional concerns about supplementary figures
Figure S2
A – starting levels of MuA, vary dramatically from one experiment to the other, also significantly different to other substrates i.e. lambda-O, GFP-ssrA. What concentration of substrate was used for each experiment and why is the apparent substrate concentration different for each substrate?
B-F. the effect of TF on the experiments provided is unclear. Quantitation of (multiple) degradation experiments should be performed
C - TnaA degradation was not observed (The authors state that TF had no effect on the degradation of TnaA, but no degradation of TnaA was observed). This must be revised.

Figure S3
Concentration of substrate added to degradation assays is unclear. Why are some experiments

analysed by Western and others by Coomassie staining. Have different substrate concentrations been used? If so why?

Figure S4
Control experiments are missing from cell viability assays. From the data provided it appears that delta-tig is more sensitive (than WT) at 30 C. This is consistent with published findings and is due to the increased aggregation of newly synthesised proteins in delta-tig cells. It is not surprising that the tig/sspB double deletion strain is more sensitive. The authors should also include additional controls, tig/sspB double deletion complemented with SspB. tig/sspB double deletion complemented with control protein (e.g. dnaK), to address the role of prevention of aggregation in the TF-enhanced degradation.

Figure S10
All mutant data should be included in supp figure.
10S B- the gel is over-destained – extremely difficult to evaluate.

Response to reviewers

REVIEWER 1

General comments

1. *The manuscript by Rizzolo et al investigates a functional cooperativity between the chaperone trigger factor (TF) and the ClpX protease. Based on the initial observation that the genes for TF and ClpX are organized in the same operon for many species, the work investigates with a series of biochemical, biophysical, structural and in cell experiments in how far the two proteins could interact physically with each other and which functional consequences this may have. The work consists of an impressively large collection of different assays and experiments, most of which point to the existence of some kind of interaction. The conclusion by the authors is that TF interacts with ClpX via multiple sites in a dynamic fashion. In general, the Figures are of high quality and the experiments rather well document (with some exceptions, see below).*

We thank the reviewer for his/her positive comments.

2. *When investigating this question about a functional coupling between TF and ClpX, one should keep in mind that it is per se not surprising that TF and ClpX can exhibit cooperative effects, since they both are well known to interact with unfolded and partially folded proteins and peptides. The crucial point is therefore whether any observed effect is necessarily based on a direct interaction between TF and ClpX or rather an indirect effect, such as mediated through their respective interactions with the clients that can lead to sequestering / protection / competition effects.*

The surprising finding in our study is that TF can modulate the degradation rate of ClpXP susbtrates by directly binding to ClpX rather than indirectly by acting on the substrate alone. We show the direct binding between TF and ClpX via ATPase activity assays, NMR, SPR, and mutagenesis. Evidence of direct effect:

- TF directly binds and enhances ClpX ATPase activity requiring the ZBD of ClpX (Figure 1D,E).
- TF directly binds and modifies the movement of the ZBD_2s, a well-established substrate binding site of ClpX, towards the catalytic chamber of ClpP (Figure 3).
- TF directly binds ClpX as shown by NMR (Figure 4, S9-S11) and SPR (Figure S6).
- TF mutations are capable of abolishing ClpXP degradation enhancement effect of the λO substrate yet do not affect TF chaperone function (Figure 5B-D).

Together, our results show, using a variety of approaches, that TF directly interacts with ClpXP and can selectively enhance the degradation of substrates.

Specific comments

1.1. *Figure S6A shows the final results of a either TF or ClpX binding to a peptide binding array of the respective other protein. For this experiment, the underlying data needs to be shown in suitable format, not just the final result.*

The primary data for the peptide array experiment are now included (Figure S13).

1.2. *Furthermore, these peptide binding data can not be readily interpreted towards the interaction sites on the folded proteins. It is known that both TF and ClpX bind peptides and at least some of the observed peptide interactions may thus not occur in the folded proteins when the respective peptide is buried as part of the structure.*

We respectfully disagree. Mapping binding epitopes onto the corresponding 3D structure is a common presentation of peptide binding data (e.g. Patzelt et al., 2001). In Figure 5A (right), we mapped TF binding sites onto a surface model of the ClpX AAA+ hexamer structure. Only binding epitopes that are exposed on the surface of the ClpX AAA+ hexamer are seen. Interactions with buried peptides that might be exposed in an unfolded ClpX protein are hidden. So, the argument that some of the interactions might not be observed in the folded protein (or rather ClpX AAA+ hexamer) are not warranted. The same applies for TF (Fig. 5A - left). The figure shows a surface model of one folded TF structure. Although alternative TF conformations may exist, only motifs exposed on the surface of the folded TF structure are visible and are colored. Finally, please note that this approach was used as part of many other approaches to map the interaction between TF and ClpX.

2. *The SPR experiments use domain constructs of TF termed TF_N, TF_P and TF_A. It is unclear from the manuscript what exactly these constructs are (residue numbers). The NMR experiments use the constructs TF_N (1-117) and TF_PA (113-432). Those domain boundaries are however in disagreement with Figure 1A and Figure 5A. This situation needs to be clarified, see also point 10 below. If the domains are defined by the colors on the left hand side, then the experiment is problematic: TF_N contains a long extension that is aggregation- and degradation-prone and TF_A will not be folded. The blue part is entirely missing.*

We thank the reviewer for catching this. It was a mistake in coloring the TF domains. We modified Figures 1A and S4A accordingly. The domain boundaries are now consistent throughout the paper. TF domain boundaries are now in line with those used in our constructs. Please note that we have removed the SPR results using TF domains since the interpretation of those data was an issue.

3. *The experiments in Figure 6A show the analyzed data for 8 selected mutants. The authors need to show the underlying raw data (gels) and the degradation curves for all 38 mutants (in Supplementary) so that readers can judge on the quality of the data and conclusions. It will be also interesting to see the variation in the mutants that do not have an effect.*

As requested by the reviewer, we have included the underlying raw data for the eight selected mutants in Figure S14. However, we do not think that showing 30+ gels of negative mutants (i.e. those that behaved like WT TF and continued to enhance the degradation of the λO substrate) plus their respective replicates is necessary or useful. The manuscript is already quite large.

4. *The authors suggest that a direct interaction between TF and ClpX plays a role for the functional*

coupling. If this was the case, the same mutants that abrogate the effect (Figure 6) should be tested in the SPR experiments. If the interaction plays a role, then these mutations should also abolish the interaction.

A direct binding SPR assay would only yield incomplete information about the effect of the candidate mutants since, as evidenced by our SPR, NMR and peptide array results (Figures S6, 4, S7-S11, 5A), the interactions between TF-λO and TF-ClpX involve multiple binding sites and many of them overlap. In addition, we show that ZBD is the site of interaction for both substrate and TF arm 2 (Figures 5E,F, 4A,D). We also provide new data to show that candidate mutations do not affect TF chaperone activity (Figure 5D). In addition, our candidate mutants on the hinge region suggest that TF movement is critical for the enhancement effect.

5. *Experiments in Figure 6B require as an additional reference point the activity of ClpX(F16W) and ClpX(I46K) without TF.*

As requested by the reviewer, we have included these reactions in Figures 5E and S14D. It is important to add that this is consistent with our previous published results (PMID: 22360725).

6. *The NMR methods section should report, which assignment were used for the analysis (BMRB ID). Furthermore, one of the authors previously reported that TF forms a dimer with a KD in the low uM range. Upon titration of a binding partner / substrate, the dimer dissociates and the monomer binds the substrate. Many of the "interaction residues" identified here by NMR (Figure 5) are located in the dimerization interface. How was the presence of the dimer-monomer equilibrium accounted for in the interpretation of the chemical shift perturbation and intensity changes? This should be clearly explained, perhaps with additional Supplementary Figures and control titrations with a TF client.*

The following assignments were used in the analysis.
TF: BMRB Entry 19835
ClpX ZBD: BMRB Entry 5665
The information is included in the methods section.

As the reviewer pointed out, the binding sites on TF used for the interaction with ClpX largely overlap with the dimer interfaces (Figure S12C). This data suggest that TF monomer binds to ClpX. To avoid possible resonance perturbations induced by the monomer-dimer transition, all of the NMR titration experiments in this study were performed for the isolated domains, TF_{PA} and TF_{N}, which were both confirmed to exist as monomers in the previous study (PMID: 24812405). Thus, the perturbations shown in this study are derived from the direct interaction with the titrant. We have added the above explanation in the Result section.

7. *It is unclear to this referee how it is possible that each of the three domains bind with affinities of ~ 10 uM, 10 uM and 0.2 uM (Table in Fig. 5A) and the full-length protein also has only 10 uM affinity to ClpX. The affinity should be at least as strong as the strongest of the individual ones*

and by avidity it would usually be expected to be even tighter binding. This in particular since ClpX is a hexamer that offers the same binding sites six times, and since the interaction is highly dynamic as the authors suggest.

As mentioned previously, we have decided to remove the individual domain TF dataset from our SPR data because of issues in interpreting the data (Figure S6). However, this can be explained clearly by TF dimerization: All of the TF domains exist as monomer in solution (Saio et al. 2014 Science), and the NMR perturbation mapping suggests that the binding sites for ClpX are largely buried in the TF dimer (Saio et al. 2018 eLife). Thus, TF is expected to monomerize upon binding to ClpX. Therefore, the competition between dimerization and ClpX binding can explain the apparent mismatches in the affinity.

8. *The experiments in Figure 4A require independent replicates. In particular, the outcome of the experiment at the bottom (NlO-S7 + TF) looks very odd: A high intensity jump from 0 to 0.5 h and then essentially a constant signal until 4 h. I really do not see how a quantification of these gel bands would lead to the curve shown on the right. e.g. the band at 0.75 h is clearly weaker than the one at 1 h next to it.*

As explained in the figure legend, the experiments were performed at least three times and the average of the band intensities as well as the standard deviations were plotted. A better representative gel picked from the independent replicates is now shown. The size of λO-S7 protein is ~25kDa and runs very close to the ClpP band ~22kDa, therefore, we performed western blots using anti-S7. We modified the Figure 4D legend to state that detection was done by western blots.

9. *It is not explained why TF protects degradation of HV-lO (top experiment in Figure 4A). Actually, this data point speaks against the presence of a specific mechanism and would rather point to an indirect interaction, such as via the substrate.*

We respectfully disagree. This result supports our argument that TF cannot force the degradation of substrates that do not have a ClpX recognition sequence, but rather TF increases the rate of degradation of substrates that have such a recognition sequence. While HV-λO substrate is not significantly degraded by ClpXP because it is unable to be efficiently recognized by ClpX, it is able to bind to TF, which results in a slower degradation rate. In the current version, we show additional evidence for the TF-λO interaction by NMR (Figures S7 and S8).

10. *The experiments shown in Figure S3B require independent replicates (usually in triplicate). And in these experiments I can actually clearly see an effect of the individual domains, unlike claimed in the corresponding text. Also in this experiment, the domain boundaries are undefined. If the domains are defined by the colors on the left hand side, then the entire experiment is pointless: TF_N contains a long extension that is aggregation- and degradation-prone and TF_A will not be folded. The blue part is entirely missing.*

The issue of TF domain boundaries has been clarified in comment 2 above. We did not see a significant effect in the independent replicate degradation assays with the indicated TF domains.

As mentioned in the text, a representative gel from at least three replicates is shown. There is no significant enhancement of degradation by these domains. This is also consistent with the observation that the domains show no significant effect on ClpX ATPase (Figure S4B).

***11.** The in vitro assay in Figure 1G shows that TF stimulates the ATPase activity of ClpX, even in the absence of substrates. This stimulation is not taken into account into the final functional model. Could it be that this effect alone is responsible for many of the in vitro effects (which are small)?*

We have performed ATPase assays in the presence of substrate to complete the results shown in Figures 1D,E. TF enhances the ATPase activity of ClpX and this is further increased in the presence of substrate. The stimulation requires the presence of ZBD. In addition, we propose a clear functional model of how TF affects ClpX conformation whereby TF promotes the proximal state of ZBD (Figure 3). The stimulation of the ClpX ATPase activity by TF is a consequence of this effect on ClpX conformation. Furthermore, we show that ClpX hexamers with some of the subunits missing ZBD degrade λO at a faster rate (Figure 3E). We also show that other ClpX adaptors, such as SspB and RssB, also enhance λO degradation (Figure S15). Hence, we are proposing a clear mechanism for how TF enhances substrate degradation by ClpXP.

***12.** The authors suggest that the interaction is a specific, but dynamic interaction between TF and ClpX. How does this model account for the substrate-specificity?*

We are not clear what the reviewer is asking about. The substrate specificity arises due to the fact that the substrate requires an interaction with ZBD in ClpX or requires the ZBDs to be moved away for faster degradation rate. The substrate also needs to bind to TF. As we mention on page 28: "A nascent polypeptide chain exiting the ribosomal exit tunnel would first interact with TF_A and then be transferred to the ZBD or AAA+ domain of ClpX via their respective recognition sequence (Figure 7C, left panel)." also in the same page: "It is important to note that for each ClpX ring, there are six AAA+ domains and three ZBD_2 that have potential binding sites for TF and for substrates.".

REVIEWER 2

1. *I believe that this is a really exciting and groundbreaking study, uncovering really new aspects about the possible mechanisms of monitoring and facilitating early protein quality control by ribosome associated chaperones. Thereby TF which monitors and interacts with the nascent polypeptide chains emanating from the ribosome is directly involved in the very early decision to recognize and target the portion of not well folding or not fully translated marked polypeptide chains for degradation by ClpXP.*

We thank the reviewer for his/her positive comments.

2. *The authors demonstrate that the interaction of TF also induces the ATPase of ClpX and the experiments strongly suggest that TF itself is not a substrate of ClpXP. In their analysis of the specific interaction and the structure function analysis it is not really mentioned whether also the ClpX ATPase was measured or an ATPase induction was observed. One could imagine that by specific interactions of TF (with or without substrate?) with the ZBD or other parts of ClpX a concurrent ATPase induction is part of the targeting and possibly activation mechanism. Or it could be that an ATPase induction of ClpX is maybe just caused by the enhanced ClpX activity of substrate recognition, unfolding and transfer to ClpP?*

As shown now by the new data in Figures 1D,E, we have demonstrated that TF can stimulate ClpX ATPase activity with and without the presence of substrate. Furthermore, this effect requires the presence of ZBD. We explicitly show that TF binds ZBD (Figures 4, 5, S9, S10). Hence, we argue that the specific interactions of TF (with or without substrate) with the ZBD cause ATPase enhancement as part of the targeting and activation mechanism. We have included the ATPase stimulation effect by TF in our final model described on page 28 of the manuscript.

2. *The controlled degradation of RpoS during exponential growth is prominent part of many mechanism to allow a stabilization of this alternative sigma factor when nutrients become limited, the cells stop growing and additional environmental stresses can or are arising. Maybe question can be discussed a little more is how the authors imagine that TF adaptor activity integrates into the logic of regulatory proteolysis of RpoS during the normal exponential growth phase? It is known that the rpoS gene encodes many rare codons (Kolmsee & Hengge (pubmed ID 21788735), which could be part of the connection between regulatory proteolysis and translation control? Could TF activity more strongly involved in the general downregulation of translation at the entry into stationary phase, and could therefore TF become titrated away from the RpoS proteolysis? Or is TF only enhancing the proteolysis after the phosphorylation of RssB results in the interaction with RpoS that exposes the degradation tag, which is subsequently also recognized by TF, as discussed in the manuscript (Fig 3). But this would suggest that regulatory proteolysis is controlled by the other adaptor and only enhanced by TF?*

We appreciate the insightful comment from the reviewer regarding the selective degradation of the RpoS protein enhanced by TF. As we show in Figure 6F,G, RpoS degradation enhancement was only observed when the cognate adaptor RssB was present. Intriguingly, this points to another

regulatory condition for RpoS ClpXP-mediated degradation in the cell. Indeed, during our revisions for this manuscript a study came out by Micevzki et al (PMID: 32316259) demonstrating the mechanism of RpoS degradation by ClpXP modulated by RssB. We show evidence of a common mechanism for TF-ClpX and RssB-ClpX in RpoS degradation. Hence, TF may be an ancestral adaptor of ClpX. Indeed, more work needs to be performed to fully understand the interplay of RssB, TF, RpoS and ClpXP in the cell for proper RpoS function at different bacterial growth conditions. However, we believe that this is beyond the scope of this manuscript.

3. *Cyclomarin is a very interesting antibiotic, known to interact with the NTD of ClpC1 of Mtb and possibly deregulating otherwise controlled substrate selection and ClpC1P1/2 activity with toxic effects, resulting in cell death. However, the NTD of ClpC is not at all related to the NTD of ClpX. Is it known if ClpX is also a known target for this antibiotic??? For me many more questions come up and I do not understand how the presented in vitro data on the interaction of Cyclomarin to the NTD of ClpX could fit into this story. I find it very confusing and not at all related to the discovery that TF can also act as an adaptor protein. Therefore, I would suggest to leave this part about cyclomarin out of this manuscript. It can easily become part of another new story which could also address the many other questions about this observation, which are not that related to TF.*

We agree with the reviewer. We have removed the cyclomarin story from our manuscript.

REVIEWER 3

General Comment

In this manuscript the authors present some potentially interesting data to suggest a functional link between TF and ClpPX, and although the in vivo data showing the importance of TF in the ClpPX-mediated turnover of lambda-O is convincing, much of the data surrounding this finding are far less compelling, providing only weak evidence for a specific functional interaction between TF and ClpX. The vast majority of their data is consistent with a substrate-like interaction between the two proteins. The authors need to provide additional evidence to strengthen their conclusion that TF promotes degradation through specific functional interaction with ClpX and rule out that the interaction between ClpX and TF (as defined in their manuscript) is not simply a substrate binding interaction.

We respectfully but strongly disagree with this reviewer. The reviewer seems to be ignoring most of our results and the multiple methods that we used to specifically show that the TF-ClpX interaction is not a substrate interaction but rather an interaction with TF acting as an adaptor.

If we define an adaptor for ClpX as a protein that enhances the degradation of specific substrates without itself being degraded, then TF fits that definition (Figure S2F). However, some adaptors do indeed get degraded as well – however, TF does not.

Using many approaches, we show that TF binds to ClpX to enhance substrate degradation without TF itself being degraded (Figure S2F). The NMR data show the binding sites between TF and ClpX (Figures 4, S9,S10, S11). As shown in Figures 5B,E, mutations on TF and ClpX are found to disrupt the enhancement in the degradation of the λO substrate. In addition, we provide new data that such mutations do not affect TF chaperone activity (Figure 5D). Furthermore, we show TF-mediated stimulation of ATPase activity of ClpX in the presence and absence of substrate (Figures 1D,E). ClpA ATPase activity was not changed in the presence and absence of TF or substrate. These results demonstrate a specific functional interaction of TF with ClpX. Furthermore, we now have a proposed mechanism of TF adaptor function (Figure 3, S5E, S15) whereby TF binds to one ZBD_2 on ClpX allowing the other ZBD_2s, which could be loaded with substrates, easier access to the pore of the ClpX hexamer to start the translocation of the substrates into ClpP

Together, all these results clearly argue that TF interaction with ClpXP is not as a substrate but rather as an adaptor protein.

Specific comments

1. *The in vitro degradation data provided in Figure 1 show only very small differences, with mostly large and overlapping error bars. The authors should provide some statistical analysis (of the rates of degradation) to strengthen the interpretation of these data. This also applies to data presented in Figure 3D.*

As requested by the reviewer, we have performed t-test statistical analysis on the graphs shown in (new) Figure 1B and Figure 6A. We disagree with the reviewer's statement that the differences are small. The differences are significant and can be easily seen by eye (Figure S2A), especially for λO.

2. *Fig 1C – is the TF enhanced turnover of GFP-ssrA (in the presence of SspB) significant? If the TF-enhanced turnover is mediated through a specific interaction with ClpX, then the authors should be able to confirm this using a specific ClpX or TF mutant that is unable to interact with its partner. – this loss in activity should be specific to the enhance degradation and not other activities of TF or ClpX. This should be clearly demonstrated with specific control experiments (which are currently lacking in the manuscript)*

The curves shown in Figure 6D (previously 1C) are the averages from three independent experiments and the error bars are the standard deviations as shown. The curves show that the enhancement of GFP-ssrA by TF + SspB are indeed significant compared to no SspB and to no adaptors. As shown in Figures 5B-D, TF and ClpX mutagenesis allowed the discovery of mutations and are capable of abolishing the degradation enhancement effect, yet not disrupt chaperone function of TF or the degradation activity of ClpXP against λO (Figures 5D,E).

3. *To ensure that TF binding to ClpX is not simply a substrate-like interaction, the authors should include an additional control (comparing the stimulation of ClpX ATPase activity (Fig 1F/G) in the presence of a known ClpX substrate). This should also include a titration of the TF mutant(s) which are unable to enhance degradation.*

As requested by the reviewer, we have included the ATPase assays in the presence of the λO substrate (Figure 1D,E). Substrates and adaptor proteins have been shown to stimulate ClpX ATPase activity independently, indeed our results show a cumulative effect from both substrate and TF. Importantly, the presence of the λO substrate did not change the ATPase activity exhibited by ClpA. This highlights TF specificity for ClpX.

Note that checking the effect on the ATPase activity of ClpX by TF mutants that do not enhance λO degradation is not warranted as we show that such mutants still exhibit chaperone activity (Figure 5B-D).

4.1. *I have two concerns about the quantitation. There appears to be a systematic difference between the quantitation and the representative gels (w.r.t the rate of change, compare data in Figure 4A). Can the authors try a different quantitation program (or the method) to obtain more representative quantifications of their data.*

As requested by the reviewer, we have revised the quantification for Figure 2D (previously Figure 4A).

4.2. *The second concern relates to the starting point for each quantification. What is the authors definition of relative change? It appears that the authors use different meanings for term relative. In figure 2, the amounts of lambda-O are relative to the delta-tig experiment, hence levels in the wild type experiment start at for example (0.4 or 0,6), while in Figure 4 and 6 the amount appears to be relative to the first time point (i.e. t=0). This is important for determining the rates of degradation (e.g. in Figure 2C and 2D). Indeed from the quantitation of data in Fig 2D, the rates*

of degradation appear to be similar (within experimental error), and only vary in the starting point of each experiment. The authors should calculate the rates of degradation from revised quantification in Fig 2D.

As requested by the reviewer, we have clarified how the data were normalized. For the *in vivo* experiments, this is indicated on the y-axis of Figures 2A-C, 6C, and 7A,B. For all *in vitro* assays, band intensities are normalized relative to the amount at the starting point (T=0). We have performed the quantification again for Figure 6C. We would like to emphasize that our conclusions remain the same.

5.1. *The authors should provide the primary data for peptide library binding (in Supplementary data). The presentation of binding motifs and the explanation/relevance of these motifs needs clarification. The significance underlined regions within a motif is unclear.*

The primary data for peptide library binding are now included in Figure S13 together with a clarification of the importance of the underlined and/or highlighted amino acids. Briefly, miniaturized libraries of overlapping 12mer peptides were synthesized by walking through the amino acid sequence of *E. coli* TF or *E. coli* ClpX, advancing three (TF) or two (ClpX) amino acids at a time. Overlapping TF-derived peptides bound by ClpX AAA+ (Fig. S13A) or overlapping ClpX-derived peptides bound by TF (Fig. S13C) were grouped into motifs. For each motif, the common region in overlapping peptides (consensus) is underlined. Amino acids are highlighted in bold if a complete set of overlapping peptides belonging to this motif and featuring this amino acid were found. Single peptides bound by ClpX AAA+ are also shown but are not grouped into motifs (Fig. S13B).

5.2. *The motifs appear to be identical to the proposed substrate binding motif for TF (Patzelt et al., 1999). Hence it appears these data are consistent with substrate binding rather than docking to a functional site. The authors should examine the peptide binding using a substrate binding defective TF mutant or PPIase mutant, to clarify which regions are involved in "substrate-binding" and which are for co-factor "docking".*

The reviewer is mistaken. Identifying ClpX binding motifs by probing a TF-derived peptide array (Fig. S13A and S13B) was not subject of the Patzelt *et al.* study. Patzelt *et al.* identified amino acids frequently found in TF substrates (i.e. hot spots). This is very different to identifying binding epitopes to map the interaction between TF and ClpX AAA+ as it was done here (Fig. 5A and S13A-C). We further note that ClpX (Figure S13C) was not amongst the 20 substrate proteins analyzed in the Patzelt *et al.* study. Importantly, only two out of seven TF-bound peptide motifs featured an aromatic amino acid, and none of the peptides were enriched in positively charged amino acids, with the exception of perhaps motif 6. This argues against the notion that ClpX is a TF substrate and supports a role of TF as an adaptor for ClpX.

6. *The authors model, suggest that TF docks at numerous sites on ClpX to facilitate enhanced degradation of lambda-O. Each site, presumably contributing only a small amount to the interaction. However, fig 6 shows numerous single point mutants at multiple different site are*

sufficient to completely inhibit the TF-enhanced degradation by ClpX. What is the authors explanation for this?

As the reviewer pointed out, the TF-ClpX complex is mediated by multiple interaction sites (Figure 4 and Figure 5A), but our data also show that each of the interaction sites is essential for the function (Figure 5B). Multiple binding sites can be important to keep the two protein complexes in a specific orientation, in analogy to the interaction reported for ClpB-DnaK interaction (Rosenzweig et al. 2013 Science). Even if only one site among the multiple interaction sites are disrupted by mutagenesis, one can expect that the functional complex is no longer formed without the assistance of an essential site. This feature is different from that applies for the recognition of the unfolded substrate protein (Figure 5D) and supports the specific functional relationship between TF and ClpX.

7. *The authors also identified specific mutants (raised above) however additional control experiments are required to demonstrate that this is not due to a loss of all TF (or ClpX) activities / substrate binding. Addition control experiments with ClpX mutants should include control substrates that are not affected by the mutation. Figure 6B should also include a control demonstrating that substrate turnover by each mutant is not compromised (in the absence of TF)*

The same comment was raised by Reviewer 1, comment 5. As we mention above, we have added new data for TF mutants that do not enhance ClpXP-dependent degradation of λO but still retain their chaperone activity (Figure 5D).

8. *The role of CymA appears tacked on to the manuscript. CymA is shown to interact with ClpX, yet has no effect on ClpX-mediated turnover. Does CymA inhibit any ClpX substrates (presumably it should inhibit substrate that interact with the ZBD). Figure 6E – shows a strong inhibitory effect of CymA on TF-enhance degradation of lambda-O. However the authors have not examined the binding of CymA with TF directly? The authors should examine ITC between TF and CymA. If there is an interaction between TF and CymA does this interaction inhibit binding of all substrates to TF?*

This issue was also raised by Reviewer 2, comment 3. We have removed the cyclomarin story from our manuscript.

9. *The presentation of the HV-ClpX and cleaved ClpX is ambiguous. Fig 7 D contains 2 protein bands, however they are labelled differently to the other gels (Fig 7B and C). Why does the "truncation" represent the cleaved protein in one gel and uncleaved in another gel?*

The reviewer did not interpret the figure correctly. The top band is the tagged-ClpX(F269W) mutant and the lower is the wild type ClpX. As described on page 12-13: "A ClpX mutant unable to bind to ClpP, HV-ClpX(F269W)[30], was not significantly degraded or clipped in the presence of TF and wild-type ClpXP (Figure 3D), demonstrating that clipping is not from free HV-ClpX[29]."

10. *What do the 2 bands in Figure 7F (GFP-ssrA) represent?*

The GFP-ssrA$^{S. mutans}$ (upper band) is very close in size to the ClpP$^{S. mutans}$ (lower band). To clarify, we provide in Figure S17B a better image of the obtained results.

11. Why is full-length TF required for the interaction with ZBD of ClpX? This does not seem to fit to the authors model.

The reviewer does not clarify where he read this in our previous manuscript. We are not sure what he/she is referring to.

Methods

1. *concentration of substrate added to degradation assays not provided.*

Substrate concentrations are indicated in the methods section on page 29.

2. *Western verification of strains should be included in Supp figures*

This is now included in Figure S18.

3. *Details of TEV and His-SUMO removal (after cleavage) should be included*

This was described before in the Methods section – currently on page 31.

4. *Gel filtration buffer details missing*

This is now added to the Methods section.

5. *SPR performed at room temperature – actual temperature should be included*

This is now added on page 36.

6. *Cyclomarin (Novartis) – provide catalogue number, or reference/method of production*

This is removed from the manuscript.

7. *protease inhibitor cocktail 25x from Sigma – which cocktail, explain meaning of 25x*

This is added on page 37.

8. *0.25x FLAG peptide – details are missing (concentration / amount)*

This is added on page 38.

9. *1:1000 dilution of RNAse inhibitor - provide details*

This is added on page 38.

10. *Was nucleotide included in SPR experiments?*

Experiments were carried out with and without nucleotides. However, the data were not as 'clean'when nucleotides were added. The data shown and the K_d's listed in Figure S6 are without nucleotides.

Additional concerns about supplementary figures

1. *Figure S2:*
A – starting levels of MuA, vary dramatically from one experiment to the other, also significantly different to other substrates i.e. lambda-O, GFP-ssrA. What concentration of substrate was used for each experiment and why is the apparent substrate concentration different for each substrate?

This is typically a staining/destaining issue. As explained in the Methods section on page 35, all substrate concentrations were 3.9 µM. Although band intensities in the SDS-PAGE gels between different assays may vary, the intensities within assays, such as comparing plus/minus for a particular chaperone for a particular substrate, do not.

2. *B-F. the effect of TF on the experiments provided is unclear. Quantitation of (multiple) degradation experiments should be performed*

We respectfully disagree with the reviewer. This is not necessary because we did not see an effect. Nevertheless, all experiments were repeated more than 3 times. Quantification will not change the result as there is no significant degradation. We have already carried out a huge number of experiments for this paper.

3. *C – TnaA degradation was not observed (The authors state that TF had no effect on the degradation of TnaA, but no degradation of TnaA was observed). This must be revised.*

We did not see any degradation with this substrate. We fixed the wording.

4. *Figure S3*
Concentration of substrate added to degradation assays is unclear. Why are some experiments analysed by Western and others by Coomassie staining. Have different substrate concentrations been used? If so why?

Concentrations of substrates are given in the Methods section. We used western blotting only when protein bands overlapped with those of others in the reaction.

5. *Figure S4*
Control experiments are missing from cell viability assays. From the data provided it appears that delta-tig is more sensitive (than WT) at 30 C. This is consistent with published findings and is due to the increased aggregation of newly synthesised proteins in delta-tig cells. It is not surprising that the tig/sspB double deletion strain is more sensitive.

While the Δ*tig* cells show a bit more sensitivity at 30 °C compared to WT, there is a synthetic lethal interaction between *tig* and *sspB*. This genetic interaction is typically interpreted as indicating that both TF and SspB are acting in the same cellular pathway. The fact that the presence of TF can complement the growth defect of the Δ*tig*Δ*sspB* strain further supports our conclusion that TF has overlapping functions with SspB.

6. *The authors should also include additional controls, tig/sspB double deletion complemented with SspB. tig/sspB double deletion complemented with control protein (e.g. dnaK), to address the role of prevention of aggregation in the TF-enhanced degradation.*

This is not needed and is beyond the scope of this paper. We only want to make the point that TF can complement the growth defect of the Δ*tig*Δ*sspB* and, hence, TF has overlapping functions with SspB.

7. *Figure S10*
All mutant data should be included in supp figure.

We have included the underlying raw data for the eight selected mutants in Figure S14. However, we do not think that showing 30+ gels of negative mutants (i.e. those that behaved like WT TF and continued to enhance the degradation of the λO substrate) is necessary or useful. The manuscript is already quite large.

8. *Figure S10B- the gel is over-destained – extremely difficult to evaluate.*

We have included the revised gel (Figure S14B).

Reviewer #1 (Remarks to the Author):

The authors have revised their manuscript. I can see that a lot of work has gone into this manuscript. Unfortunately, in my eyes several central issues still remain:

- As an answer to my previous point #7, the author claim that the competition between dimerization and ClpX binding could explain the apparent mismatch in the affinity. But this is not a biophysically valid explanation. There is a mismatch between the affinities of TF to different ClpX constructs and these are affected in the same way by the auto-dimerization of TF.

- The authors did not explain how their proposed TF-ClpX mechanism can be substrate-specific (Question #12). Substrate-specific means that it affects only 2% of newly synthesized proteins as stated in abstract and shown for some substrates in Fig. 1. Why these and not others? What is recognized in those substrates?

- The SPR results in Figure S6 show that TF binds tighter to ClpX_DeltaZBD than to ClpX. This makes no sense, since the ZBD is essential for the interaction between the two proteins, as shown in Fig. 1E.

- The KD fit in Figure S3B is not legitimate, since saturation was not reached.

- The KD fit in Figure S6, top row, is not legitimate, since saturation was not reached.

- It is not explained why the effect of Delta_tig is so strong in the experiment in Figure 2A compared to Figure 2B.

- There is a statement on page 8, 3rd line, saying that if TF would act as a folding or holding chaperone, one would expect it to have either no effect or protect the substrate from degradation. But this is not a valid conclusion. As a holdase, it could also prevent the formation of local aggregation and hence make the substrate better accessible to ClpX, hence accelerating its degradation.

- The relevance of the control experiments with DnaK (Hsp70) alone is questionable, since DnaK works with co-chaperones for many, if not most, of its substrates.

- The effect of TF on ClpX is very mild, enhancing the ATPase activity by just 20% for a few substrates. I do not see a strong biological relevance in this effect.

- Both me and referee 3 have asked specifically for the raw data underlying former Figure S10. The authors do unfortunately not want to show these data. They could have shown them as review-material-only to not prolong their manuscript.

- The proposed mechanism remains vaguely defined, and the numerous affinities that are measured are not explained mechanistically, included the KDs from SPR and the fluorescence-monitored degradation assay. I do not doubt that there is some kind of transient interaction between TF, some substrates and the ClpX machinery, but from the data shown I am not convinced this interaction is anything beyond substrate-mediated or otherwise strongly relevant.

- I also realized that reviewer 3 independently shared my central concerns about the relevance of the mechanism.

Taken together, I remain unconvinced about the conclusions of the work. The individual experiments do not result in a coherent picture about the mechanism of the TF-ClpX interaction and do not point to a clear functional coupling of the two proteins. The interaction has a rather mild effect for only 2% of all newly synthesized proteins and it remains unclear how the overall mechanism could be substrate-specific.

I am assuming that Nature Communications aims at works of high impact and without uncertainties. Given the residual uncertainties and the technical points mentioned, I therefore must remain at my original assessment and see the revised version of the manuscript better suited in a more specialized journal.

Reviewer #2 (Remarks to the Author):

I am very happy with the revised manuscript.

For the discussion of the interplay between SspB and TF for the degradation of ssrA-tagged proteins it might be worth mentioning that SspB activity was initially identified in "...a high-salt wash of partially purified ribosomes..." (Levchenko et al (2000) Science 289:2354)

Reviewer #3 (Remarks to the Author):

The authors have provided new data, missing statistical information and several minor corrections to the revised manuscript. In the absence of new data they have provided suitable responses to my remaining comments. Overall, I am satisfied that their changes and responses have addressed each of my concerns raised during the initial review of this manuscript.

Reviewer 1

1. As an answer to my previous point #7, the author claim that the competition between dimerization and ClpX binding could explain the apparent mismatch in the affinity. But this is not a biophysically valid explanation. There is a mismatch between the affinities of TF to different ClpX constructs and these are affected in the same way by the auto-dimerization of TF.

We respectfully disagree with the Reviewer for the following reasons. First, as clearly mentioned in the previous rebuttal, this part of the SPR data showing the affinities of the individual TF domains against ClpX was removed from the manuscript. Second, the SPR data is not meant to discuss the difference in the affinities but to show that all of the domains of ClpX are involved in the binding to TF. As stated in the main text, the K_d obtained is an apparent dissociation constant due to the oligomeric state of ClpX. Thus, we do not discuss the differences in K_d values for the different TF and ClpX constructs. The information we obtained from the SPR experiments is that all of the ClpX domains are involved in the interaction with TF, which is highly consistent with the other experimental results including NMR and peptide array (Fig. 4, 5A). Furthermore, we consider that the SPR is showing non-stoichiometric binding of 1+ TF monomer to 1 ClpX hexamer and that other, non-binding domains could interfere with this binary interaction giving rise to the different apparent K_ds.

2. The authors did not explain how their proposed TF-ClpX mechanism can be substrate-specific (Question #12). Substrate-specific means that it affects only 2% of newly synthesized proteins as stated in abstract and shown for some substrates in Fig. 1. Why these and not others? What is recognized in those substrates?

We are not clear what the reviewer is asking about. Substrate specificity arises due to the fact that the substrate requires an interaction with ZBD in ClpX or requires the ZBDs to be moved away for faster degradation rate. As we mention on page 28: “A nascent polypeptide chain exiting the ribosomal exit tunnel would first interact with TF_A and then be transferred to the ZBD or AAA+ domain of ClpX via their respective recognition sequences (Figure 7C, left panel)” … “It is important to note that for each ClpX ring, there are six AAA+ domains and three ZBD_2 that have potential binding sites for TF and for substrates”.

In our experiments, we confirm substrate specificity with the discovery and analyses of both the endogenous substrate λO and the *in vivo* trapped substrate RpoS (Figures 1B-D, 6E-G). We show that both λO and RpoS contain similar N-terminal ClpX recognition sequences that are critical for enhancement of their ClpXP-dependent degradation by TF. Furthermore, we find that the interaction of ClpX adaptor proteins, RssB and SspB, with ZBD also causes enhancement of degradation of specific substrates. Both adaptors enhance λO degradation but not MuA degradation (Figure S15). Together, our data clearly show that the enhancement of ClpXP-dependent degradation by TF is substrate-specific.

In addition, it is important to note that mechanisms for substrate-selectivity for chaperones are not fully understood even for TF itself, which is one of the most extensively studied molecular chaperones (Patzelt et al. 2001 *PNAS*; Saio et al. 2014 *Science*). Interestingly, recent work

highlighted the importance of kinetic parameters of folding/unfolding in addition to the amino acid sequence and structure of the substrate for recognition by chaperones (Huang et al. 2016 *Nature*; Saio et al. 2018 *eLife*). Therefore, we expect that substrate selection achieved by both ClpX and TF will be complex. Here, we showed that one feature of importance is the N-terminal sequence of the substrate.

3. The SPR results in Figure S6 show that TF binds tighter to ClpX_DeltaZBD than to ClpX. This makes no sense, since the ZBD is essential for the interaction between the two proteins, as shown in Fig. 1E.

We strongly disagree with the Reviewer. Figure 1E shows ATPase activity and stimulation of ATPase activity of ClpX by TF and substrate. Figure S6 shows the binding affinity between TF and ClpX full length and its domains. Measuring binding parameters is one thing; determining ATPase activity is another thing. ATPase stimulation is presumably the result of binding and more importantly translocating substrate, which is unrelated to the binding affinity between TF and ClpX. Also, substrate was not included in the SPR experiments. In addition, throughout our work we provide robust data from NMR (Figure 4A, D), mutagenesis (Figure 5E), ATPase activity assays (Figure 1E), and ZBD proximity assays (Figure 3), to collectively conclude that TF binds to ClpX using multiple sites on both of its domains. Many approaches were used to reach our conclusion.

4. The KD fit in Figure S3B is not legitimate, since saturation was not reached.
5. The KD fit in Figure S6, top row, is not legitimate, since saturation was not reached.

As the reviewer pointed out, obtaining saturation point assures more accurate K_d fit. However, we did not obtain data points at higher TF and ClpX concentrations for two reasons: The first is that, at a higher concentrations, the proteins started to precipitate. The second, given the weaker affinity between TF and ClpX, which is often seen in the interaction between chaperones, obtaining saturation points is not always possible. For accurate interpretation of the data, we provide the uncertainty derived from the fitting (Fig. S3B, S6). This has no impact on our conclusions and does not change the results of the manuscript. Thus, we strongly disagree with the reviewer's statement that it is 'not legitimate'.

We added to the figure legends of figures S3B and S6A, the sentence "measurements at higher protein concentrations needed to reach saturation were not possible because of protein aggregation".

6. It is not explained why the effect of Delta_tig is so strong in the experiment in Figure 2A compared to Figure 2B.

This is simply due to the difference in expression of the λO protein between both *in vivo* experiments. As we describe on page 10 of the manuscript, λO is overexpressed in Figure 2A from a plasmid and degradation occurs at 37 °C. In Figure 2B, λO is expressed from a lysogenic

strain. 42 °C was used in order to induce lysogens as described in the methods. The rate of degradation is consistent with that observed previously under these conditions (PMID: 8226770).

Importantly, Figure 2 shows that λO degradation is dependent on both TF and ClpXP and requires an exposed degron. The finding is consistent with our *in vitro* observations (Figure 1). We fail to see a problem with data interpretation.

7. There is a statement on page 8, 3rd line, saying that if TF would act as a folding or holding chaperone, one would expect it to have either no effect or protect the substrate from degradation. But this is not a valid conclusion. As a holdase, it could also prevent the formation of local aggregation and hence make the substrate better accessible to ClpX, hence accelerating its degradation.

The Reviewer is misinterpreting our statement made at the beginning of the manuscript as a conclusion. It is clear that it is made to suggest a working hypothesis to be tested for the effect of TF on ClpXP-mediated degradation of model substrates (Figure 1B). We are suggesting that if a chaperone is holding a substrate, it is likely that its degradation rate will be reduced. Nevertheless, we agree that there is the possibility that TF could prevent the aggregation of substrates; however, we found no evidence of λO or GFP-SsrA aggregation under the conditions of our experiments. On page 8, we have added the following sentences:
"Alternatively, it is conceivable that TF might prevent the formation of small aggregates making substrates more accessible to ClpX and accelerating their degradation by ClpXP. However, there is no evidence of λO or GFP-SsrA aggregation under the conditions of our experiments."

8. The relevance of the control experiments with DnaK (Hsp70) alone is questionable, since DnaK works with co-chaperones for many, if not most, of its substrates.

We are not clear what the Reviewer is asking. If this question is in reference to Figure 1B, then this observation is erroneous since we in fact did include DnaJ and GrpE along with DnaK in our experiments (Figure 1B). Furthermore, Hsp70/DnaK has other known functions besides protein folding, which are independent of co-chaperones (e.g. PMID: 23650362).

9. The effect of TF on ClpX is very mild, enhancing the ATPase activity by just 20% for a few substrates. I do not see a strong biological relevance in this effect.

We strongly disagree with the Reviewer. Indeed, a similar effect is seen with the MecA adaptor protein of ClpC. ClpC is the ATPase subunit of the ClpCP protease of *Bacillus subtilis* (PMID: 10447896, PMID: 9000055).

It should be noted that what the reviewer is calling a mild effect is one of the most important features among the chaperones. Owing to the high concentration of chaperones in the cell, even a small change will drive significant perturbation to the cellular proteome. This is in contrast with, for example, signaling molecules whose activity changes by an order of 10 or more upon

stimulation. Signaling molecules are typically at very low levels in the cell and need to trigger clear-cut ON-OFF events. Thus, we argue that chaperones, whose primary function is to maintain cellular homeostasis, need a distinct standard. Therefore, 20% enhancement in ATPase activity of ClpX by TF is very significant and cannot be ignored.

10. Both me and referee 3 have asked specifically for the raw data underlying former Figure S10. The authors do unfortunately not want to show these data. They could have shown them as review-material-only to not prolong their manuscript.

We have compiled the raw images of the *in vitro* degradation assays using TF and ClpX mutants that did not significantly abolish the enhancement the ClpXP-dependent λO degradation by TF. The link is here:
https://www.dropbox.com/sh/y758suuxxd7jjq7/AAD-MnHGl2A898geJ8Jto28Xa?dl=0

Referee 3 is satisfies with the manuscript.

11. The proposed mechanism remains vaguely defined, and the numerous affinities that are measured are not explained mechanistically, included the KDs from SPR and the fluorescence-monitored degradation assay. I do not doubt that there is some kind of transient interaction between TF, some substrates and the ClpX machinery, but from the data shown I am not convinced this interaction is anything beyond substrate-mediated or otherwise strongly relevant.

Because the Reviewer is referring to the original manuscript, some of these critique points are no longer relevant and have since been addressed in the revision. The Reviewer contradicts himself/herself with the previous argument that the "effect of Delta_tig is so strong". Not only do we show a direct interaction between TF and ClpX, but we also show stimulation of the ATPase activity of ClpX by TF, which strongly supports the functional relationship between TF and ClpX. In our revised manuscript, we have shown that mutations that have negligible effect on the chaperone activity of TF itself have significant effect on protein degradation mediated by TF-ClpXP. This clearly highlights the functional relationship between TF and ClpXP and we clearly describe a detailed mechanism shown in Figures 3A and 7C elucidating the effect of TF on ClpX.

The other two reviewers no longer see an issue with the manuscript.

12. I also realized that reviewer 3 independently shared my central concerns about the relevance of the mechanism.

We disagree with the Reviewer. In our rebuttal, we propose a clear mechanism for the effect of TF on ClpX leading to enhancement of substrate degradation by ClpXP (Fig. 3A and 7C). Briefly, we propose that blocking of ZBD-dimers by the TF chaperone causes the enhancement of degradation of a substrate bound to other ZBD-dimers in ClpXP and that TF promotes the proximal (ZBD down) state of ClpX.

Reviewer 3 has no further concerns. So, this comment no longer holds true.

13. Taken together, I remain unconvinced about the conclusions of the work. The individual experiments do not result in a coherent picture about the mechanism of the TF-ClpX interaction and do not point to a clear functional coupling of the two proteins. The interaction has a rather mild effect for only 2% of all newly synthesized proteins and it remains unclear how the overall mechanism could be substrate-specific.

The reviewer is mistaken. 2% is a significant number when it comes to the function of molecular chaperones in cellular protein homeostasis. For example, only 5% of cellular proteins are found to be dependent on GroEL for their folding (PMID: 9267029). In this regard, it should be noted that, unlike TF, GroEL is an essential chaperone in *E. coli*. Another example is the finding that 5-10% of newly translated proteins interact with DnaK (PMID: 10380927). So, 2% is a significant number in this context.

14. I am assuming that Nature Communications aims at works of high impact and without uncertainties. Given the residual uncertainties and the technical points mentioned, I therefore must remain at my original assessment and see the revised version of the manuscript better suited in a more specialized journal.

Unfortunately, the reviewer seems to ignore all our data and our results. He/she keeps going back to his/her initial concerns that we have addressed and fully answered in the revised manuscript. We have addressed all uncertainties and technical issues raised by this reviewer. Our work highlights the discovery of a novel and very exciting function for TF beyond its regular chaperone function.

Furthermore, the reviewer seems to chastise *Nature Communications*, which is unwarranted and gratuitous.

Reviewer 2

I am very happy with the revised manuscript.
For the discussion of the interplay between SspB and TF for the degradation of ssrA-tagged proteins it might be worth mentioning that SspB activity was initially identified in "...a high-salt wash of partially purified ribosomes..." (Levchenko et al (2000) Science 289:2354)

We thank the reviewer for her/his comment. As requested, we added the following sentence on page 21:
"In this regard, it is important to note that SspB activity was initially identified in partially purified ribosomes[44]."

Reviewer 3

The authors have provided new data, missing statistical information and several minor corrections to the revised manuscript. In the absence of new data they have provided suitable responses to my remaining comments. Overall, I am satisfied that their changes and responses have addressed each of my concerns raised during the initial review of this manuscript.

We thank the reviewer for her/his comment.
